# Rimonabant-Based Compounds Bearing Hydrophobic Amino Acid Derivatives as Cannabinoid Receptor Subtype 1 Ligands

**DOI:** 10.1021/acsmedchemlett.3c00024

**Published:** 2023-03-09

**Authors:** Szabolcs Dvorácskó, Marilisa Pia Dimmito, Jessica Sebastiani, Giuseppe La Regina, Romano Silvestri, Stefano Pieretti, Azzurra Stefanucci, Csaba Tömböly, Adriano Mollica

**Affiliations:** † Laboratory of Chemical Biology, Institute of Biochemistry, Biological Research Centre, Temesvári krt. 62, 6726 Szeged, Hungary; ‡ Department of Medicinal Chemistry, University of Szeged, 6720 Szeged, Hungary; § Department of Pharmacy, University “G. d’Annunzio” Chieti-Pescara, Via dei Vestini 31, 66100 Chieti, Italy; ∥ Laboratory Affiliated with the Institute Pasteur Italy - Cenci Bolognetti Foundation, Department of Drug Chemistry and Technologies, Sapienza University of Rome, Piazzale Aldo Moro 5, 00185 Rome, Italy; ⊥ National Centre for Drug Research and Evaluation, Istituto Superiore di Sanità, Viale Regina Elena 299, 00161 Rome, Italy

**Keywords:** cannabinoid, rimonabant, orexigenic agent, anorexant, anti-nociception, inflammation

## Abstract

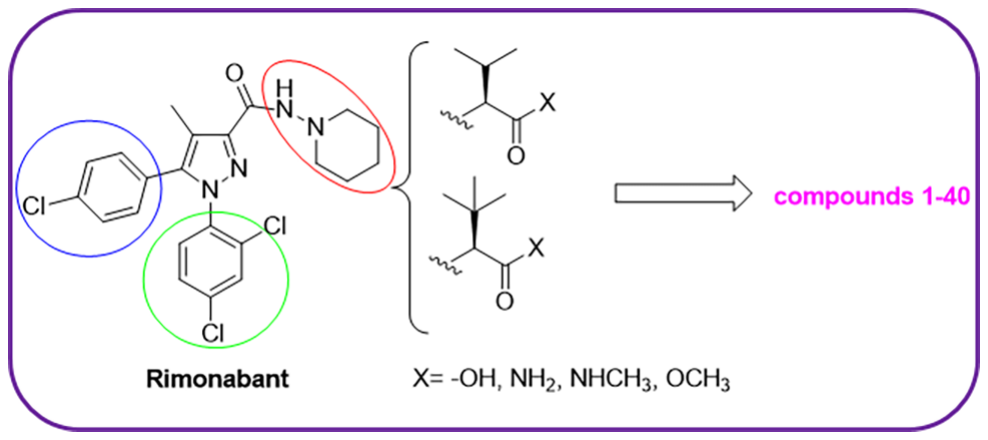

In this study, 1*H*-pyrazole-3-carboxylic acids related to the cannabinoid type 1 (CB1) receptor antagonist rimonabant were amidated with valine or *tert*-leucine, and the resulting acids were further diversified as methyl esters, amides, and *N*-methyl amides. *In vitro* receptor binding and functional assays demonstrated a wide series of activities related to the CB1 receptors (CB1Rs). Compound **34** showed a high CB1R binding affinity (*K*_i_ = 6.9 nM) and agonist activity (EC_50_ = 46 nM; *E*_max_ = 135%). Radioligand binding and [^35^S]GTPγS binding assays also demonstrated its selectivity and specificity to CB1Rs. Moreover, *in vivo* experiments revealed that **34** was slightly more effective than the CB1 agonist WIN55,212-2 in the early phase of the formalin test, indicating a short duration of the analgesic effect. Interestingly, in a mouse model of zymosan-induced hindlimb edema, **34** was able to maintain the percentage of paw volume below 75% for 24 h following subcutaneous injection. After intraperitoneal administration, **34** increased the food intake of mice, suggesting potential activity on CB1Rs.

The endogenous cannabinoid system (ECS) plays a pivotal role in feeding behavior and metabolism in the insurgence of obesity associated with metabolic risk factors, suggesting a possible correlation with cardiovascular diseases (CVDs) and type-2 diabetes.^1^ The endocannabinoid system may modulate orexigenic and anorexigenic behaviors, as described by Wilson et al., mainly via the regulation of neurotransmitters such as serotonin^2^ and norepinephrine^3^ at the presynaptic level. Activation of the cannabinoid type 1 (CB1) receptor (CB1R) in adipocytes reduces adiponectin production and promotes lipogenesis; thus, selective modulators of CB1R signaling could represent a possible strategy to avoid fat accumulation.^1^ Drugs interfering with endocannabinoid CB1R activation, mostly inverse agonists, may suppress diverse food-related behaviors. Behavioral studies involving high fat and carbohydrate diets have reveal that their consumption is influenced by CB1 inverse agonists; however, patients on such treatments have suffered several unwanted side effects, such as nausea and anxiety.^4,5^ On the other hand, the drug rimonabant (*N*-(piperidin-1-yl)-5-(4-Cl-phenyl)-1-(2,4-di-Cl-phenyl)-4-methyl-1*H*-pyrazole-3-carboxy-amide) significantly reduces body weight and waist circumference and positively influences the lipidic profile by CB1R antagonism.^6^ By blocking the CB1 activation, several mechanisms involved in weight control are effected, such as orexigenic and anorexigenic neuropeptides, hormones, mitochondrial function, and lipogenesis.^7^ These functions are largely controlled at the central level; however, the weight loss may also occur when the drugs do not cross the blood–brain barrier (BBB) efficaciously.^7,8^ This phenomenon opens the way to the development of peripheral CB1 antagonists, avoiding the side effects of central-acting drugs such as rimonabant and taranabant.^9,10^ Rimonabant is a selective CB1 inverse agonist that promotes the loss of body weight by reducing food intake through alteration of the metabolic activity in adipose tissue.^11^ In clinical trials, rimonabant ameliorated the main metabolic parameters, reducing triglycerides and body fat. However, its use was suspended in 2008 because of patients’ development of central side effects.^12^ In our recent studies, novel fubinaca–rimonabant hybrids containing the lonidamine scaffold and amidated with leucine, valine, or *tert*-leucine (*t*Leu) as an amide and an *N*-methyl amide, carboxylic acid, or methyl ester *C*-terminus were prepared via solution-phase synthesis.^13−15^ In detail, compounds containing *tert*-leucine and valine were able to exert orexant, anorexiant, or anti-inflammatory effects *in vivo* while showing diverse binding potency and efficacy versus CB receptors. In this work, we widened the structure–activity relationship (SAR) studies by synthesizing and testing 40 novel compounds, namely, **1–40**, where *tert*-leucine or valine was coupled with a series of rimonabant-mimicking scaffolds (Figure 1), with the aim to find highly potent CB1R ligands potentially useful for the treatment of inflammation-related pathologies, obesity, and anorexia. These scaffolds conserve the *N*-phenyl group and the pyrazole core, but the *p*-Cl phenyl group has been replaced with a pyrrole (**RS2689**, **RS2709**, **RS2691**, **RS2708**)^16^ or 2-Cl-pyrrole group (**RS3516**).^17^ The novel structures with *tert*-leucine or valine amino acids were variously functionalized as acid, amide, methyl ester, or *N*-methyl amide.

**Figure 1:**
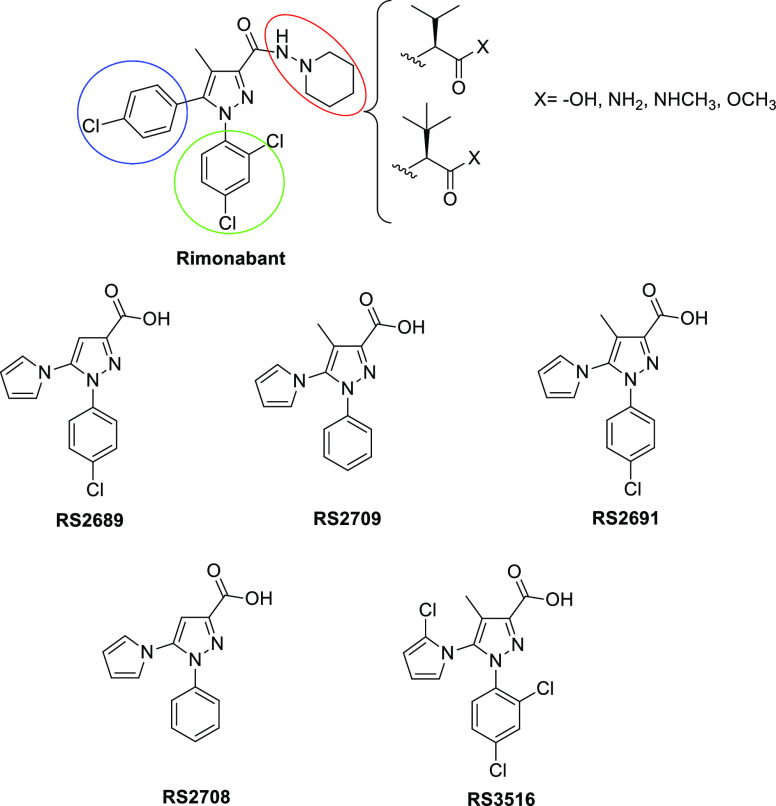
Rimonabant and **RS** scaffold structures.

The hybrid compounds were prepared by tandem solution and solid-phase synthesis (Table 1). The compounds of the series **1**–**10** were synthesized starting from 2-Cl-trityl chloride resin (loading coefficient: 1.60 mmol/g), which was coupled with Fmoc-Val-OH or Fmoc-*t*Leu-OH according to the procedure reported in the Supporting Information (SI), Scheme S1. After removal of the fluorenyl-methoxycarbonyl protecting group with 20% piperidine/DMF, the α-amino group was acylated with a series of scaffolds (**RS** series) using a HOBt/TBTU/DIPEA mixture in DMF. Acidolytic cleavage from the resin resulted in carboxylic acids **1**–**10** (Table 1). The acids were triturated in diethyl ether and then used for the preparation of their methyl esters **21**–**30** (Table 1 and Scheme S1), which were purified on silica gel. The compounds of the series **1**–**10** and **21**–**30** were obtained in good overall yields and excellent purity as detected by reverse-phase high-performance liquid chromatography (RP-HPLC) analysis (for experimental details, see the SI).

**Table 1:**
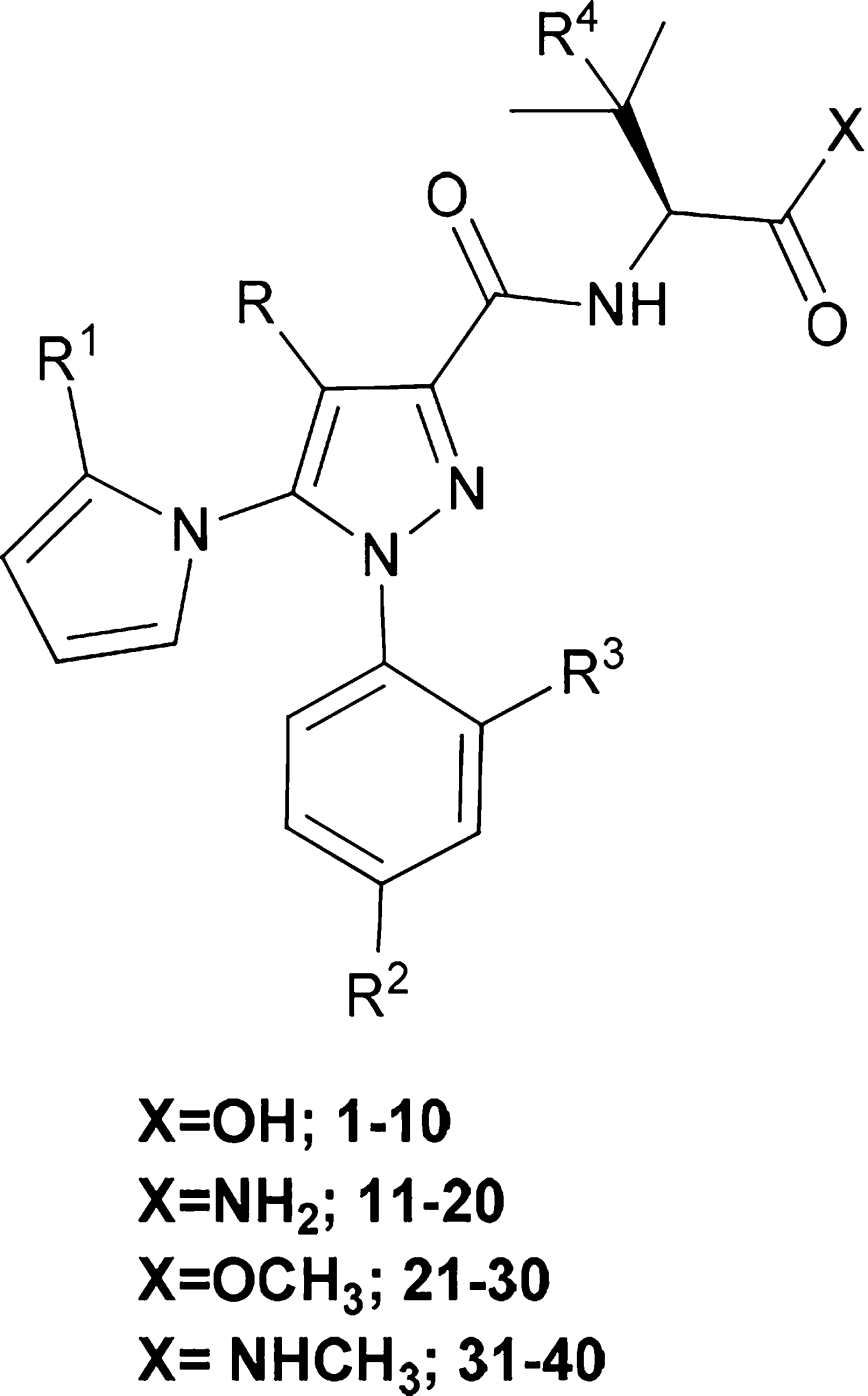
Sequences and Structures of Compounds **1–40**

**Compd**	**Sequence**	**R**	**R**^**1**^	**R**^**2**^	**R**^**3**^	**R**^**4**^		**Compd**	**Sequence**	**R**	**R**^**1**^	**R**^**2**^	**R**^**3**^	**R**^**4**^
**1**	**RS2689**-Val-OH	H	H	Cl	H	H		**21**	**RS2689**-Val-OCH_3_	H	H	Cl	H	H
**2**	**RS2689**-*t*Leu-OH	H	H	Cl	H	CH_3_		**22**	**RS2689**-*t*Leu-OCH_3_	H	H	Cl	H	CH_3_
**3**	**RS2691**-Val-OH	CH_3_	H	Cl	H	H		**23**	**RS2691**-Val-OCH_3_	CH_3_	H	Cl	H	H
**4**	**RS2691**-*t*Leu-OH	CH_3_	H	Cl	H	CH_3_		**24**	**RS2691**-*t*Leu-OCH_3_	CH_3_	H	Cl	H	CH_3_
**5**	**RS2708**-Val-OH	H	H	H	H	H		**25**	**RS2708**-Val-OCH_3_	H	H	H	H	H
**6**	**RS2708**-*t*Leu-OH	H	H	H	H	CH_3_		**26**	**RS2708**-*t*Leu-OCH_3_	H	H	H	H	CH_3_
**7**	**RS2709**-Val-OH	CH_3_	H	H	H	H		**27**	**RS2709**-Val-OCH_3_	CH_3_	H	H	H	H
**8**	**RS2709**-*t*Leu-OH	CH_3_	H	H	H	CH_3_		**28**	**RS2709**-*t*Leu-OCH_3_	CH_3_	H	H	H	CH_3_
**9**	**RS3516**-Val-OH	CH_3_	Cl	Cl	Cl	H		**29**	****RS3516****-Val-OCH_3_	CH_3_	Cl	Cl	Cl	H
**10**	**RS3516**-*t*Leu-OH	CH_3_	Cl	Cl	Cl	CH_3_		**30**	**RS3516**-*t*Leu-OCH_3_	CH_3_	Cl	Cl	Cl	CH_3_
**11**	**RS2689**-Val-NH_2_	H	H	Cl	H	H		**31**	**RS2689**-Val-NHCH_3_	H	H	Cl	H	H
**12**	**RS2689**-*t*Leu-NH_2_	H	H	Cl	H	CH_3_		**32**	**RS2689**-*t*Leu-NHCH_3_	H	H	Cl	H	CH_3_
**13**	**RS2691**-Val-NH_2_	CH_3_	H	Cl	H	H		**33**	**RS2691**-Val-NHCH_3_	CH_3_	H	Cl	H	H
**14**	**RS2691**-*t*Leu-NH_2_	CH_3_	H	Cl	H	CH_3_		**34**	**RS2691**-*t*Leu-NHCH_3_	CH_3_	H	Cl	H	CH_3_
**15**	**RS2708**-Val-NH_2_	H	H	H	H	H		**35**	**RS2708**-Val-NHCH_3_	H	H	H	H	H
**16**	**RS2708**-*t*Leu-NH_2_	H	H	H	H	CH_3_		**36**	**RS2708**-*t*Leu-NHCH_3_	H	H	H	H	CH_3_
**17**	**RS2709**-Val-NH_2_	CH_3_	H	H	H	H		**37**	**RS2709**-Val-NHCH_3_	CH_3_	H	H	H	H
**18**	**RS2709**-*t*Leu-NH_2_	CH_3_	H	H	H	CH_3_		**38**	**RS2709**-*t*Leu-NHCH_3_	CH_3_	H	H	H	CH_3_
**19**	**RS3516**-Val-NH_2_	CH_3_	Cl	Cl	Cl	H		**39**	**RS3516**-Val-NHCH_3_	CH_3_	Cl	Cl	Cl	H
**20**	**RS3516**-tLeu-NH_2_	CH_3_	Cl	Cl	Cl	CH_3_		**40**	**RS3516**-*t*Leu-NHCH_3_	CH_3_	Cl	Cl	Cl	CH_3_

Compounds **11**–**20** were prepared on Fmoc-RINK amide resin (loading coefficient: 0.74 mmol/g) treated with piperidine 20% in DMF and acylated with the Fmoc-protected amino acids in the presence of DIPEA, TBTU as coupling reagent, and HOBt in DMF at room temperature (SI, Scheme S2). After Fmoc-group removal, the amino acyl resin was *N-*acylated with a series of **RS** scaffolds, and then the amides were cleaved from the resin under acidic conditions. The series of methyl amide compounds **31**–**40** were synthesized in solution from the Boc-protected amino acid, which was converted into the respective *N*-methyl amide derivative (SI, Scheme S3). After Boc deprotection, the amino *N*-methyl amides were coupled to the **RS** scaffolds. Compounds **31**–**40** were obtained in excellent overall yields after purification on silica gel. All the final compounds **1**–**40** were fully characterized by low-resolution mass spectroscopy (LRMS) and proton nuclear magnetic resonance (^1^H NMR); the purities of the final products were evaluated by analytical RP-HPLC and were found to be ≥95% (details are included in the SI).

Our novel chemical compounds were tested *in vitro* for their binding ability, efficacy, and potency on CB1R in rat brain membrane homogenate (Table 2 and SI, Figure S1). CB1Rs are the most abundant G-protein coupled receptors in the rodent and mammalian brain; therefore, this is an appropriate model to characterize our ligands in a more physiological system.

**Table 2:** Binding Affinity (*K*_i_) and Signaling Properties of Compounds **1–40**^a^

		**[**^**35**^**S]GTPγS stimulation ± SEM**	
**Compd**	***K*_**i**_^3^H]WIN55,212-2 binding (nM) ± SEM**	***E*_max_ (%)**	**EC_50_ (nM)**
**WIN55,212-2**	11 ± 1	170 ± 1.7	143 ± 3.8
**rimonabant**	18 ± 2	38 ± 5	3086 ± 118
**1**	n.b.	63 ± 13	5874 ± 372
**2**	n.b.	74 ± 2.5	712 ± 18
**3**	n.b.	51 ± 5.6	>10 000
**4**	5879 ± 152	66 ± 10	4613 ± 303
**5**	n.b.	79 ± 4.9	1648 ± 75
**6**	n.b.	80 ± 7.3	2454 ± 118
**7**	n.b.	82 ± 18	2549 ± 106
**8**	n.b.	74 ± 14	3401 ± 118
**9**	n.b.	50 ± 15	3433 ± 283
**10**	n.b.	49 ± 10	1648 ± 98
**11**	341 ± 19	68 ± 9	1159 ± 79
**12**	102 ± 16	101 ± 1.8	n.r.
**13**	123 ± 13	97 ± 6	n.r.
**14**	21 ± 3.8	104 ± 1.4	n.r.
**15**	n.b.	53 ± 3.8	1233 ± 121
**16**	1470 ± 103	76 ± 4	295 ± 38
**17**	1059 ± 72	46 ± 12	4241 ± 377
**18**	60 ± 6.1	57 ± 13	3317 ± 242
**19**	446 ± 32	60 ± 2.9	849 ± 78
**20**	45 ± 4.4	70 ± 7	1875 ± 109
**21**	261 ± 27	52 ± 4.8	1362 ± 139
**22**	44 ± 4	43 ± 12	4960 ± 333
**23**	55 ± 5.2	45 ± 13	4869 ± 225
**24**	17 ± 3.7	99 ± 4	n.r.
**25**	1363 ± 16	54 ± 5	468 ± 22
**26**	283 ± 18	44 ± 5	1288 ± 151
**27**	449 ± 15	52 ± 7	2075 ± 129
**28**	369 ± 19	55 ± 5	1034 ± 117
**29**	30 ± 3.5	44 ± 10	2836 ± 118
**30**	21 ± 4.4	48 ± 8	4387 ± 344
**31**	387 ± 33	55 ± 5	601 ± 21
**32**	42 ± 2.9	120 ± 2	389 ± 51
**33**	168 ± 16	94 ± 2	n.r.
**34**	6.9 ± 1.8	135 ± 4	46 ± 4.1
**35**	7945 ± 266	76 ± 9	2458 ± 240
**36**	616 ± 16	98 ± 1.6	n.r.
**37**	1060 ± 85	55 ± 9	7075 ± 398
**38**	42 ± 3.9	102 ± 2	n.r.
**39**	185 ± 17	47 ± 8	3545 ± 193
**40**	n.b.	77 ± 4	237 ± 45

a n.b.: no binding of ligands was observed. n.r.: not relevant.

The binding and functional assays were performed using CB1 agonist WIN55,212-2 and inverse agonist rimonabant as reference ligands. Compounds **1**–**10** were not able to bind CB1Rs, while **11**–**20** bound to CB1Rs and stimulated GTP-binding; among them, **12**–**14** showed antagonist properties with binding affinity in the range of 21–123 nM. In the first case, it could be due to the ability of such compounds to modulate receptor function by binding at spatially distinct binding sites (the so-called allosteric ligands) or to exhibit off-target effects involving orphan GPCRs from adenosine, opioid or serotonin receptors, TRP channels, nuclear receptors, or ligand-gated ion channels.^18−20^ Some examples from the literature describe multitarget indazolyl ketones able to activate cannabinoid type 2 receptors (CB2Rs) while inhibiting cholinesterase and/or β-secretase enzymes; other ligands targeting PPAR-α and CB1Rs have been also designed fusing the pharmacophores of fibrates and the diarylpyrazole of rimonabant, giving compounds useful in the management of metabolic syndromes.^18^ The other compounds of this group exerted inverse agonist properties; in particular, **18** and **20** exhibited the strongest CB1 binding affinity (*K*_i_ = 60 and 18 nM, respectively). Both of them incorporate a *t*Leu residue and a methyl group in position 4, which are missing in the rimonabant scaffold. Even the CB1 antagonists **12**–**14** of the same series have at least one of the above substituents, suggesting their possible preference for acting as inverse agonists/antagonists. The valine and *tert-*leucine methyl ester derivatives **21**–**30** interacted with CB1Rs; among them, **22**, **23**, **29**, and **30** showed the highest binding affinities (*K*_i_ = 21–55 nM) to the CB1R. Compounds **22** and **30**, containing the *t*Leu residue, and **23** and **29**, containing the Val residue, conserve the *p*-Cl substitution of the rimonabant scaffolds. All the compounds of this series act as inverse agonists with micromolar EC_50_ values, with the only exception being the strong antagonist, **24**. This compound has the *t*Leu residue and a CH_3_ in position 4 of the pyrazole moiety; it also preserves the *p*-Cl phenyl group of rimonabant in position N1. Interestingly, the amides **12**–**14** and the methyl ester **24** are all CB1 antagonists, suggesting that the presence of a pyrrole in position 5 and the *p*-Cl substitution of the N1 phenyl ring could be key structural features in determining the specific G-protein-dependent activity regardless of their *C*-terminal functionalization. In the group of valine and *tert*-leucine methyl amide derivatives **31**–**40**, two agonist ligands, **32** and **34**, were found with strong potency and high CB1 binding affinity (*K*_i_ = 42 and 6.9 nM, respectively). It is worth noting that compound **34** possesses the same rimonabant-like scaffold of the compound 30 previously described by Silvestri et al.,^15^ with the only exception being a chlorine atom in position 3 on the aromatic ring. The presence of this substitution could be responsible for the slightly improved binding affinity of this compound (*K*_i_ = 5.6 nM) in comparison with the most active compound (**34**) of our series on the CB1R. Compounds **33**, **36**, and **38** acted as antagonists with *K*_i_ values of 42–600 nM; **31**, **35**, **37**, and **39** showed modest affinity toward CB1R and weak inverse agonist properties. Compounds **32** and **34** share a *t*Leu amino acid, a *p*-Cl phenyl group on N1 pyrazole, and a pyrrole on C5; otherwise, CB1 inverse agonists of this cluster present the Val residue, pyrrole, pyrazole, and phenyl groups with or without substitution on their rings. Compounds acting as CB1 antagonists (**33**, **36**, and **38**) did not show a significant preference for an amino acid residue and a phenyl substituent; however, all of them share an unsubstituted pyrrole moiety in common with **32** and **34** and amide/methyl ester derivatives described above. Overall, a clear trend can be delineated on the basis of these data: (*i*) the *C*-terminal methyl amide determines the CB1 agonist activity of *t*Leu-containing compounds preserving the *p*-Cl substitution on the phenyl ring and pyrrole moiety on C5 (e.g., **32** and **34**); (*ii*) the *N*-methyl amide function causes the shift of activity to CB1 antagonists (**12** and **14**) for the structures sharing the same rimonabant ring (e.g., **RS2689** and **RS2691**); and (*iii*) structures maintaining the same rimonabant scaffold switch their activity from agonist/inverse agonist to inverse agonist/antagonist depending on the amino acid residue (e.g., methyl ester derivatives **23**/**24**; methyl amide derivatives **34**/**33**, **32**/**31**).

Since compound **34** shows a significant binding affinity, which is the best of the series, we explored its ability to interact with CBR1 through the *in silico* method. After the ligand preparation and validation of docking protocol on the crystallographic ligand MDMB-fubinaca (PDB ID: 6N4B)^21^ (SI, Figure S2), a quantum mechanics/molecular mechanics (QM/MM) docking method was chosen by the Schrödinger QM-polarized ligand docking protocol (QPLD) for the novel agonist **34** following the parameters reported in the SI. The most favorable poses are depicted in Figure 2.

**Figure 2:**
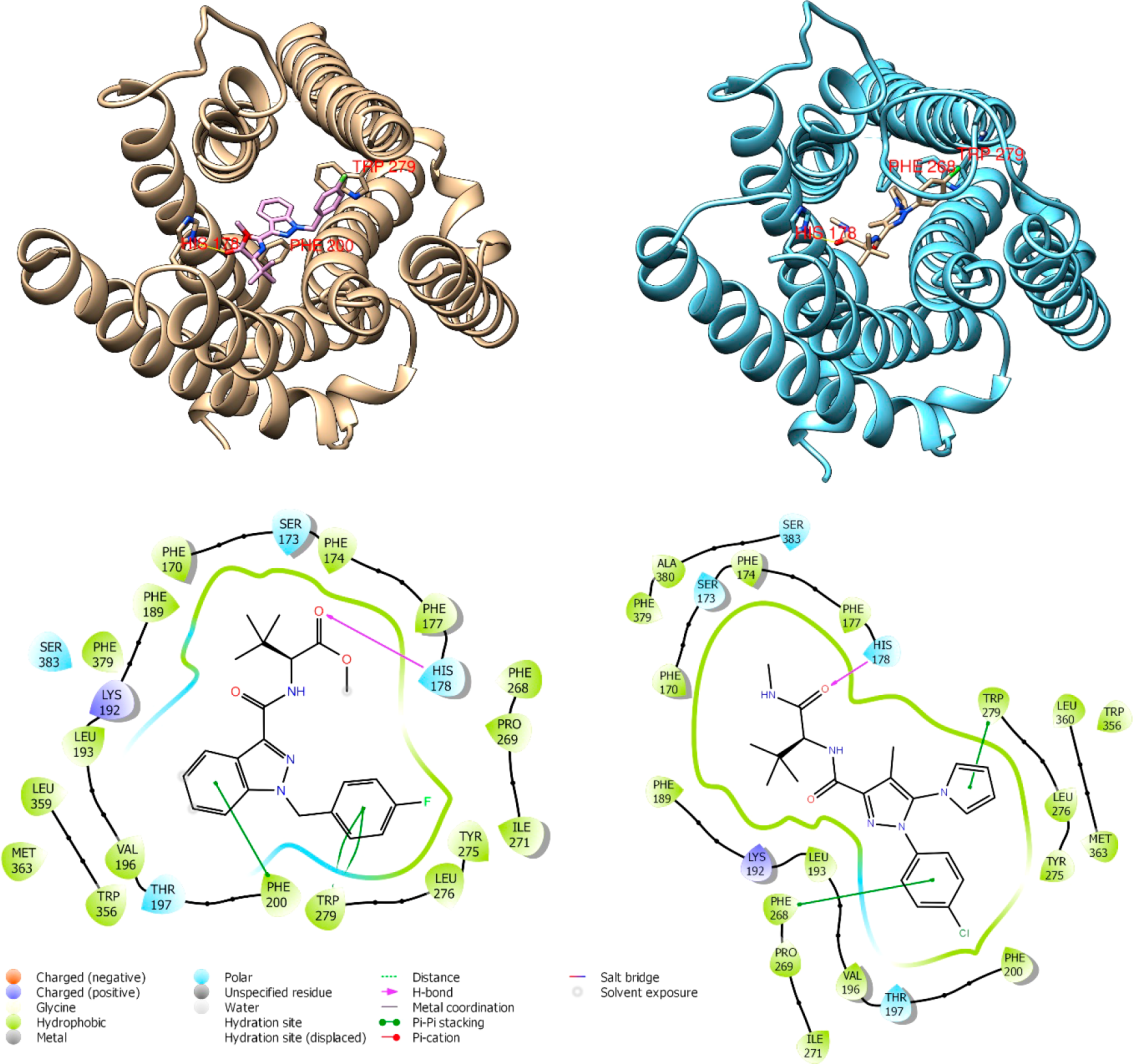
Best docking poses of crystallographic ligand MDMB-fubinaca (PDB 6N4B, left side) and **34** (right side) and their key interactions in the binding site.

The best docking pose of **34** presents two out of three of the interactions found in the crystallographic ligand–receptor complex, namely, π–π interactions toward Phe268 in place of Phe200, Trp279 and the hydrogen bond between the carbonyl group of the methyl amide *C*-terminal, and the His178 of the receptor binding site, in common with the interactions found for the receptor–ligand complex retrieved from the PDB. These data confirm the ability of such compounds to locate similar key structural elements inside the receptor pocket, retaining their strong binding affinity as CBR1 agonists. Furthermore, they help to understand the best binding affinity of compound **34** in comparison to the other *C*-terminal methyl amides; the presence of a *p*-chlorine electron-withdrawing atom on the aromatic moiety could be responsible for a well-balanced binding pocket interaction, which is favored by the correct orientation of the phenyl group. This perfectly fits the prominent hydrophobic task of the CB1R surrounded by Phe268, Trp279, Leu276, Phe200, Thr197, and Tyr275, thus furnishing an explanation for the strong binding affinity. Compounds characterized by more than one chlorine atom located on phenyl, pyrazole, and pyrrole rings possess *K*_i_ values higher than that of compound **34**; it is feasible to assume that the presence of diverse chlorine atoms exerts an unfavorable effect on the binding pocket’s interactions, confirming our hypothesis.

In light of the *in vitro* results, **14** was chosen as antagonist, **18**, **20**, **21**, **22**, **24**, **26**, and **29** as inverse agonists, and **34** as agonist of the CB1R to evaluate the *in vivo* food intake on mice, in comparison with rimonabant (Figure 3).

**Figure 3:**
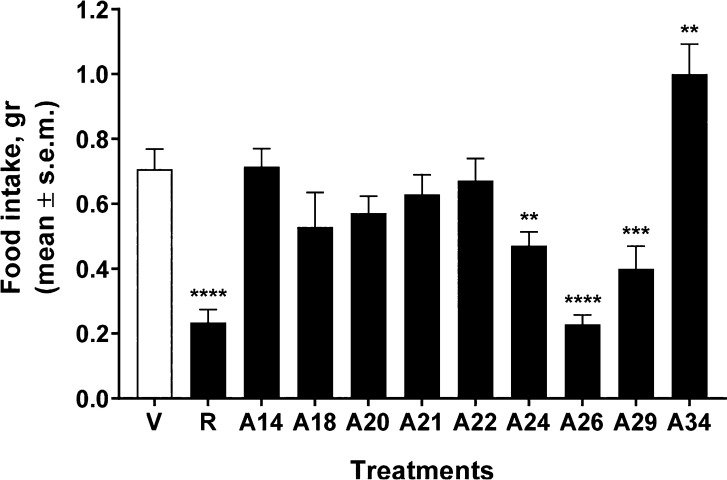
Activities of rimonabant (R), compounds **14**, **18**, **20**, **21**, **22**, **24**, **26**, **29**, and **34** (labeled as **A14–A34**), and vehicle (V, 1:1:18 mix of DMSO:Tween 80:sterile saline) on food intake. The tested compounds were injected i.p. (10 mg/kg), and after leaving for 30 min to allow the drug assimilation, mice were placed into individual cages with access to a predefined amount of their standard lab chow (2 g) for the 1-h test. The graphic bars represent the mean ± SEM of data from the same seven mice at each dose for food intake. **p < 0.01, ***p < 0.001, and ****p < 0.0001 vs V (vehicle-treated animals).

Compounds **14** and **22** showed an activity profile close to that of the vehicle, while **18**, **20**, **21**, and **24** exerted a slight reduction in food intake. Compounds **26** and **29** were able to induce a strong anorexic effect comparable to that of rimonabant, while compound **34** (*K*_i_ = 6.9 nM) was able to increase food intake in the mouse model. Compounds **24**, **26**, and **29** present diverse **RS** scaffolds and amino acid residues, while they share the same *C*-terminal derivatization. The result for compound **29** was not surprising since this compound has a biological profile *in vitro* very close to that of rimonabant (*K*_i_ = 30 nM versus 18 nM; *E*_max_ = 44% versus 38%; EC_50_ = 2836 nM versus 3086 nM for **29** and rimonabant, respectively). Otherwise, compound **34** exerts a strong orexant effect, probably due to its binding affinity for CB1R, which is higher than that of the standard agonist WIN55,212,2 (*K*_i_ = 6.9 nM versus 11 nM), as is its potency (EC_50_ = 46 nM versus 143 nM). It is well known that intraperitoneal (i.p.) administration of WIN55,212-2 used at doses of 1 mg/kg and 2 mg/kg is able to develop hyperphagia up to 6 h after the injection. It is worth noting that the administration of higher doses (5 mg/kg) was able to inhibit the food intake and motor behavior in partially satiated rats.^22^ Considering the importance of CB1 agonists in the management of chronic pain,^23^ we decided to investigate the anti-nociceptive profile of **34** in hot plate and formalin tests (Figure 4) in comparison with WIN55,212-2.

**Figure 4:**
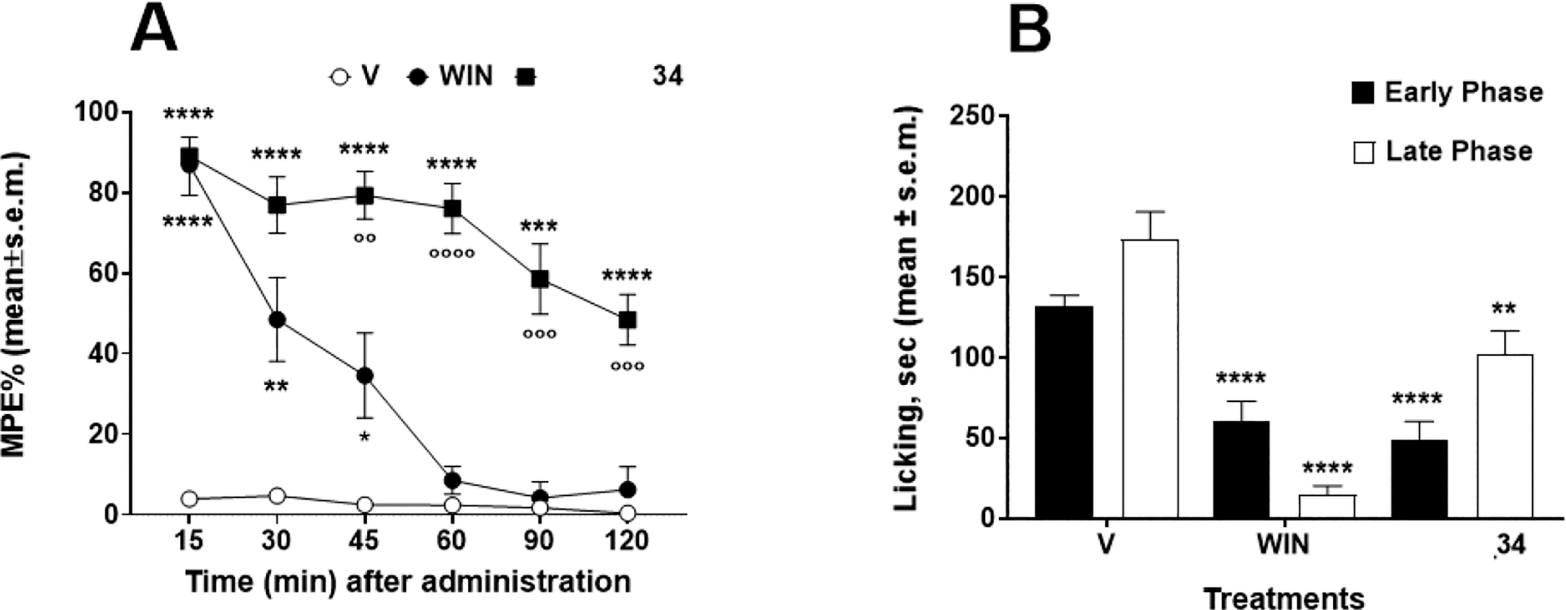
(A) Anti-nociceptive effect of WIN55,212-2 (WIN), **34**, and vehicle (V, saline 0.1% v/v DMSO) at the dose of 10 μg/10 μL after intracerebroventricular (i.c.v.) administration in the hot plate test. The anti-nociceptive activity is expressed as percentage of the maximum possible effect (%MPE ± SEM). *p < 0.05, **p < 0.01, ***p < 0.001, and ****p < 0.0001 vs V (vehicle-treated animals); °°*p* < 0.01, °°°*p* < 0.001, and °°°°*p* < 0.0001; *N* = 7. (B) Effects induced in the early phase (white bars) and in the late phase (black bars) of the formalin test by vehicle (V, DMSO:saline 1:3 (v/v)), **34**, and WIN55,212-2 (WIN). **34** and WIN were injected subcutaneously (s.c.) at a single dose (100 μg in 20 μL) 15 min before formalin injection (20 μL s.c.). Early: Licking activity recorded from 0 to 10 min after formalin administration. Late: Licking activity recorded from 15 to 40 min after formalin administration. The results obtained are expressed as the mean ± SEM; **p < 0.01 and ****p < 0.0001 vs V; *N* = 7.

In rats with spinal cord injury (SCI), repeated treatment with WIN55,212-2 provoked a sustained decrease in neuropathic pain-related behavior. On the contrary, the same treatment in uninjured rats led to a loss of anti-nociceptive efficacy, suggesting that the efficacy of this agonist may depend also on the initial pain state. Both models here described are acute pain state related tests.^24^ In the formalin test, **34** was slightly more effective than the pure CB1 agonist WIN55,212-2 in the early phase while it was less efficacious in the late phase, indicating a short duration of analgesic effect after subcutaneous (s.c.) administration. On the contrary, in the hot plate test, **34** demonstrated strong and long-lasting anti-nociceptive effect after intracerebroventricular (i.c.v.) injection since it significantly increased the response time of the animals to thermal nociceptive stimuli, between 15 and 120 min if compared to vehicle-treated animals. The effects of WIN55,212-2 diminished immediately after administration and reached an effect close to that observed for the animals treated with the saline solution 60 min later. Recent results from an experimental mice colitis model (dextran sulfate sodium salt (DSS)-induced colitis) reported WIN55,212-2 acting as an anti-inflammatory drug with improved pathological changes, including decreased levels of TNFα and IL6 in blood and myeloperoxidase (MPO) activity in colon.^25^ Zymosan-induced edema studies were performed to estimate the anti-inflammatory profile of **34** (Figure 5) after s.c. administration at 100 μg/20 μL of dose. WIN 55,212-2 and **34** induced an anti-inflammatory effect, but only **34** was able to significantly reduce paw volume increase induced by zymosan from 1 to 4 h after s.c. administration.

**Figure 5:**
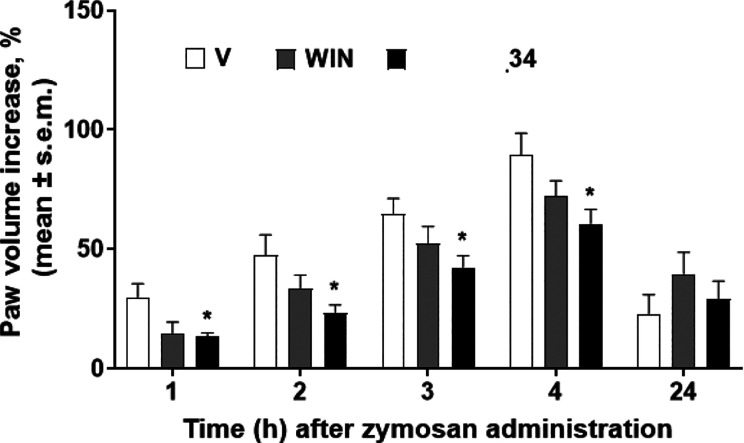
Effects of vehicle (V, saline containing 0.9% NaCl in the ratio DMSO:saline 1:3 (v/v)), WIN55,212-2 (WIN), and **34** on zymosan-induced paw edema. Zymosan (2.5% w/v in saline, 20 μL) was administered subcutaneously (s.c.) in the dorsal surface of the right hind paw. Drugs were administered s.c. in the dorsal surface of the right hind paw at the dose of 100 μg/20 μL, 15 min before zymosan. Paw volume was measured 1 h before zymosan and 1, 2, 3, 4, and 24 h thereafter. The paw volume increase was evaluated as the percentage between the paw volume at each time point and the basal paw volume. The results obtained are expressed as the mean ± SEM. *p < 0.05 vs V; *N* = 6.

The main issue with CB1R ligands is to avoid psychotropic effects due to penetration into the brain. In the first stage of our work we predicted the physicochemical properties of the best candidate, **34**, using the SwissADME tools^26^ (SI, Figure S3). The iLOG tool determined a moderate solubility in water, which pairs with the pharmacokinetic properties calculation.^27,28^ On the basis of these data, such a compound should not be able to penetrate the BBB, and it is not a good substrate for the permeability glycoprotein (P-gp protein). Overall, these results suggest the ability of **34** to exert its orexic effect as well as anti-inflammatory and anti-nociceptive activity on the periphery without the unwanted side effects of common CBR1 agonists. Chronic disorders are characterized by inflammatory states, and cannabinoids are reported to be beneficial in alleviating inflammation in several cases.^29−31^

In this work, we have explored the anti-inflammatory effect, food intake, anti-nociceptive activity *in vivo*, and the possible mechanism of action of rimonabant-related compounds bearing hydrophobic amino acid derivatives. *In vitro* biological assays revealed two agonists possessing a *t*Leu residue (e.g., **32** and **34**) in the *N*-methyl amide cluster. The presence of the *N*-methyl group in these compounds seems to be responsible for determining this activity since its removal induces the loss of agonist effect in favor of the antagonist one. This *in vitro* biological profile is strongly associated with the *in vivo* behavior, considering that **34** exerted a significant orexic effect after i.p. administration which closely resembles that of WIN55,212-2, a well-defined CB1 agonist. Compound **34** is able to produce an anti-nociceptive effect more potent than that of the reference compound by i.c.v. administration in the hot plate assay and by s.c. administration in the early phase of the formalin test. The results were also corroborated by the evaluation of its anti-inflammatory effect in the zymosan-induced edema formation model, where it reduced the paw volume increase from 1 to 4 h after s.c. administration. Works are underway to better characterize the pharmacological profile of **34***in vivo* as a leading drug for the management of anorexia and the complication related with chronic injuries and inflammatory pain states.

## Supporting Information Available

Experimental procedures, ^1^H NMR spectra, RP-HPLC analytical traces, *in silico* docking protocol, *in vitro* and *in vivo* biological assays procedures, and SwissADME predictions (PDF)

## Abbreviations

CB1R: cannabinoid type 1 receptor
CB2R: cannabinoid type 2 receptor
[^35^S]GTPγS: [^35^S]guanosine triphosphate, gamma phosphate group
ECS: endocannabinoid system
BBB: blood–brain barrier
CVD: cardiovascular disease
RP-HPLC: reverse-phase high-performance liquid chromatography
LRMS: low-resolution mass spectroscopy
Fmoc: fluorenylmethoxycarbonyl
Boc: *tert*-butyloxycarbonyl
DIPEA: *N*,*N*-diisopropylethylamine
TBTU: 2-(1*H*-benzotriazol-1-yl)-1,1,3,3-tetramethylaminium tetrafluoroborate
DMF: dimethylformamide
HOBt: hydroxybenzotriazole
^1^H NMR: proton nuclear magnetic resonance
*E*_MAX_: efficacy
EC_50_: potency
TRP: transient receptor potential
GPCR: G protein-coupled receptor
PPAR-α: peroxisome proliferator-activated receptor-α
QM/MM: quantum mechanics/molecular mechanics
QPLD: quantum mechanics-polarized ligand docking protocol
V: vehicle
i.p.: intraperitoneal
s.c.: subcutaneous
i.c.v.: intracerebroventricular
DMSO: dimethyl sulfoxide
SCI: spinal cord injury
DSS: dextran sulfate sodium salt
MPO: myeloperoxidase
TNFα: tumor necrosis factor-α
IL6: interleukin 6
ADME: absorption, distribution, metabolism, and excretion
P-gp: permeability glycoprotein
