# Supplementary material for: Rimonabant-Based Compounds Bearing Hydrophobic Amino Acid Derivatives as Cannabinoid Receptor Subtype 1 Ligands
Source: ACS Med Chem Lett. 2023 Mar 9;14(4):479–86. doi: 10.1021/acsmedchemlett.3c00024 (PMC10108392; doi:10.1021/acsmedchemlett.3c00024)
Supplement: Supplementary file 1 — ml3c00024_si_001.pdf [file ml3c00024_si_001.pdf]

SUPPORTING INFORMATION

Rimonabant based compounds bearing hydrophobic amino acid derivatives as cannabinoid receptor subtype 1 ligands

Szabolcs Dvorácskó,[1,2#] Marilisa Pia Dimmito,[3,#] Jessica Sebastiani,[4] Giuseppe La Regina,[4] Romano Silvestri,[4] Stefano Pieretti,[5] Azzurra Stefanucci,[3,*] Csaba Tömböly,[1,*] Adriano Mollica[3]

[1] Laboratory of Chemical Biology, Institute of Biochemistry, Biological Research Centre, Temesvári krt. 62., 6726, Szeged, Hungary.

[2] Department of Medicinal Chemistry, University of Szeged, H-6720 Szeged, Hungary.

[3] Department of Pharmacy, University "G. d'Annunzio" Chieti-Pescara, Via dei Vestini 31, 66100, Chieti, Italy.

[4] Laboratory Affiliated with the Institute Pasteur Italy - Cenci Bolognetti Foundation, Department of Drug Chemistry and Technologies, Sapienza University of Rome, Piazzale Aldo Moro 5, 00185 Rome, Italy.

[5] National Centre for Drug Research and Evaluation, Istituto Superiore di Sanità, Viale Regina Elena 299, 00161 Rome, Italy.

Co-first authors: Szabolcs Dvorácskó,[1,2#] Marilisa Pia Dimmito,[3,#]

Co-corresponding authors: Azzurra Stefanucci,[3,*] Csaba Tömböly [1,*]

e.mail: a.stefanucci@unich.it (A.S.); tomboly.csaba@brc.hu (C.T.)

TABLE OF CONTENTS	PAGES
Experimental procedures	S2-S16
In vitro biological assays	S17-S19
In vivo biological assays	S20,S21
In silico study	S22-S24
^{1}H-NMR spectra	S25-S64
RP-HPLC analytical traces	S65-S106
SwissADME prediction	S107

Experimental procedures

Methods and materials

Solvents, reagents, Boc- and Fmoc-protected amino acids were purchased from Sigma-Aldrich (Milano, Italy). The structures of the intermediates and the final compounds were characterized by ^{1}H-NMR spectra performed on a Varian Inova spectrometer (300 MHz, Varian Inc., Palo Alto, CA). Chemical shifts were reported in parts per million (δ) downfield from the internal standard tetramethylsilane (Me_4Si). The purity of each final product was established by analytical reverse phase-high performance liquid chromatography (RP-HPLC) (C18-bonded 4.6 × 150 mm) at a flow rate of 1 mL/min by using a gradient of H_2O/ACN 0.1% TFA ranging from 2% ACN to 98% ACN for 30 min. The purity of each compound was found to be >95%. Mass spectra were performed on an LCQ (Finnigan-Mat) ion trap mass spectrometer (San Jose, CA, USA) equipped with an electrospray ionization source. The capillary temperature was set at 300 °C and the spray voltage was set at 3.5 kV. The fluid was nebulized using nitrogen as both the sheath and the auxiliary gas. No unexpected or unusually high safety hazards were encountered.

Chemistry

General Procedure for the C-terminus acid Formation

Chlorotrityl chloride resin (100 mg, 1.60 mmol/g) was first swelled in DCM, the Fmoc protected amino-acid was dissolved in DCM with DIPEA and added to the vessel, swelling overnight at room temperature (r.t.). The resin was filtered and washed three times with DMF and DCM, then capping was performed with a mixture of 17 DCM/2 MeOH/1 DIPEA (7 mL x 3 x 5min). After filtration the resin was washed three times with DMF, MeOH and DCM, filtered and deprotected using a solution of 20% piperidine in DMF (2 x 15min). Kaiser test was used to check the completeness of each reaction step. A solution containing the desired Rimonabant scaffold (1.5 eq), HOBt (1.5 eq), TBTU (1.5 eq) DIPEA (3 eq) dissolved in DMF (3 mL) was added to the resin stirring until reaction completeness. The resin was washed and treated with a solution of 1%TFA/DCM (10 mL) for 1h, then the solution was collected and dried under vacuum.

General Procedure for the N-methyl Amide Formation

Boc-*tert*Leu-OH and Boc-Val-OH have been converted in their *N*-methyl amide derivatives using TBTU (1.1 eq), *N*-methylammonium·hydrochloride (3.3 eq) DIPEA (6 eq) in ACN (5 mL), stirred at room temperature overnight. After ACN removal, the crude product was extracted three times with citric acid solution (5% w/v), $NaHCO_3$ saturated solution (s.s.) and NaCl s.s. The

collected organic phases were dried and used for further reaction steps without purification (90% yield). The ^{1}H-NMR was used to confirm the intermediate structure. The Boc-protecting group has been removed using a solution of TFA/DCM (1:1) stirring for 1h at r.t. and the coupling reaction was performed using Rimonabant-scaffolds (1 eq), HOBt (1.1 eq), EDC·HCl (1.1 eq), DIPEA (2 eq) in DMF (3 mL). The reaction was stirred at r.t. for 12h. After DMF removal, the crude product was extracted three times with citric acid solution (5% w/v), $NaHCO_3$ s.s. and NaCl s.s. The collected organic phases were dried and purified on silica gel column (DCM:AcOEt = 9:1). The ^{1}H-NMR and LRMS techniques were used to confirm the structure.

General Procedure for the Methyl ester Formation

The methyl ester compounds have been prepared starting from the *C*-terminal acid compounds (1 eq) using EDC·HCl (1.1 eq), DMAP (0.3 eq) in anhydrous methanol (7 mL), stirring overnight. After DMF removal, the crude product was extracted three times with citric acid solution (5% w/v), $NaHCO_3$ s.s. and NaCl s.s. The collected organic phases were dried and purified using silica gel column (DCM:*n*-hexane = 7:3). The ^{1}H-NMR and LRMS techniques were used to confirm the desired structure.

General Procedure for the Amide Formation

Fmoc-Rink-amide resin (100 mg, 0.74 mmol/g) was swelled in DCM, the Fmoc protecting group was removed using a solution of 20% piperidine in DMF (2 x 15min). After checking the completeness of deprotection reaction using Kaiser test, the capping was performed using Ac_2O (2 eq.), DIPEA (2 eq.) in DMF (3 mL) for 30 min. The Fmoc-protected amino-acid was coupled using HOBt (1.5 eq), TBTU (1.5 eq), DIPEA (3 eq) dissolved in DMF (3 mL); the reaction mixture was added to the resin and stirred until reaction completeness. After filtration the resin was washed three times with DMF, MeOH and DCM, filtered and deprotected using a solution of 20% piperidine in DMF (2 x 15min). A solution containing the Rimonabant scaffolds (1.5 eq), HOBt (1.5 eq), TBTU (1.5 eq), DIPEA (3 eq) dissolved in DMF (3 mL) was added to the resin and stirred until reaction completeness. After washing, the resin was treated with a solution of TFA/TIS/water = 95/2.5/2.5 for 1h, then the solution was collected and dried under vacuum.

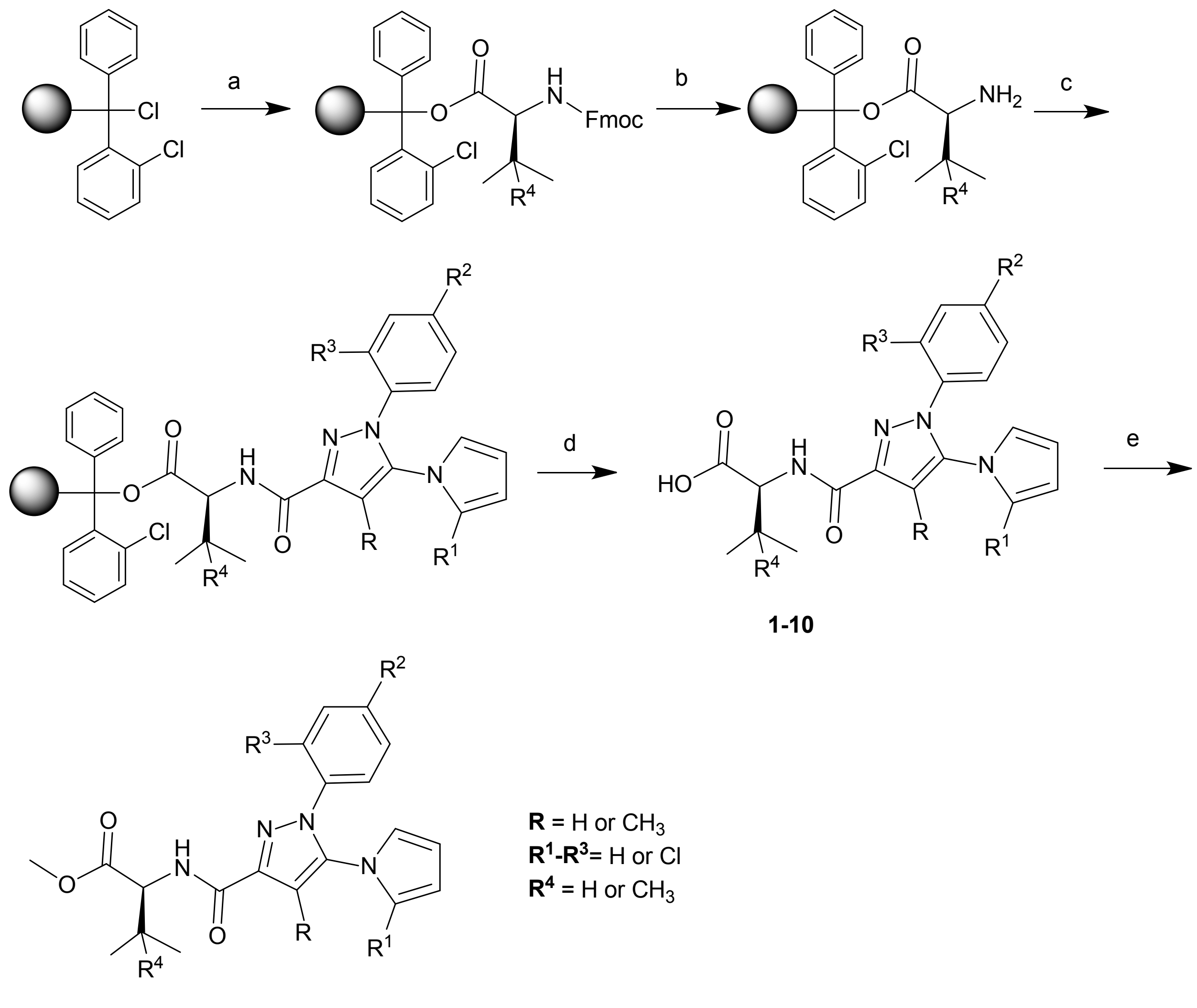

21-30

Scheme S1. Reagents and conditions: a) Fmoc-Val-OH or Fmoc-*t*Leu-OH, DIPEA, DCM, r.t., 12h; b) 20% piperidine/DMF, r.t., 15 min x 2; c) RS2689/RS2708/RS2709/RS2691/RS3516, HOBt, TBTU, DIPEA, DMF, r.t., 12h; d) 1% TFA/DCM, r.t., 1h; e) EDC·HCl, DMAP, MeOH anhydrous, r.t., 12h.

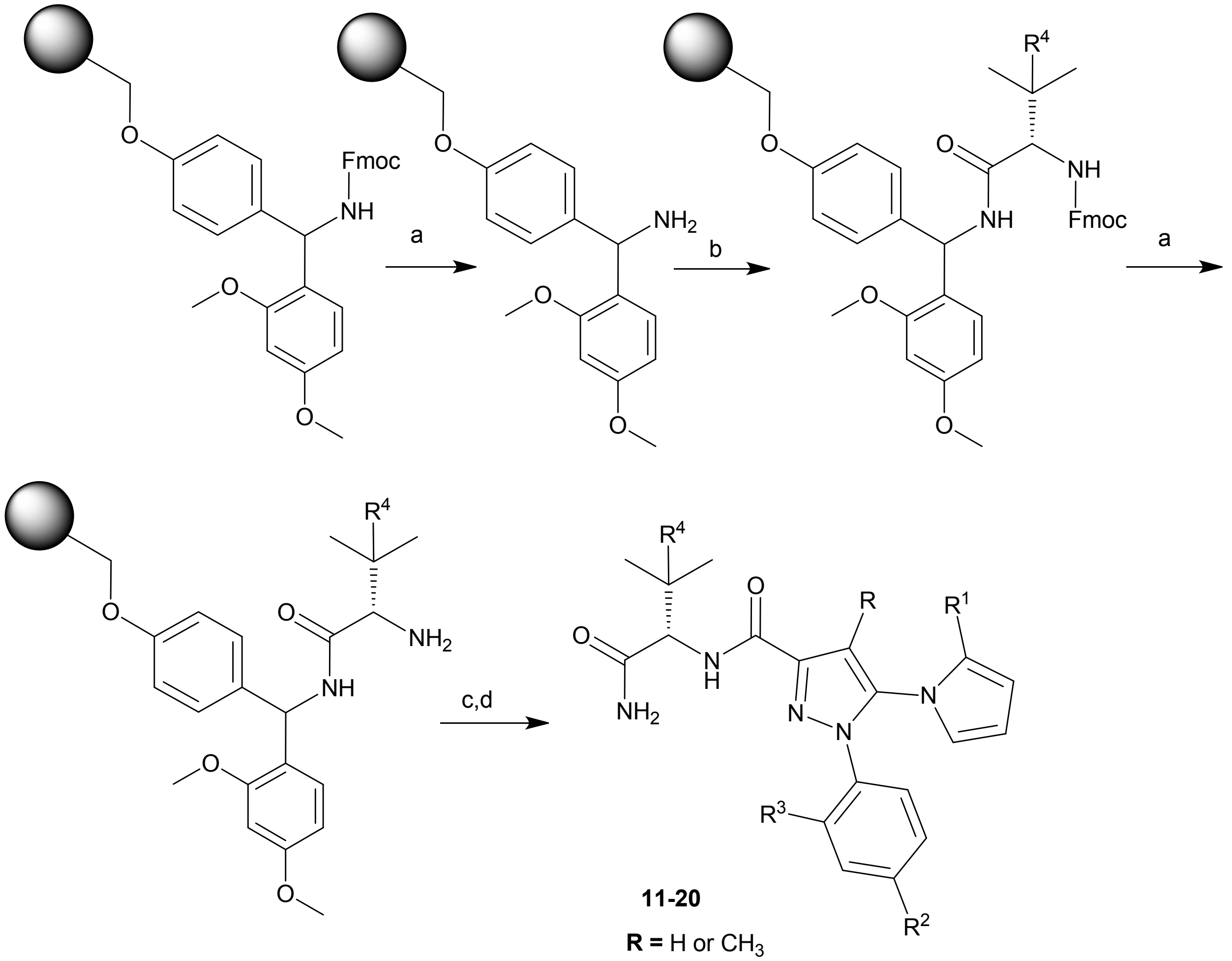

11-20

R = H or CH_3

R^1-R^3 = H or Cl

R^4 = H or CH_3

Scheme S2. Reagents and conditions: a) 20% Piperidine/DMF, r.t., 15 min x 2; b) Fmoc-Val-OH or Fmoc-*t*Leu-OH, HOBt, TBTU, DIPEA, DMF, r.t., 12 h; c) RS2689/RS2708/RS2709/RS2691/RS3516, HOBt, TBTU, DIPEA, DMF, r.t., 12h; d) 1% TFA/DCM, r.t., 1h.

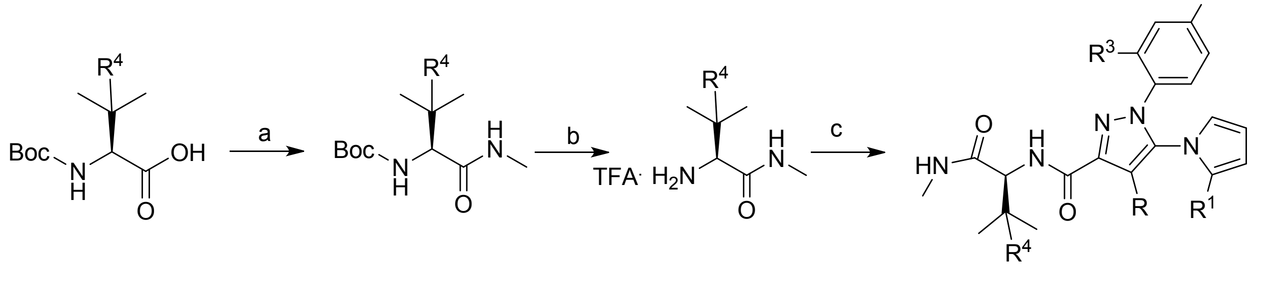

31-40

R = H or CH_3

R^1-R^3 = H or Cl

R^4 = H or CH_3

Scheme S3. Reagents and conditions: a) *N*-methyl ammonium hydrochloride, TBTU, DIPEA in ACN, r.t., 12h; b) TFA/DCM (1:1), r.t., 1h; c) RS2689/RS2708/RS2709/RS2691/RS3516, HOBt, EDC·HCl, DIPEA in DMF, r.t., 12h.

Compounds characterization

1: RS2689-Val-OH ((S)-2-(1-(4-chlorophenyl)-5-(1H-pyrrol-1-yl)-1H-pyrazole-3-carboxamido)-3-methylbutanoic acid) was obtained in 61 % yield, after reaction work up. R_t (analytical RP-HPLC) = 20.12 min. ^{1}H NMR (300 MHz, DMSO-d$_6$) δ 7.98 (d, *J* = 2.9 Hz, 1H, NH amide), 7.49 (d, *J* = 11.7 Hz, 2H, phenyl aromatics), 7.23 (d, *J* = 11.7 Hz, 2H, phenyl aromatics), 7.01 (s, 1H, pyrazole), 6.86 (m, 2H, pyrrole), 6.21 (m, 2H, pyrrole), 4.34 (t, 1H, CH$^{\alpha}$ Val), 2.21-2.16 (m, 1H, CH$^{\beta}$ Val), 0.94-0.91 (m, 6H, CH$_3$ x 2 Val). LRMS calcd. for $C_{19}H_{19}ClN_4O_3$: 386.1; found: 385.0 $[M-H]^-$.

2: RS2689-*t*Leu-OH (S)-2-(1-(4-chlorophenyl)-5-(1H-pyrrol-1-yl)-1H-pyrazole-3-carboxamido)-3,3-dimethylbutanoic acid) was obtained in 52% yield, after reaction work up. R_t (analytical RP-HPLC) = 20.36 min. ^{1}H NMR (300 MHz, DMSO-d$_6$) δ 10.52 (s, 1H, *C*-terminus acid), 7.64 (d, *J* = 3.1 Hz, 1H, NH amide), 7.49 (d, *J* = 11.7 Hz, 2H, phenyl aromatics), 7.22 (d, *J* = 11.7 Hz, 2H, phenyl), 7.03 (s, 1H, pyrazole), 6.86 (m, 2H, H pyrrole), 6.21 (m, 2H, pyrrole), 4.35 (d, *J* = 3.1 Hz, 1H, CH$^{\alpha}$ *t*Leu), 0.99 (s, 9H, CH$_3$ x 3 Leu) LRMS calcd. for $C_{20}H_{21}ClN_4O_3$: 400.1; found: 400.7 [M].

3: RS2691-Val-OH ((S)-2-(1-(4-chlorophenyl)-4-methyl-5-(1H-pyrrol-1-yl)-1H-pyrazole-3-carboxamido)-3-methylbutanoic acid) was obtained in 64% yield, after reaction work up. R_t (analytical RP-HPLC) = 21.01 min. ^{1}H NMR (300 MHz, DMSO-d$_6$) δ 7.89 (d, *J* = 2.9 Hz, 1H, NH amide), 7.47 (d, *J* = 11.2 Hz, 2H, phenyl aromatics), 7.17 (d, *J* = 11.2 Hz, 2H, phenyl aromatics), 6.88 (m, 2H, H pyrrole), 6.26 (m, 2H, pyrrole), 4.35 (t, 1H, CH$^{\alpha}$ Val), 2.20 (m, 1H, CH$^{\beta}$-Val), 2.08 (s, 3H, CH$_3$ pyrazole), 0.95-0.91 (m, 6H, CH$_3$ x 2 Val). LRMS calcd. for $C_{20}H_{21}ClN_4O_3$: 400.1; found: 401.1 $[M+H]^+$.

4: RS2691-*t*Leu-OH ((S)-2-(1-(4-chlorophenyl)-4-methyl-5-(1H-pyrrol-1-yl)-1H-pyrazole-3-carboxamido)-3,3-dimethylbutanoic acid) was obtained in 73% yield, after reaction work up. R_t (analytical RP-HPLC) = 21.10 min. ^{1}H NMR (300 MHz, DMSO-d$_6$) δ 7.62 (d, *J* = 3.1 Hz, 1H, NH amide), 7.46 (d, *J* = 11.2 Hz, 2H, phenyl aromatics), 7.16 (d, *J* = 11.2 Hz, 2H, phenyl aromatics), 6.87 (m, 2H, pyrrole), 6.25 (m, 2H, pyrrole), 4.34 (d, *J* = 3.1 Hz, 1H, CH$^{\alpha}$ *t*Leu), 2.09

(s, 3H, CH_3 pyrazole), 0.99 (s, 9H, CH_3 x 3 Leu). LRMS calcd. for $C_{21}H_{23}ClN_4O_3$: 414.1; found: 415.1 $[M+H]^+$

5: RS2708-Val-OH (S)-3-methyl-2-(1-phenyl-5-(1H-pyrrol-1-yl)-1H-pyrazole-3-carboxamido)butanoic acid) was obtained in 55% yield, after reaction work up. R_t (analytical RP-HPLC) = 19.32 min. ^1H NMR (300 MHz, DMSO-d_6) δ 7.94 (d, *J* = 2.9 Hz, 1H, NH amide), 7.42-7.40 (m, 3H, phenyl aromatics), 7.25-7.22 (m, 2H, phenyl aromatics), 6.99 (s, 1H, pyrazole), 6.83 (m, 2H, pyrrole), 6.18 (m, 2H, pyrrole), 4.34 (t, 1H, CH^{α} Val), 2.20 (m, 1H, CH^{β}-Val), 0.94-0.90 (m, 6H, CH_3 x 2 Val). LRMS calcd. for $C_{19}H_{20}N_4O_3$: 352.1; found: 353.0 $[M+H]^+$.

6: RS2708-*t*Leu-OH ((S)-3,3-dimethyl-2-(1-phenyl-5-(1H-pyrrol-1-yl)-1H-pyrazole-3-carboxamido) butanoic acid) was obtained in 51% yield, after reaction work up. R_t (analytical RP-HPLC) = 19.75 min. ^1H NMR (300 MHz, DMSO-d_6) δ 7.62 (d, *J* = 3.2 Hz, 1H, NH amide), 7.43-7.41 (m, 3H, phenyl aromatics), 7.25-7.22 (m, 2H, phenyl aromatics), 7.01 (s, 1H, pyrazole), 6.83 (m, 2H, pyrrole), 6.18 (m, 2H, pyrrole), 4.35 (d, *J* = 3.1 Hz, 1H, CH^{α} *t*Leu), 0.99 (s, 9H, CH_3 x 3 Leu). LRMS calcd. for $C_{20}H_{22}N_4O_3$: 366.2; found: 367.0 $[M+H]^+$.

7: RS2709-Val-OH ((S)-3-methyl-2-(4-methyl-1-phenyl-5-(1H-pyrrol-1-yl)-1H-pyrazole-3-carboxamido)butanoic acid) was obtained in 68% yield, after reaction work up. R_t (analytical RP-HPLC) = 19.81 min. ^1H NMR (300 MHz, DMSO-d_6) δ 7.84 (d, *J* = 2.9 Hz, 1H, NH amide), 7.40-7.34 (m, 3H, phenyl aromatics), 7.19-7.16 (m, 2H, phenyl aromatics), 6.86 (m, 2H, pyrrole), 6.23 (m, 2H, pyrrole), 4.35 (t, 1H, CH^{α} Val), 2.20 (m, 1H, CH^{β} Val), 2.08 (s, 3H, CH_3 pyrazole), 0.94-0.90 (m, 6H, CH_3 x 2 Val). LRMS calcd. for $C_{20}H_{22}N_4O_3$: 366.2; found: 367.0 $[M+H]^+$.

8: RS2709-*t*Leu-OH ((S)-3,3-dimethyl-2-(4-methyl-1-phenyl-5-(1H-pyrrol-1-yl)-1H-pyrazole-3-carboxamido)butanoic acid) was obtained in 57% yield, after reaction work up. R_t (analytical RP-HPLC) = 20.62 min. ^1H NMR (300 MHz, DMSO-d_6) δ 7.59 (d, *J* = 3.1 Hz, 1H, NH amide), 7.38-7.18 (m, 3H, phenyl aromatics), 7.18-7.15 (m, 2H, phenyl aromatics), 6.85 (m, 2H, pyrrole), 6.23 (m, 2H, pyrrole), 4.34 (d, *J* = 3.2 Hz, 1H, CH^{α} *t*Leu), 2.08 (s, 3H, CH_3 pyrazole), 0.99 (s, 9H, CH_3 x 3 Leu). LRMS calcd. for $C_{21}H_{24}N_4O_3$: 380.2; found: 403.1 $[M+Na]^+$.

9: RS3516-Val-OH (2S)-2-(5-(2-chloro-1H-pyrrol-1-yl)-1-(2,4-dichlorophenyl)-4-methyl-1H-pyrazole-3-carboxamido)-3-methylbutanoic acid) was obtained in 68% yield, after reaction work up. R_t (analytical RP-HPLC) = 21.54 min. ^1H NMR (300 MHz, DMSO-d_6) δ 7.87-7.83 (m, 2H,

NH amide + 1H aromatics), 7.69 (m, 1H, pyrrole), 7.63-7.54 (m, 2H, aromatics), 6.95-6.92 (m, 1H, pyrrole), 6.21-6.18 (m, 2H, pyrrole), 4.30 (t, 1H, CH^{α} Val), 2.19 (m, 1H, CH^{β} Val), 2.07 (s, 3H, CH_3 pyrazole), 0.93-0.88 (m, 6H, CH_3 x 2 Val). LRMS calcd. for $C_{20}H_{19}Cl_3N_4O_3$: 468.0; found: 491.0 $[M+Na]^+$.

10: RS3516-*t*Leu-OH ((2S)-2-(5-(2-chloro-1H-pyrrol-1-yl)-1-(2,4-dichlorophenyl)-4-methyl-1H-pyrazole-3-carboxamido)-3,3-dimethylbutanoic acid) was obtained in 52% yield, after reaction work up. R_t (analytical RP-HPLC) = 21.83 min. ^1H NMR (300 MHz, DMSO-d_6) δ 7.84 (d, 1H, NH amide), 7.67-7.51 (m, 3H, phenyl aromatics), 6.94 (m, 1H, pyrrole), 6.19 (m, 2H, pyrrole), 4.32 (d, 1H, CH^{α} *t*Leu), 2.07 (s, 3H, CH_3 pyrazole), 0.98 (s, 9H, CH_3 x 3 Leu). LRMS calcd. for $C_{21}H_{21}Cl_3N_4O_3$: 482.1; found: 482.9 [M].

11: RS2689-Val-NH_2 (S)-N-(1-amino-3-methyl-1-oxobutan-2-yl)-1-(4-chlorophenyl)-5-(1H-pyrrol-1-yl)-1H-pyrazole-3-carboxamide) was obtained in 78% yield, after reaction work up. R_t (analytical RP-HPLC) = 19.43 min. ^1H NMR (300 MHz, DMSO-d_6) δ 7.78 (d, J = 3.1 Hz, 1H, NH amide), 7.59 (s, 1H, NH_2 amide), 7.49 (d, J = 3 Hz, 2H, phenyl aromatics), 7.23-7.19 (m, 3H, 2H phenyl aromatics + 1H NH_2 amide), 6.98 (s, 1H, pyrazole), 6.85 (m, 2H, pyrrole), 6.20 (m, 2H, pyrrole), 4.33 (t, 1H, CH^{α} Val), 2.05 (m, 1H, CH^{β} Val), 0.96-0.84 (m, 6H, CH_3 x 2 Val). LRMS calcd. for $C_{19}H_{20}ClN_5O_2$: 385.1; found: 385.9 [M].

12: RS2689-*t*Leu-NH_2 (S)-2-(1-(4-chlorophenyl)-5-(1H-pyrrol-1-yl)-1H-pyrazole-3-carboxamido)-3,3-dimethylbutanoic acid) was obtained in 54% yield, after reaction work up. R_t (analytical RP-HPLC) = 19.75 min. ^1H NMR (300 MHz, DMSO-d_6) δ 7.67 (s, 1H, NH *C*-terminal amide), 7.57 (d, J = 2 Hz, 1H, NH amide), 7.50 (d, J = 6.9 Hz, 2H, phenyl aromatics), 7.24-7.19 (m, 3H, 2H phenyl aromatics + 1H NH *C*-terminal amide), 6.99 (s, 1H, pyrazole), 6.86 (m, 2H, pyrrole), 6.21 (m, 2H, pyrrole), 4.41 (d, J = 3.1 Hz, 1H, CH^{α} *t*Leu), 0.95 (s, 9H, CH_3 x 3 Leu) LRMS calcd. for $C_{20}H_{22}ClN_5O_2$: 399.1; found: 399.9 [M].

13: RS2691-Val-NH_2 ((S)-N-(1-amino-3-methyl-1-oxobutan-2-yl)-1-(4-chlorophenyl)-4-methyl-5-(1H-pyrrol-1-yl)-1H-pyrazole-3-carboxamide) was obtained in 76% yield, after reaction work up. R_t (analytical RP-HPLC) = 20.21 min. ^1H NMR (300 MHz, DMSO-d_6) δ 7.75 (d, J = 3.2 Hz, 1H, amide), 7.59 (s, 1H, *C*-terminal amide), 7.46 (d, J = 1.6 Hz, 2H, phenyl aromatics), 7.18-7.14 (m, 3H, phenyl aromatics + NH *C*-terminal amide), 6.88 (m, 2H, pyrrole), 6.25 (m, 2H, pyrrole), 4.33 (t, 1H, CH^{α} Val), 2.07 (s, 3H, CH_3 pyrazole), 0.92-0.85 (m, 6H, CH_3 x 2 Val). LRMS calcd. for $C_{20}H_{22}ClN_5O_2$: 399.1; found: 399.9 [M].

14: RS2691-*t*LeuNH$_2$ (N-(1-amino-3,3-dimethyl-1-oxobutan-2-yl)-1-(4-chlorophenyl)-4-methyl-5-(1H-pyrrol-1-yl)-1H-pyrazole-3-carboxamide) was obtained in 49% yield, after reaction work up. R$_t$ (analytical RP-HPLC) = 20.92 min. ^{1}H NMR (300 MHz, DMSO-d$_6$) δ 7.66 (s, 1H, NH$_2$ amide), 7.58 (d, *J* = 3.4 Hz, 1H, NH amide), 7.46 (d, *J* = 3 Hz, 2H, phenyl aromatics), 7.23 (s, 1H, NH$_2$ amide), 7.14 (d, *J* = 2.9 Hz, 2H, phenyl aromatics), 6.88 (m, 2H, H pyrrole), 6.25 (m, 2H, H pyrrole), 4.38 (d, *J* = 3.4 Hz, 1H, CH^α *t*Leu), 2.06 (s, 3H, CH$_3$ pyrazole), 0.99 (s, 9H, CH$_3$ x 3 *t*Leu). LRMS calcd. for $C_{21}H_{24}ClN_5O_2$: 413.2; found: 413.9 [M].

15: RS2708-Val-NH$_2$ ((S)-N-(1-amino-3-methyl-1-oxobutan-2-yl)-1-phenyl-5-(1H-pyrrol-1-yl)-1H-pyrazole-3-carboxamide) was obtained in 76% yield, after reaction work up. R$_t$ (analytical RP-HPLC) = 18.30 min. ^{1}H NMR (300 MHz, DMSO-d$_6$) δ 7.76 (d, *J* = 2.9 Hz, 1H, NH amide), 77.59 (s, 1H, NH$_2$ amide), 7.42-7.40 (m, 3H, phenyl aromatics), 7.24-7.19 (m, 2H, 2H phenyl aromatics + 1H NH$_2$ amide), 6.97 (s, 1H, pyrazole), 6.83 (m, 2H, pyrrole), 6.18 (m, 2H, pyrrole), 4.34 (t, 1H, CH^α Val), 2.05 (m, 1H, CH^β Val), 0.91-0.84 (dd, 6H, CH$_3$ x 2 Val). LRMS calcd. for $C_{19}H_{21}N_5O_2$: 351.2; found: 352.0 $[M+H]^+$.

16: RS2708-*t*Leu-NH$_2$ (S)-N-(1-amino-3,3-dimethyl-1-oxobutan-2-yl)-1-phenyl-5-(1H-pyrrol-1-yl)-1H-pyrazole-3-carboxamide) was obtained in 43% yield, after reaction work up. R$_t$ (analytical RP-HPLC) = 19.28 min. ^{1}H NMR (300 MHz, DMSO-d$_6$) δ 7.67 (s, 1H, *C*-terminal amide), 7.56 (d, *J* = 3.1 Hz, 1H, NH amide), 7.43-7.41 (m, 3H, phenyl aromatics), 7.24-7.21 (m, 3H, phenyl aromatics + NH *C*-terminal amide under aromatic signals), 6.97 (s, 1H, pyrazole), 6.83 (m, 2H, pyrrole), 6.18 (m, 2H, pyrrole), 4.40 (d, *J* = 3.1 Hz, 1H, CH^α *t*Leu), 0.99 (s, 9H, CH$_3$ x 3 Leu). LRMS calcd. for $C_{20}H_{23}N_5O_2$: 365.2; found: 366.0 $[M+H]^+$.

17: RS2709-Val-NH$_2$ (S)-N-(1-amino-3-methyl-1-oxobutan-2-yl)-4-methyl-1-phenyl-5-(1H-pyrrol-1-yl)-1H-pyrazole-3-carboxamide) was obtained in 78% yield, after reaction work up. R$_t$ (analytical RP-HPLC) = 19.80 min. ^{1}H NMR (300 MHz, DMSO-d$_6$) δ 7.72 (d, *J* = 2.9 Hz, 1H, NH amide), 7.59 (s, 1H, NH$_2$ amide), 7.38-7.35 (m, 3H, phenyl aromatics), 7.18-7.15 (m, 3H, 2H phenyl aromatics + 1H NH$_2$ amide), 6.85 (m, 2H, pyrrole), 6.22 (m, 2H, pyrrole), 4.33 (t, 1H, CH^α Val), 2.09-2.05 (m, 4H, CH^β Val + CH$_3$ pyrazole), 0.98-0.85 (m, 6H, CH$_3$ x 2 Val). LRMS calcd. for $C_{20}H_{23}N_5O_2$: 365.2; found: 365.9 [M].

18: RS2709-*t*Leu-NH$_2$ ((S)-N-(1-amino-3,3-dimethyl-1-oxobutan-2-yl)-4-methyl-1-phenyl-5-(1H-pyrrol-1-yl)-1H-pyrazole-3-carboxamide). The desired compound was obtained in 56% yield, after reaction work up. R$_t$ (analytical RP-HPLC) = 19.63 min. ^{1}H NMR (300 MHz,

DMSO-d_6) δ 7.66 (s, 1H, *C*-terminal amide), 7.58 (d, *J* = 3.1 Hz, 1H, NH amide), 7.38-7.36 (m, 3H, phenyl aromatics), 7.22 (s, 1H, *C*-terminal amide), 7.17-7.14 (m, 2H, phenyl aromatics), 6.86 (m, 2H, pyrrole), 6.22 (m, 2H, pyrrole), 4.38 (d, *J* = 3.1 Hz, 1H, CH^{α} *t*Leu), 2.10 (s, 3H, CH_3 pyrazole), 0.96 (s, 9H, CH_3 x 3 Leu). LRMS calcd. for $C_{21}H_{25}N_5O_2$: 379.2; found: 781.0 $[2M+Na]^+$.

19: RS3516-Val-NH_2 (N-((S)-1-amino-3-methyl-1-oxobutan-2-yl)-5-(2-chloro-1H-pyrrol-1-yl)-1-(2,4-dichlorophenyl)-4-methyl-1H-pyrazole-3-carboxamide) was obtained in 88% yield, after reaction work up. R_t (analytical RP-HPLC) = 24.34 min. ^1H NMR (300 MHz, DMSO-d_6) δ 7.85 (d, 1H, NH amide), 7.73-7.54 (m, 5H, 3H aromatics + NH_2 *C*-terminal amide under aromatic signals), 7.22 (d, 1H, pyrrole), 6.95 (m, 1H, pyrrole), 6.20 (m, 1H, pyrrole), 4.32 (t, 1H, CH^{α} Val), 2.07 (s, 4H, CH_3 pyrazole + CH^{β} Val), 0.98-0.90 (dd, 6H, CH_3 x 2 Val). LRMS calcd. for $C_{20}H_{20}Cl_3N_5O_2$: 467.1; found: 468.7 $[M+H]^+$.

20: RS3516-*t*Leu-NH_2 (N-((S)-1-amino-3,3-dimethyl-1-oxobutan-2-yl)-5-(2-chloro-1H-pyrrol-1-yl)-1-(2,4-dichlorophenyl)-4-methyl-1H-pyrazole-3-carboxamide). The desired compound was obtained in 63% yield, after reaction work up. R_t (analytical RP-HPLC) = 23.84 min. ^1H NMR (300 MHz, DMSO-d_6) δ 7.85 (s, 1H, NH amide), 7.70-7.50 (m, 3H, aromatics), 7.28 (s, 1H, *C*-terminal amide), 6.95 (s, 1H, *C*-terminal amide), 6.19 (m, 2H, pyrrole), 5.74 (m, 1H, pyrrole), 4.38 (d, 1H, CH^{α} *t*Leu), 2.08 (s, 3H, CH_3 pyrazole), 0.94 (s, 9H, CH_3 x 3 *t*Bu). LRMS calcd. for $C_{21}H_{22}Cl_3N_5O_2$: 481.1; found: 504.0 $[M+Na]^+$.

21: RS2689-Val-OCH_3 ((S)-methyl 2-(1-(4-chlorophenyl)-5-(1H-pyrrol-1-yl)-1H-pyrazole-3-carboxamido)-3-methylbutanoate). The desired compound was obtained in 89% yield, after reaction work up. R_t (analytical RP-HPLC) = 21.50 min. ^1H NMR (300 MHz, DMSO-d_6) δ 8.31 (d, *J* = 2.7 Hz, 1H, NH amide), 7.50 (d, *J* = 3 Hz, 2H, phenyl aromatics), 7.23 (d, *J* = 3Hz, 2H, phenyl aromatics), 7.01 (s, 1H, pyrazole), 6.86 (s, 2H, pyrrole), 6.21 (s, 2H, pyrrole), 4.34 (t, 1H, CH^{α} Val), 3.65 (s, 3H, OCH_3), 2.19 (m, 1H, CH^{β} Valine), 0.92 (dd, 6H, CH_3 x 2 Valine). LRMS calcd. for $C_{20}H_{21}ClN_4O_3$: 400.1; found: 401.1 $[M+H]^+$.

22: RS2689-*t*Leu-OCH_3 (S)-methyl 2-(1-(4-chlorophenyl)-5-(1H-pyrrol-1-yl)-1H-pyrazole-3-carboxamido)-3,3-dimethylbutanoate). The desired compound was obtained in 55% yield, after reaction work up. R_t (analytical RP-HPLC) = 24.96 min. ^1H NMR (300 MHz, DMSO-d_6) δ 7.9 (d, *J* = 2.7 Hz, 1H, NH amide), 7.49 (d, 2H, aromatics), 7.22 (d, 2H, aromatics), 7.05 (s, 1H, H pyrazole), 6.86 (m, 2H, H pyrrole), 6.22 (m, 2H, H pyrrole), 4.43 (d, 1H, CH^{α} *t*Leu), 3.77 (s, 3H,

-OCH_3), 0.99 (s, 9H, CH_3 x 3 *t*Bu). LRMS calcd. for $C_{21}H_{23}ClN_4O_3$: 414.1; found: 437.1 $[M+Na]^+$

23: RS2691-Val-OCH_3 ((S)-methyl 2-(1-(4-chlorophenyl)-4-methyl-5-(1H-pyrrol-1-yl)-1H-pyrazole-3-carboxamido)-3-methylbutanoate) was obtained in 88% yield, after reaction work up. R_t (analytical RP-HPLC) = 22.01 min. ^1H NMR (300 MHz, DMSO-d_6) δ 8.18 (d, 1H, NH amide), 7.45 (d, 2H, aromatics), 7.16 (d, 2H, aromatics), 6.88 (m, 2H, H pyrrole), 6.26 (m, 2H, pyrrole), 4.34 (t, 1H, CH^{α} Val), 3.65 (s, 3H, -OCH_3), 2.20 (m, 1H, CH^{β} Val), 2.07 (s, 3H, CH_3 pyrazole), 0.91 (dd, 6H, CH_3 x 2 Val). LRMS calcd. for $C_{21}H_{23}ClN_4O_3$: 414.1; found: 415.0 $[M+H]^+$.

24: RS2691-*t*Leu-OCH_3 ((S)-methyl 2-(1-(4-chlorophenyl)-4-methyl-5-(1H-pyrrol-1-yl)-1H-pyrazole-3-carboxamido)-3,3-dimethylbutanoate) was obtained in 54% yield, after reaction work up. R_t (analytical RP-HPLC) = 26.07 min. ^1H NMR (300 MHz, DMSO-d_6) δ 7.78 (d, 1H, NH amide), 7.46 (d, 2H, aromatics), 7.17 (d, 2H, aromatics), 6.88 (m, 2H, pyrrole), 6.26 (m, 2H, pyrrole), 4.42 (d, 1H, CH^{α} *t*Leu), 3.67 (s, 3H, -OCH_3), 2.07 (s, 3H, CH_3 pyrazole), 0.99 (s, 9H, CH_3 x 3 *t*Leu). LRMS calcd. for $C_{22}H_{25}ClN_4O_3$: 428.2; found: 451.1$[M+Na]^+$.

25: RS2708-Val-OCH_3 ((S)-methyl 3-methyl-2-(1-phenyl-5-(1H-pyrrol-1-yl)-1H-pyrazole-3-carboxamido)butanoate) was obtained in 98% yield, after reaction work up. R_t (analytical RP-HPLC) = 22.89 min. ^1H NMR (300 MHz, DMSO-d_6) δ 8.31 (d, 1H, NH amide), 7.43-7.40 (m, 3H, phenyl aromatics), 7.26-7.22 (m, 2H, phenyl aromatics), 7.00 (s, 1H, pyrazole), 6.85 (m, 2H, H pyrrole), 6.18 (m, 2H, pyrrole), 4.34 (t, 1H, CH^{α} Val), 3.65 (s, 1H, -OCH_3), 2.21 (m, 1H, CH^{β} Val), 0.92 (dd, 6H, CH_3 x 2 Val). LRMS calcd. for $C_{20}H_{22}N_4O_3$: 366.2; found: 367.1 $[M+H]^+$.

26: RS2708-*t*Leu-OCH_3 (((S)-methyl 3,3-dimethyl-2-(1-phenyl-5-(1H-pyrrol-1-yl)-1H-pyrazole-3-carboxamido)butanoate) was obtained in 61% yield, after reaction work up. R_t (analytical RP-HPLC) = 23.68 min. ^1H NMR (300 MHz, DMSO-d_6) δ 7.79 (d, 1H, NH amide), 7.43-7.41 (m, 3H, phenyl aromatics), 7.25-7.23 (m, 2H, phenyl aromatics), 7.03 (s, 1H, H pyrazole), 6.84 (m, 2H, pyrrole), 6.19 (m, 2H, pyrrole), 4.44 (d, 1H, CH^{α} *t*Leu), 3.67 (s, 3H, -OCH_3), 0.99 (s, 9H, CH_3 x 3 *t*Leu). LRMS calcd. for $C_{21}H_{24}N_4O_3$: 380.2; found: 403.2 $[M+Na]^+$.

27: RS2709-Val-OCH_3 ((S)-methyl 3-methyl-2-(4-methyl-1-phenyl-5-(1H-pyrrol-1-yl)-1H-pyrazole-3-carboxamido)butanoate) was obtained in 64% yield, after reaction work up. R_t (analytical RP-HPLC) = 24.20 min. ^1H NMR (300 MHz, DMSO-d_6) δ: 8.15 (d, 1H, NH amide), 7.40-7.33 (m, 3H, phenyl aromatics), 7.20-7.17 (m, 2H, phenyl aromatics), 6.86 (m, 2H, H

pyrrole), 6.23 (m, 2H, H pyrrole), 4.36 (t, 1H, CH^{α} Val), 3.65 (s, 3H, $-OCH_3$), 2.22 (m, 1H, CH^{β} Val), 2.07 (s, 3H, CH_3 pyrazole), 0.91 (dd, 6H, CH_3 x 2 Val). LRMS calcd. for $C_{21}H_{24}N_4O_3$: 380.2; found: 402.2 $[M+Na]^+$.

28: RS2709-*t*Leu-OCH_3 (S)-methyl 3,3-dimethyl-2-(4-methyl-1-phenyl-5-(1H-pyrrol-1-yl)-1H-pyrazole-3-carboxamido)butanoate). The desired compound was obtained in 65% yield, after reaction work up. R_t (analytical RP-HPLC) = 24.78 min. ^{1}H NMR (300 MHz, DMSO-d_6) δ 7.74 (d, 1H, NH amide), 7.39-7.35 (m, 3H, phenyl aromatics), 7.19-7.16 (m, 2H, phenyl aromatics), 6.86 (m, 2H, H pyrrole), 6.24 (m, 2H, H pyrrole), 4.42 (d, 1H, CH^{α} *t*Leu), 3.67 (s, 3H, $-OCH_3$), 2.08 (s, 3H, CH_3 pyrazole), 0.99 (s, 9H, CH_3 x 3 Leu). LRMS calcd. for $C_{22}H_{26}N_4O_3$: 394.2; 417.2 $[M+Na]^+$.

29: RS3516-Val-OCH_3 ((2S)-methyl 2-(5-(2-chloro-1H-pyrrol-1-yl)-1-(2,4-dichlorophenyl)-4-methyl-1H-pyrazole-3-carboxamido)-3-methylbutanoate). The desired compound was obtained in 78% yield, after reaction work up. R_t (analytical RP-HPLC) = 22.54 min. ^{1}H NMR (300 MHz, DMSO-d_6) δ: 8.21 (d, 1H, NH amide), 7.84 (d, 1H, pyrrole), 7.69-7.54 (m, 2H, aromatics), 6.93 (m, 1H, aromatics), 6.18 (m, 2H, pyrrole), 4.27 (t, 1H, CH^{α} Val), 3.64 (s, 3H, $-OCH_3$), 2.21 (m, 1H, CH^{β} Val), 2.06 (s, 3H, CH_3, pyrazole), 0.88 (dd, 6H, CH_3 x 2 Val). LRMS calcd. for $C_{21}H_{21}Cl_3N_4O_3$: 482.1; found: 505.0 $[M+Na]^+$.

30: RS3516-*t*Leu-OCH_3 ((2S)-methyl 2-(5-(2-chloro-1H-pyrrol-1-yl)-1-(2,4-dichlorophenyl)-4-methyl-1H-pyrazole-3-carboxamido)-3,3-dimethylbutanoate). The desired compound was obtained in 54% yield, after reaction work up. R_t (analytical RP-HPLC) = 26.35 min. ^{1}H NMR (300 MHz, DMSO-d_6) δ 7.86 (d, 1H, NH amide), 7.71-7.54 (m, 3H phenyl aromatics), 6.97 (m, 1H, pyrrole), 6.20 (m, 2H, pyrrole), 4.40 (m, 1H, CH^{α} *t*Leu), 3.67 (s, 3H, $-OCH_3$), 2.07 (s, 3H, CH_3 pyrazole), 0.98 (s, 9H, CH_3 x 3 *t*Bu). LRMS calcd. for $C_{22}H_{23}Cl_3N_4O_3$: 496.1; found: 496.9 [M].

31: RS2689-Val-$NHCH_3$ (S)-1-(4-chlorophenyl)-N-(3-methyl-1-(methylamino)-1-oxobutan-2-yl)-5-(1H-pyrrol-1-yl)-1H-pyrazole-3-carboxamide. The desired compound was obtained in 85% yield, after reaction work up. R_t (analytical RP-HPLC) = 21.98 min. ^{1}H NMR (300 MHz, DMSO-d_6) δ 8.11 (q, 1H, NH-methylamide), 7.86 (d, 1H, NH amide), 7.50 (d, 2H, phenyl romatics), 7.22 (d, 2H, phenyl aromatics), 6.99 (s, 1H, H pyrazole), 6.86 (m, 2H, pyrrole), 6.21 (m, 2H, H pyrrole), 4.29 (t, 1H, CH^{α} Val), 2.59 (d, 3H, CH_3 methyl-amide), 2.06 (m, 1H, CH^{β} Val), 0.85 (dd, 6H, CH_3 x 2 Val). LRMS calcd. for $C_{20}H_{22}ClN_5O_2$: 399.1; found: 422.9 $[M+Na]^+$.

32: RS2689-*t*Leu-NHCH$_3$ 1-(4-chlorophenyl)-N-(3,3-dimethyl-1-(methylamino)-1-oxobutan-2-yl)-5-(1H-pyrrol-1-yl)-1H-pyrazole-3-carboxamide. The desired compound was obtained in 97% yield, after reaction work up. R$_t$ (analytical RP-HPLC) = 23.07 min. ^{1}H NMR (300 MHz, DMSO-d$_6$) δ 8.20 (q, 1H, NH-methyl amide), 7.57 (d, 1H, NH amide), 7.50 (d, 2H, phenyl aromatics), 7.22 (d, 2H, phenyl aromatics), 7.01 (s, 1H, pyrazole), 6.84 (m, 2H, pyrrole), 6.20 (m, 2H, pyrrole), 4.49 (d, 1H, CH^α *t*Leu), 2.60 (d, 3H, CH$_3$ methyl-amide), 0.99 (s, 9H, CH$_3$ x 3 *t*Bu). LRMS calcd. for $C_{21}H_{24}ClN_5O_2$: 413.2; found: 436.1 [M+Na]$^+$.
33: RS2691-Val-NHCH$_3$ (S)-1-(4-chlorophenyl)-4-methyl-N-(3-methyl-1-(methylamino)-1-oxobutan-2-yl)-5-(1H-pyrrol-1-yl)-1H-pyrazole-3-carboxamide) was obtained in 76% yield, after reaction work up. R$_t$ (analytical RP-HPLC) = 23.09 min. ^{1}H NMR (300 MHz, DMSO-d$_6$) δ 8.11 (q, 1H, NH methyl amide), 7.81 (d, 1H, NH amide), 7.47 (d, 2H, phenyl aromatics), 7.16 (d, 2H, phenyl aromatics), 6.88 (m, 2H, H pyrrole), 6.26 (m, 2H, H pyrrole), 4.30 (t, 1H, CH^α Val), 2.59 (d, 3H, CH$_3$ pyrazole), 2.08-1.96 (m, 4H, CH$_3$ methyl amide + 1H CH^β Val), 0.887 (dd, 6H, CH$_3$ x 2 Val). LRMS calcd. for $C_{21}H_{24}ClN_5O_2$: 413.1; found: 436.2 [M+Na]$^+$.
34: RS2691-*t*Leu-NHCH$_3$ (S)-1-(4-chlorophenyl)-N-(3,3-dimethyl-1-(methylamino)-1-oxobutan-2-yl)-4-methyl-5-(1H-pyrrol-1-yl)-1H-pyrazole-3-carboxamide) was obtained in 64% yield, after reaction work up. R$_t$ (analytical RP-HPLC) = 24.09 min. ^{1}H NMR (300 MHz, DMSO-d$_6$) δ 8.19 (q, 1H, NH methyl amide), 7.58 (d, 1H, NH amide), 7.48 (d, 2H, phenyl aromatics), 7.15 (d, 2H, phenyl aromatics), 6.88 (m, 2H, pyrrole), 6.25 (m, 2H, pyrrole), 4.38 (d, 1H, CH^α *t*Leu), 2.59 (d, 3H, CH$_3$ methyl amide), 2.09 (s, 3H, CH$_3$ pyrazole), 0.93 (s, 9H, CH$_3$ x 3 *t*Leu). LRMS calcd. for $C_{22}H_{26}ClN_5O_2$: 427.2; found: 450.1 [M+Na]$^+$.
35: RS2708-Val-NHCH$_3$ ((S)-methyl 3-methyl-2-(1-phenyl-5-(1H-pyrrol-1-yl)-1H-pyrazole-3-carboxamido)butanoate) was obtained in 89% yield, after reaction work up. R$_t$ (analytical RP-HPLC) = 20.77 min. ^{1}H NMR (300 MHz, DMSO-d$_6$) δ 8.12 (q, 1H, NH methyl amide), 7.85 (d, 1H, NH amide), 7.44-7.40 (m, 3H, phenyl aromatics), 7.24-7.21 (m, 2H, phenyl aromatics), 6.98 (s, 1H, pyrazole), 6.83 (m, 2H, pyrrole), 6.19 (m, 2H, pyrrole), 4.29 (t, 1H, CH^α Val), 2.59 (d, 3H, CH$_3$ methyl-amide), 2.04-2.02 (m, 1H, CH^β Val), 0.85 (dd, 6H, CH$_3$ x 2 Val). LRMS calcd. for $C_{20}H_{23}N_5O_2$: 365.2; found: 388.1 [M+Na]$^+$.
36: RS2708-*t*Leu-NHCH$_3$ (S)-N-(3,3-dimethyl-1-(methylamino)-1-oxobutan-2-yl)-1-phenyl-5-(1H-pyrrol-1-yl)-1H-pyrazole-3-carboxamide) was obtained in 56% yield, after reaction work up. R$_t$ (analytical RP-HPLC) = 21.61 min. ^{1}H NMR (300 MHz, DMSO-d$_6$) δ 8.20 (q, 1H, NH

methyl amide), 7.57 (d, 1H, NH amide), 7.45-7.21 (m, 5H, phenyl aromatics), 6.98 (s, 1H, pyrazole), 6.83 (m, 2H, pyrrole), 6.19 (m, 2H, pyrrole), 4.38 (d, 1H, CH^{α} *t*Leu), 2.59 (d, 3H, CH_3 methyl amide), 0.92 (s, 9H, CH_3 x 3 *t*Leu). LRMS calcd. for $C_{21}H_{25}N_5O_2$: 379.2; found: 402.2 $[M+Na]^+$.

37: RS2709-Val-$NHCH_3$ ((S)-4-methyl-N-(3-methyl-1-(methylamino)-1-oxobutan-2-yl)-1-phenyl-5-(1H-pyrrol-1-yl)-1H-pyrazole-3-carboxamide) was obtained in 76% yield, after reaction work up. R_t (analytical RP-HPLC) = 21.59 min. ^1H NMR (300 MHz, DMSO-d_6) δ 8.11 (q, 1H, NH methyl amide), 7.78 (d, 1H, NH amide), 7.37 (m, 3H, phenyl aomatics), 7.16 (m, 2H, phenyl aromatics), 6.86 (m, 2H, pyrrole), 6.23 (m, 2H, pyrrole), 4.30 (t, 1H, CH^{α} Val), 2.59 (d, 3H, CH_3 methyl amide), 2.09-2.02 (m, 4H, CH_3 pyrazole + 1H CH^{β} Val), 0.87 (dd, 6H, CH_3 x 2 Val). LRMS calcd. for $C_{21}H_{25}N_5O_2$: 379.2; found: 402.2 $[M+Na]^+$.

38: RS2709-*t*Leu-$NHCH_3$ (S)-N-(3,3-dimethyl-1-(methylamino)-1-oxobutan-2-yl)-4-methyl-1-phenyl-5-(1H-pyrrol-1-yl)-1H-pyrazole-3-carboxamide). The desired compound was obtained in 71% yield, after reaction work up. R_t (analytical RP-HPLC) = 22.62 min. ^1H NMR (300 MHz, DMSO-d_6) δ 8.18 (q, 1H, NH methyl amide), 7.59 (d, 1H, NH amide), 7.39-7.35 (m, 3H, phenyl aromatics), 7.17-7.14 (m, 2H, phenyl aromatics), 6.86 (m, 2H, pyrrole), 6.23 (m, 2H, pyrrole), 4.36 (d, 1H, CH^{α} *t*Leu), 2.59 (s, 3H, CH_3 methyl amide), 2.09 (s, 3H, CH_3 pyrazole), 0.93 (s, 9H, CH_3 x 3 *tert*-Leu). LRMS calcd. for $C_{22}H_{27}N_5O_2$: 393.2; found: 416.9 $[M+Na]^+$.

39: RS3516-Val-$NHCH_3$ 5-(2-chloro-1H-pyrrol-1-yl)-1-(2,4-dichlorophenyl)-4-methyl-N-((S)-3-methyl-1-(methylamino)-1-oxobutan-2-yl)-1H-pyrazole-3-carboxamide). The desired compound was obtained in 88% yield, after reaction work up. R_t (analytical RP-HPLC) = 23.91 min. ^1H NMR (300 MHz, DMSO-d_6) δ 8.11 (q, 1H, NH methyl amide), 7.85 (d, 1H, NH amide), 7.76-7.55 (m, 3H phenyl aromatics), 6.95 (m, 1H, pyrrole), 6.20 (m, 2H, pyrrole), 4.27 (t, 1H, CH^{α} Val), 2.58 (d, 3H, CH_3 methyl amide), 2.07-1.94 (m, 4H, CH_3 pyrazole + 1H CH^{β} Val), 0.84 (dd, 6H, CH_3 x 2 Val). LRMS calcd. for $C_{21}H_{22}Cl_3N_5O_2$: 481.1; found: 504.1 $[M+Na]^+$.

40: RS3516-*t*Leu-$NHCH_3$ 5-(2-chloro-1H-pyrrol-1-yl)-1-(2,4-dichlorophenyl)-N-((S)-3,3-dimethyl-1-(methylamino)-1-oxobutan-2-yl)-4-methyl-1H-pyrazole-3-carboxamide). The desired compound was obtained in 57% yield, after reaction work up. R_t (analytical RP-HPLC) = 24.80 min. ^1H NMR (300 MHz, DMSO-d_6) δ 8.18 (q, 1H, NH methyl amide), 7.85 (d, 1H, NH amide), 7.68-7.49 (m, 3H phenyl aromatics), 6.97-6.93 (m, 2H, pyrrole), 6.19 (m, 1H, pyrrole), 4.38 (d,

1H, CH$^{\alpha}$ *t*Leu), 2.59 (d, 3H, CH_3 methyl amide), 2.07 (s, 3H, CH_3 pyrazole), 0.92 (s, 9H, CH_3 x 3 *t*Bu). LRMS calcd. for $C_{22}H_{24}Cl_3N_5O_2$: 495.1; found: 518.2 $[M+Na]^+$.

Table S1. Sequence and structures of compounds **1-10/21-30**.

**Code**	**Sequence**	**R**	**R^1**	**R^2**	**R^3**	**R^4**	**Code**	**Sequence**	**R**	**R^1**	**R^2**	**R^3**	**R^4**
**1**	RS2689-Val-OH	H	H	Cl	H	H	**21**	RS2689-Val-OCH_3	H	H	Cl	H	H
**2**	RS2689-*t*Leu-OH	H	H	Cl	H	CH_3	**22**	RS2689-*t*Leu-OCH_3	H	H	Cl	H	CH_3
**3**	RS2691-Val-OH	CH_3	H	Cl	H	H	**23**	RS2691-Val-OCH_3	CH_3	H	Cl	H	H
**4**	RS2691-*t*Leu-OH	CH_3	H	Cl	H	CH_3	**24**	RS2691-*t*Leu-OCH_3	CH_3	H	Cl	H	CH_3
**5**	RS2708-Val-OH	H	H	H	H	H	**25**	RS2708-Val-OCH_3	H	H	H	H	H
**6**	RS2708-*t*Leu-OH	H	H	H	H	CH_3	**26**	RS2708-*t*Leu-OCH_3	H	H	H	H	CH_3
**7**	RS2709-Val-OH	CH_3	H	H	H	H	**27**	RS2709-Val-OCH_3	CH_3	H	H	H	H
**8**	RS2709-*t*Leu-OH	CH_3	H	H	H	CH_3	**28**	RS2709-*t*Leu-OCH_3	CH_3	H	H	H	CH_3
**9**	RS3516-Val-OH	CH_3	Cl	Cl	Cl	H	**29**	RS3516-Val-OCH_3	CH_3	Cl	Cl	Cl	H
**10**	RS3516-*t*Leu-OH	CH_3	Cl	Cl	Cl	CH_3	**30**	RS3516-*t*Leu-OCH_3	CH_3	Cl	Cl	Cl	CH_3

Table S2. Sequence and structures of compounds **11-20**.

Code	Sequence	R	R^1	R^2	R^3	R^4
**11**	RS2689-Val-NH_2	H	H	Cl	H	H
**12**	RS2689-*t*Leu-NH_2	H	H	Cl	H	CH_3
**13**	RS2691-Val-NH_2	CH_3	H	Cl	H	H
**14**	RS2691-*t*Leu-NH_2	CH_3	H	Cl	H	CH_3
**15**	RS2708-Val-NH_2	H	H	H	H	H
**16**	RS2708-*t*Leu-NH_2	H	H	H	H	CH_3
**17**	RS2709-Val-NH_2	CH_3	H	H	H	H
**18**	RS2709-*t*Leu-NH_2	CH_3	H	H	H	CH_3
**19**	RS3516-Val-NH_2	CH_3	Cl	Cl	Cl	H
**20**	RS3516-*t*Leu-NH_2	CH_3	Cl	Cl	Cl	CH_3

Table S3. Sequence and structures of compounds **31-40**.

Code	Sequence	R	R^1	R^2	R^3	R^4
**31**	RS2689-Val-$NHCH_3$	H	H	Cl	H	H
**32**	RS2689-*t*Leu-$NHCH_3$	H	H	Cl	H	CH_3
**33**	RS2691-Val-$NHCH_3$	CH_3	H	Cl	H	H
**34**	RS2691-*t*Leu-$NHCH_3$	CH_3	H	Cl	H	CH_3
**35**	RS2708-Val-$NHCH_3$	H	H	H	H	H
**36**	RS2708-*t*Leu-$NHCH_3$	H	H	H	H	CH_3
**37**	RS2709-Val-$NHCH_3$	CH_3	H	H	H	H
**38**	RS2709-*t*Leu-$NHCH_3$	CH_3	H	H	H	CH_3
**39**	RS3516-Val-$NHCH_3$	CH_3	Cl	Cl	Cl	H
**40**	RS3516-*t*Leu-$NHCH_3$	CH_3	Cl	Cl	Cl	CH_3

In Vitro Biological Assays

Preparation of Brain Membrane Homogenates

Wistar rats were locally bred and handled according to the EU Directive 2010/63/EU and to the Regulations on Animal Protection (40/2013. (II. 14.) Korm. r.) of Hungary. Crude membrane fractions were prepared from the brain. Brains were quickly removed from the euthanized rats and directly put in an ice-cold 50 mM Tris-HCl buffer (pH 7.4). The collected tissue was then homogenized in 30 volumes (v/w) of an ice-cold buffer with a Braun Teflon-glass homogenizer at the highest rpm. The homogenate was centrifuged at 20,000 × g for 25 min, and the resulting pellet was suspended in the same volume of a cold buffer followed by incubation at 37 °C for 30 min to remove endogenous ligands. Centrifugation was then repeated. The final pellets were taken up in five volumes of a 50 mM Tris-HCl (pH 7.4) buffer containing 0.32 M sucrose and stored at -80 °C. Prior to the experiment, aliquots were thawed and centrifuged at 20,000 ×g for 25 min and then they were resuspended in 50 mM Tris-HCl (pH 7.4), homogenized with a Douncer, followed by the determination of the protein concentration by the method of Bradford. The membrane suspensions were immediately used either in radioligand binding experiments or in [^{35}S]GTPγS functional assays.

Radioligand Competition Binding Assay

Binding experiments were performed at 30 °C for 60 min in a 50 mM Tris-HCl binding buffer (pH 7.4) containing 2.5 mM of EGTA, 5 mM of $MgCl_2$ and 0.5 mg/mL of fatty acid-free BSA in plastic tubes in a total assay volume of 1 mL that contained 0.3-0.5 mg/mL of a membrane protein. Competition binding experiments were carried out by incubating rat brain membranes with 5 nM of [^{3}H]WIN55,212-2 (Kd: 10.1 nM) in the presence of increasing concentrations (10^{-11}-10^{-5} M) of various competing unlabelled ligands. Non-specific binding was determined in the presence of 10 μM of WIN 55,212-2. The incubation was terminated by diluting the samples with an ice-cold wash buffer (50 mM of Tris-HCl, 2.5 mM of EGTA, 5 mM of $MgCl_2$, 0.5% fatty acid free BSA, pH 7.4), followed by repeated washing and rapid filtration through Whatman GF/B glass fiber filters (Whatman Ltd., Maidstone, UK) pre-soaked with 0.1% polyethyleneimine (30 min before the filtration). Filtration was performed with a 24-well Brandel Cell Harvester (Gaithersburg, MD, USA). Filters were air-dried and immersed into Ultima Gold MV scintillation cocktail, and then radioactivity was measured with a TRI-CARB 2100TR liquid scintillation analyzer (Packard, Perkin Elmer, Waltham, MA, USA).

Ligand Stimulated [^{35}S]GTPγS Binding Assay

Rat brain membranes (30 μg protein/tube), prepared as described above, were incubated with 0.05 nM of [^{35}S]GTPγS (PerkinElmer) and 10^{-10}-10^{-5} M unlabelled ligands in the presence of 30 μM of GDP, 100 mM of NaCl, 3 mM of $MgCl_2$ and 1 mM of EGTA in a 50 mM Tris-HCl buffer (pH 7.4) for 60 min at 30 °C. Basal [^{35}S]GTPγS binding was measured in the absence of ligands and set as 100%. Nonspecific

binding was determined by the addition of 10 μM unlabelled GTPγS and subtracted from total binding. Incubation, filtration and radioactivity measurements of the samples were carried out as described above.

Data Analysis

The results of the competition binding studies are reported as means ± S.E.M. of at least three independent experiments each performed in duplicate. In competition binding studies, the inhibitory constants (Ki) were calculated from the inflection points of the displacement curves using non-linear least-square curve fitting and the Cheng-Prusoff equation, Ki= EC_{50}/(1 + [ligand]/Kd). In [^{35}S]GTPγS binding studies, data were expressed as the percentage stimulation of the specific [^{35}S]GTPγS binding over the basal activity and are given as means ±S.E.M. Each experiment was performed in triplicate and analyzed with sigmoid dose-response curve fitting to obtain potency (EC_{50}) and efficacy (E_{max}) values. All data and curves were analyzed by GraphPad Prism 5.0 (San Diego, CA, USA).

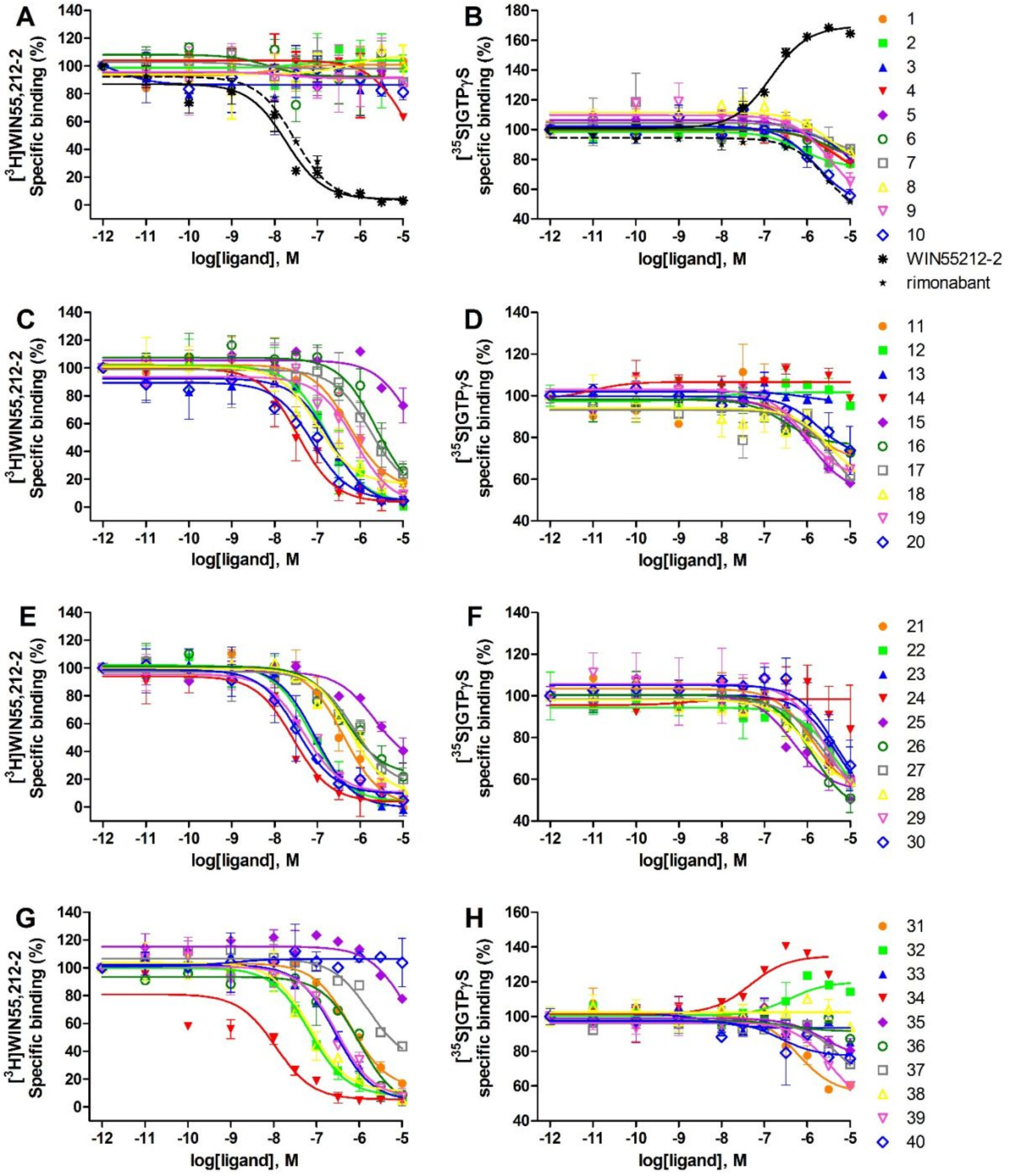

Figure S1. On the left: Cannabinoid receptor binding affinity of compounds **1-40** (A, C, E, G) in [3H]WIN55,212-2 displacement binding assays to whole brain rat membrane homogenates. The graphics represent the specific binding of the radioligand in percentage in the presence of increasing concentrations (10^{-11}-10^{-5} M) of the ligands indicated on the right. Data are expressed as percentage of mean specific binding ± S.E.M. (n≥ 3). The affinity values (Ki) of the unlabeled compounds are indicated in Table 1. On the right: G protein stimulatory effects of the **1-40** (B, D, F, H) in [^{35}S]GTPγS stimulation assays in homogenates of rat brain membrane. Graphics represent relative specific binding of [^{35}S]GTPγS in the presence of increasing concentrations (10^{-11}-10^{-5} M) of the novel compounds indicated in the legend on the right. Data are expressed as percentage of mean specific binding ± S.E.M. (n≥ 3). The maximum efficacy (E_{max}) and potency (EC_{50}) values of the unlabeled compounds are indicated in Table 1 in the main manuscript.

In Vivo Biological Assays

Animals

The international and national law and policies approved by Italian Ministry of Health were used to comply with all animal care and experimental procedures. Animal studies were advised in compliance with the ARRIVE guidelines and with the recommendations made by EU Directive 2010/63/EU for animal experiments and the Basel declaration including the 3Rs concept [1,2]. CD-1 male mice (10-14 weeks of age, 25-30 g of weight) were bought from Charles River (Milan, Italy). Shortly after their arrival and for at least one week, they were kept in an animal care facility under controlled standard conditions of temperature (21 ± 1 °C), light (from 7:00 AM to 7:00 PM), and relative humidity (60 ± 10%). Access to drinking water and food was assured. All procedures were performed to decrease the number of animals used (n = 7 per group) and their distress.

Feeding Test

The test was carried out as previously described [3]. At 24 h before the start of a feeding test, all food was removed from the home cages of mice to be tested. The next day and at least 1 h before the feeding test began, the mice were transported to the laboratory. On test days, the animals were placed in the home cages for 30 min of drug assimilation, during which food was not available. Then compounds were intraperitoneally administered (10 mg/kg). Mice were transferred into transparent and individual plastic cages with thick white paper lining the bottom and access to a pre-measured amount of their regular lab chow (2 gr) for the 1-h test. At the end of 1 h, mice were repositioned into their home cage. The amount of food left in the trial cage, including crumbs, was measured, and the amount consumed was calculated. Feeding trials normally happened on Tuesdays and Fridays between 12:00 and 14:00 h.

Hot plate Test

Thermal nociception (hot-plate test) was assessed with a commercially available apparatus consisting of a metal plate 25x25 cm (Ugo Basile, Italy) heated to a constant temperature of 55.0 ± 0.1 °C, on which a plastic cylinder (20 cm diameter, 18 cm high) was placed. The time of latency (s) was recorded from the moment the animal was placed in the cylinder on the hot plate until it licked its paws or jumped; the cut-off time was 60 s. The baseline was calculated as mean of three readings recorded before testing at intervals of 15 min, and was in the same order of magnitude in all experimental groups (mean 9.8 ± 1.2 s, n = 7). The time course of latency was then determined at 15, 30, 45, 60, 90 and 120 min after compound treatment. Experimental data were expressed as time course of the percentage of maximum effect (%MPE) ¼ (post-drug latency – baseline latency)/(cut-off time-baseline latency)x100. Compounds were freshly diluted in saline 0.1% v/v DMSO and were injected at 10 μg/10 μL for intracerebroventricular (i.c.v.) administrations, as previously reported [4].

Formalin Test

The method utilized was comparable to the one previously described by Pieretti *et al*. [5]. Mice were located to adapt into the transparent cages individually (30 × 14 × 12 cm) for at least an hour before testing. They were injected with 20 µL of a 1% solution of formalin in saline. Then, the compounds were administered subcutaneously in the dorsal surface of the right hind paw of the mouse using a microsyringe with a 27-gauge needle for 15 min before. Compounds were prepared by freshly diluting saline containing 0.9% NaCl in the ratio DMSO:saline 1:3 (v/v). Then, these solutions were injected for subcutaneous (s.c.) administrations in doses of 30-100 µg/20 µL. The total time the animal spent licking or biting its paw was calculated.

Edema Induced by Zymosan

In this test, 100 µg of the compounds were administered subcutaneously in a volume of 20 µL in the dorsal surface of mice hind paw; this was done 15 min before a subcutaneous injection (20 µL/paw) of zymosan A (2.5% w/v in saline) into the same paw. Then, paw edema was calculated as formerly described [6]. The percentage difference between the paw volume at each time point and the basal paw volume was used as an index of the increase in paw volume. Paw volume was quantified using a hydroplethysmometer modified for small volumes (Ugo Basile, Varese, Italy) three times before the injections and at 1, 2, 3, 4 and 24 h thereafter.

Data Analysis and Statistics

The mean ± S.E.M. was used to explain the results obtained. Statistically significant differences between groups were measured with an analysis of variance (ANOVA) followed by Tukey's post-hoc comparisons or the Mann-Whitney test when the comparison was restricted to two groups. GraphPad Prism 6.0 software (San Diego, CA, USA) was used to analyze the data. Data were considered statistically significant when a value of $p < 0.05$ was performed. The data and statistical analyses respected the recommendations on experimental design and analysis.

In silico study

Ligand preparation

The 3D molecular structure of the ligand was built with the Schrödinger software. The energy minimization was performed using the OPLS-2005 force field [7]. Then, the compound was prepared by Ligprep module [8]. To consider the flexibility of both ligand and receptor in the docking study, the IFD42 protocol was adopted. In IFD protocol, ligands were docked into the rigid protein using the soften-potential docking in the Glide program with the van der Waals radii scaling of 0.8 for the proteins. The resulted top 20 poses of each ligand were then used to sample the protein plasticity using the Prime program in the Schrödinger suite. Residues having at least one atom within 5 Å of any of the 20 ligand poses were subject to a conformational search and energy minimization process, although residues outside this zone were fixed. In this way, the flexibility of protein was considered. The resulted 20 new receptor conformations were taken forward for re-docking, Glide docking parameters were set to the default hard-potential function. The Glide XP (extra precision) was used for all the docking calculations. The binding affinity of each complex was reported as Glide Score.

QM/MM (quantum mechanics/molecular mechanics) docking study

For the docking study the QM/MM method has been chosen since it was reported to have higher accuracy respect to other method such as Glide [9]. The QM/MM docking was performed by the Schrödinger QM-polarized ligand docking protocol (QPLD). It was validated first on the crystallographic pose. In the validation experiment QPLD the crystal ligand was docked into its native crystal structure using both QPLD and Glide's standard precision (SP) mode. QPLD method was the only able to accurately reproduce the crystallographic pose and original interactions reported by Kumar *et al*. [8].

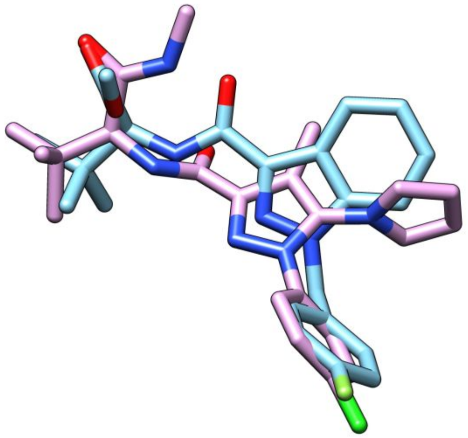

Figure S2. Validation of crystallographic poses.

The QM/MM docking was then performed on the novel agonist **34**. The fully automated algorithm of QPLD was carried out as follows: (*i*) Glide docking job generated several geometrically unique protein-ligand complexes (semiempirical method with Standar Precision), with ligand van der Waals scaling fixed at 0.8, the poses were discarded if the RMSD was less than 0.5A and if the maximum atomic displacement less than 1.3 A. Then (*ii*) QSite performed a single-point energy calculation on each complex, treating the ligand with ab initio methods and deriving partial atomic charges using electrostatic potential fitting, the charge of the ligand have been calculated in water, by using of the quantomechanical method Jaguar at the most accurate level. Jaguar is a high-performance *ab initio* package for both gas and solution phase simulations (*iii*) in this step, the top 5 poses of the ligand was used, Glide then re-docks the ligand using each of the ligand charge sets calculated by QSite, and the QPLD algorithm returns the ten most energetically favourable poses. The Emodel value was selected for scoring the poses.

References

1. Kilkenny, C., Browne, W., Cuthill, I.C., Emerson, M., Altman, D.G. Animal research: reporting in vivo experiments: the ARRIVE guidelines. Br J Pharmacol. 160 (**2010**) 1577-1579, doi: 10.1111/j.1476-5381.2010.00872.x.
2. McGrath, J.C., Lilley, E. Implementing guidelines on reporting research using animals (ARRIVE etc.): new requirements for publication in BJP. Br J Pharmacol. 172 (**2015**) 3189-3193, doi: 10.1111/bph.12955.
3. Wiley, J.L., Burston, J.J., Leggett, D.C., Alekseeva, O.O., Razdan, R.K., Mahadevan, A., Martin, B.R. CB1 cannabinoid receptor-mediated modulation of food intake in mice. Br J Pharmacol. 145 (**2005**) 293-300, doi: 10.1038/sj.bjp.0706157.
4. Monti, L., Stefanucci, A., Pieretti, S., Marzoli, F., Fidanza, L., Mollica, A., Mirzaie, S., Carradori, S., De Petrocellis, L., Schiano Moriello, A., Benyhe, S., Zádor, F., Szűcs, E., Ötvös, F., Erdei, A.I., Samavati, R., Dvorácskó, S., Tömböly, C., Novellino, E. Evaluation of the analgesic effect of 4-anilidopiperidine scaffold containing ureas and carbamates. J Enzyme Inhib Med Chem. 31 (**2016**) 1638-1647, doi: 10.3109/14756366.2016.1160902.
5. Pieretti, S., Di Giannuario, A., De Felice, M., Perretti, M., Cirino, G. Stimulus-dependent specificity for annexin 1 inhibition of the inflammatory nociceptive response: the involvement of the receptor for formylated peptides. Pain. 109 (**2004**) 52-63, doi: 10.1016/j.pain.2004.01.009.
6. Dimmito, M.P., Stefanucci, A., Pieretti, S., Minosi, P., Dvorácskó, S., Tömböly, C., Zengin, G., Mollica, A. Discovery of Orexant and Anorexant Agents with Indazole Scaffold Endowed with Peripheral Antiedema Activity. Biomolecules 9 (**2019**) 492, doi: 10.3390/biom9090492.

7. Stefanucci, A., Lei, W., Pieretti, S. *et al.* On resin click-chemistry-mediated synthesis of novel enkephalin analogues with potent anti-nociceptive activity. *Sci Rep* **9**, 5771 (2019). https://doi.org/10.1038/s41598-019-42289-5.
8. Krishna Kumar, K., Shalev-Benami, M., Robertson, M.J., Hu, H., Banister, S.D., Hollingsworth, S.A., Latorraca, N.R., Kato, H.E., Hilger, D., Maeda, S., Weis, W.I., Farrens, D.L., Dror, R.O., Malhotra, S.V., Kobilka, B.K., Skiniotis, G. Structure of a Signaling Cannabinoid Receptor 1-G Protein Complex. Cell 176 (**2019**) 448.
9. Burger, S.K., Thompson, D.C., Ayers, P.W. Quantum mechanics/molecular mechanics strategies for docking pose refinement: distinguishing between binders and decoys in cytochrome C peroxidase. J Chem Inf Model. 51 (**2011**) 93-101. doi: 10.1021/ci100329z.

^{1}H NMR SPECTRA OF FINAL COMPOUNDS IN DMSO-d_6

1

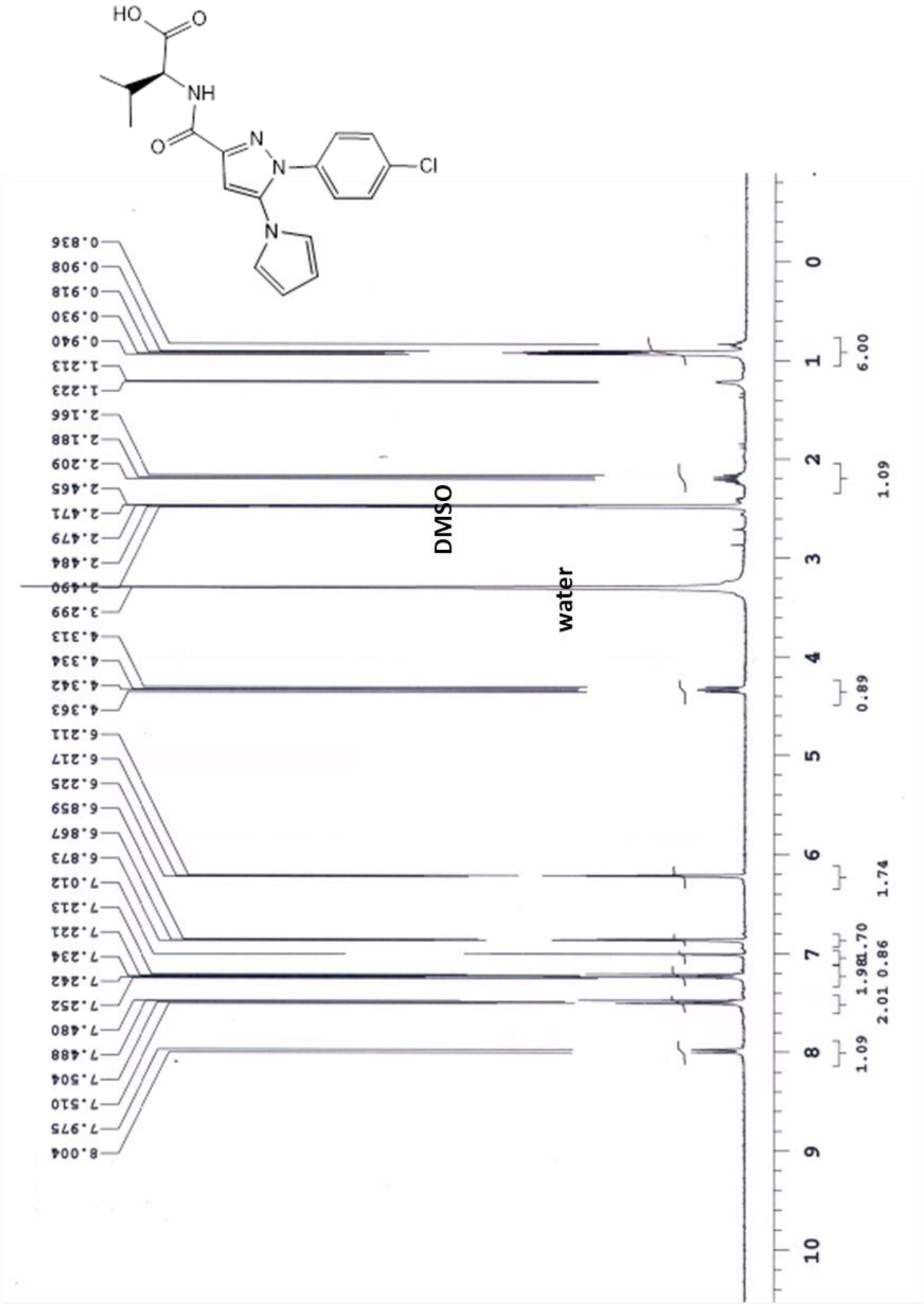

2

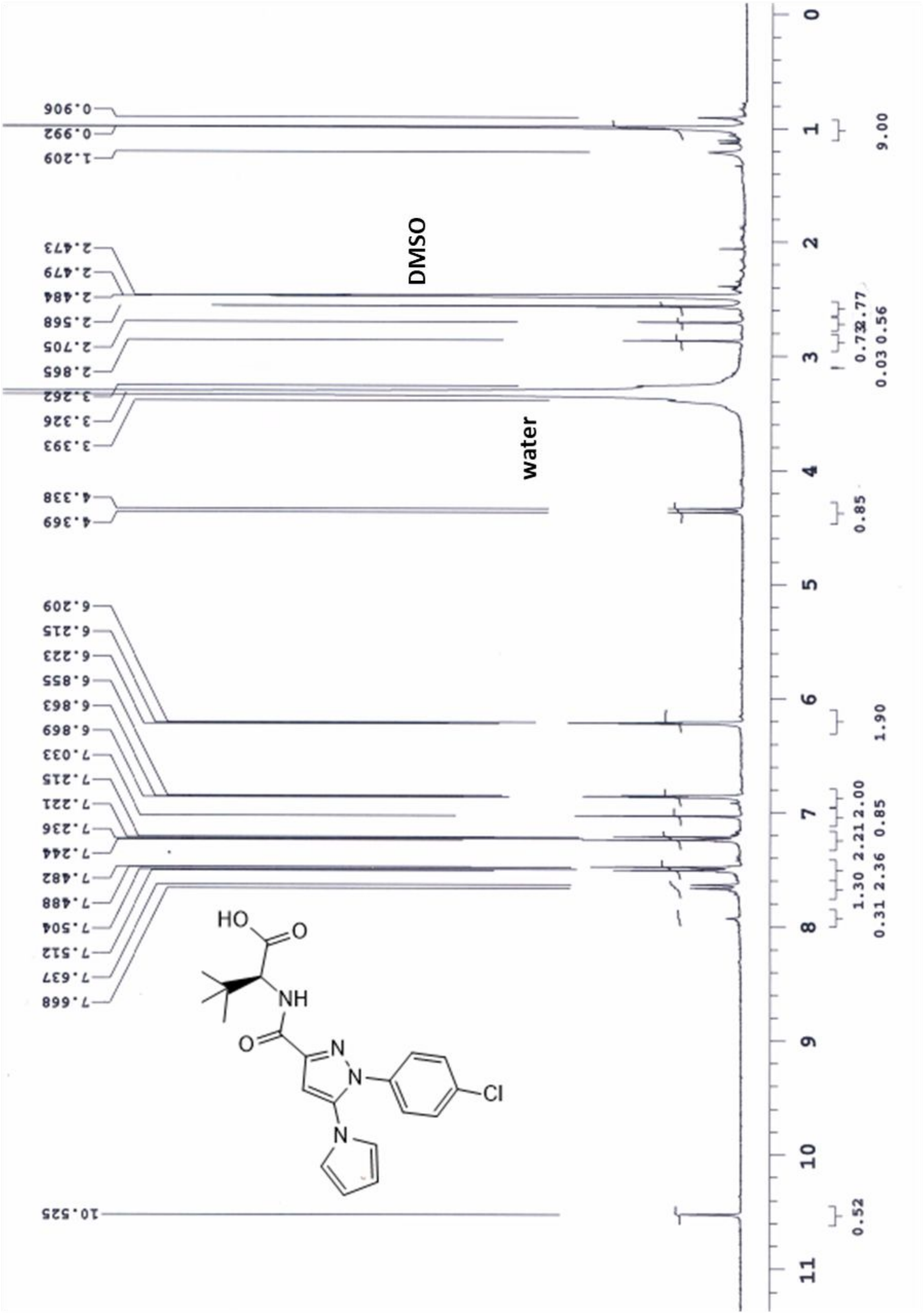
0.906
0.992
1.209
2.473
2.479
2.484
2.568
2.705
2.865
3.262
3.326
3.393
4.338
4.369
6.209
6.215
6.223
6.855
6.863
6.869
7.033
7.215
7.221
7.236
7.244
7.482
7.488
7.504
7.512
7.637
7.668
10.525
DMSO
water
HO
O
NH
O
N
N
Cl
N
9.00
0.85
1.90
0.52

3

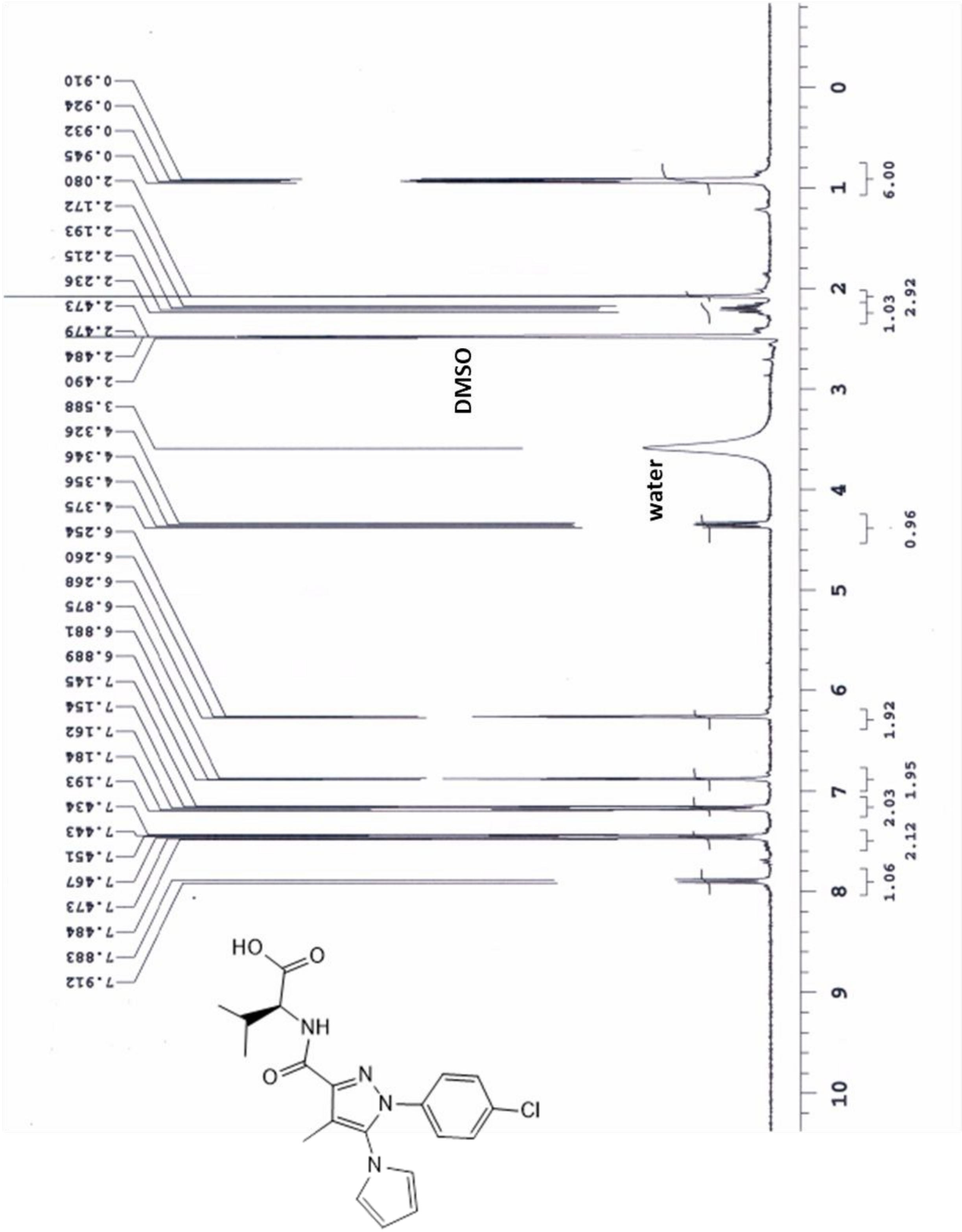
0.910
0.924
0.932
0.945
2.080
2.172
2.193
2.215
2.236
2.473
2.479
2.484
2.490
3.588
4.326
4.346
4.356
4.375
6.254
6.260
6.268
6.875
6.881
6.889
7.145
7.154
7.162
7.184
7.193
7.434
7.443
7.451
7.467
7.473
7.484
7.883
7.912
DMSO
water
6.00
1.03
2.92
0.96
1.92
2.03
1.95
1.06
2.12
0
1
2
3
4
5
6
7
8
9
10
HO
O
NH
O
N
N
N
Cl

4

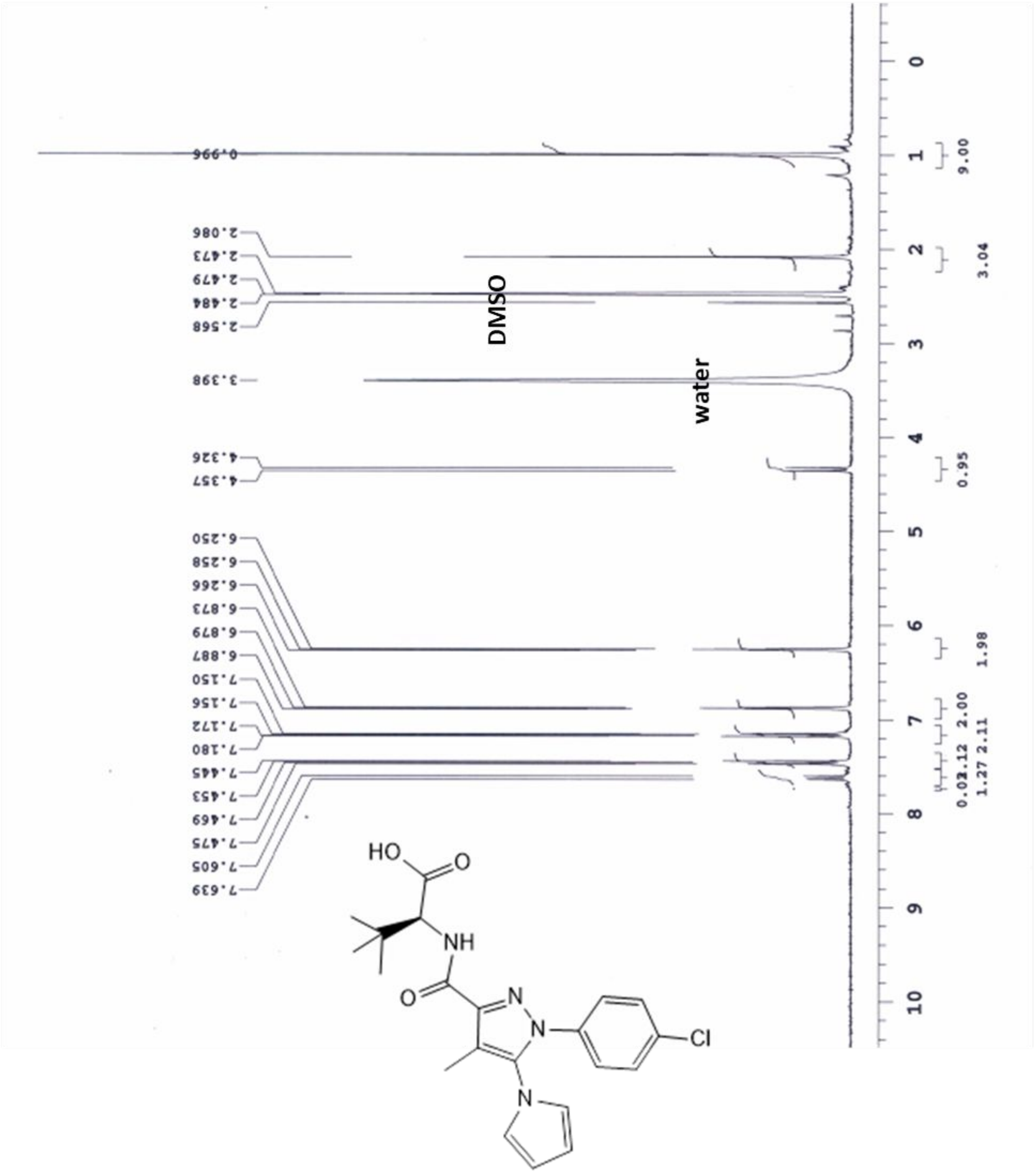

5

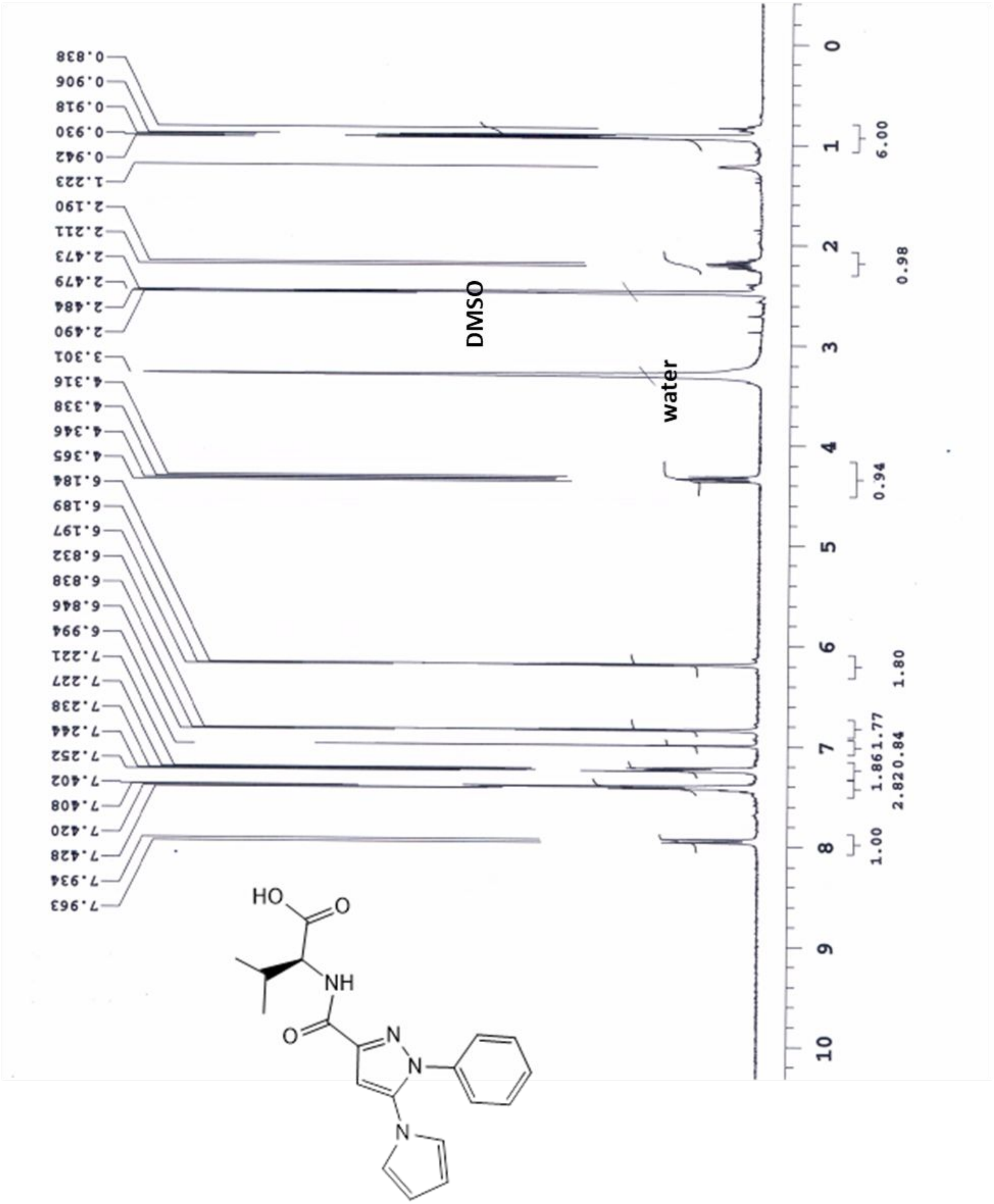
0.838
0.906
0.918
0.930
0.942
1.223
2.190
2.211
2.473
2.479
2.484
2.490
3.301
4.316
4.338
4.346
4.365
6.184
6.189
6.197
6.832
6.838
6.846
6.994
7.221
7.227
7.238
7.244
7.252
7.402
7.408
7.420
7.428
7.934
7.963
DMSO
water
6.00
0.98
0.94
1.80
1.86
1.77
2.82
0.84
1.00
HO
O
NH
O
N
N
N

6

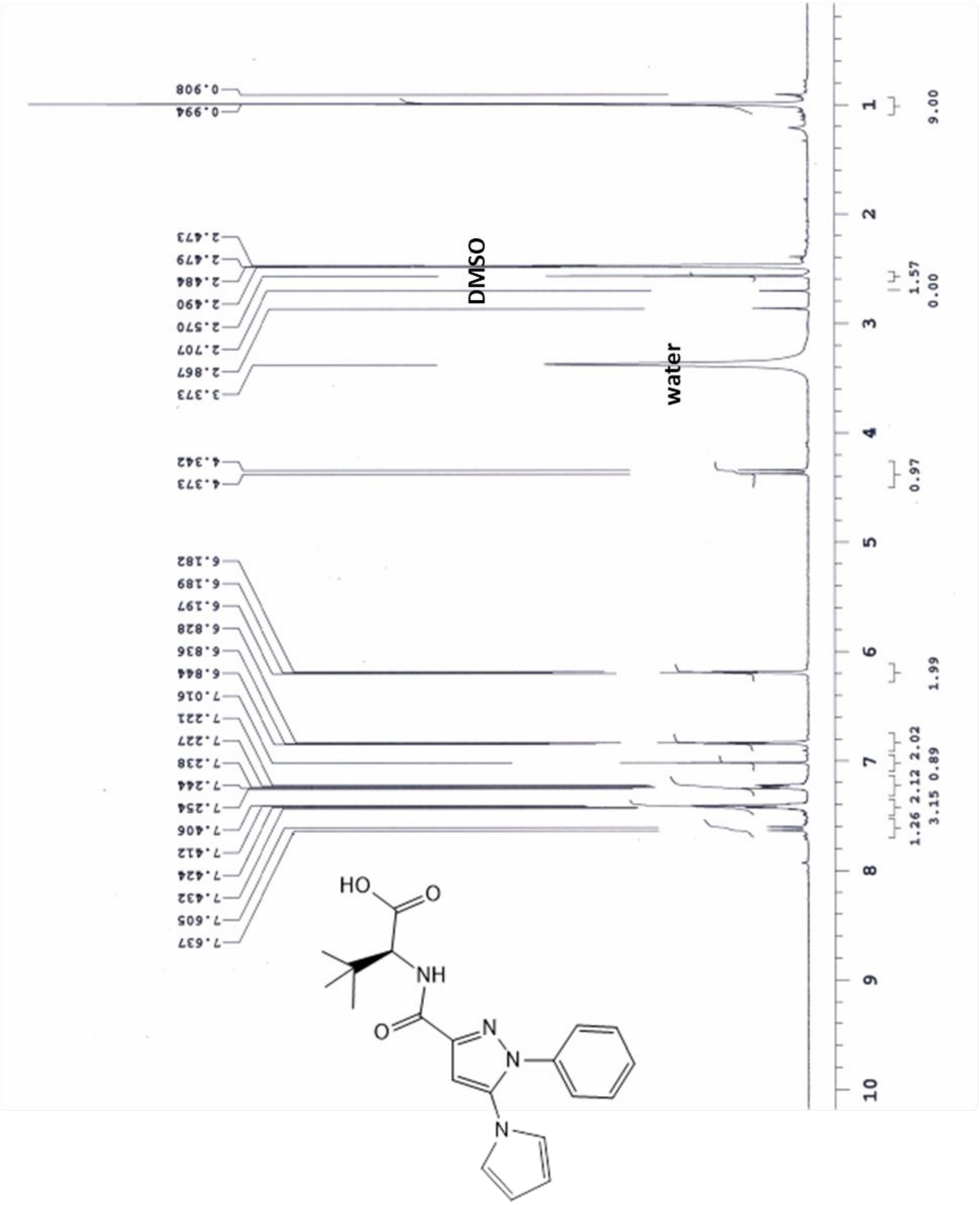

7

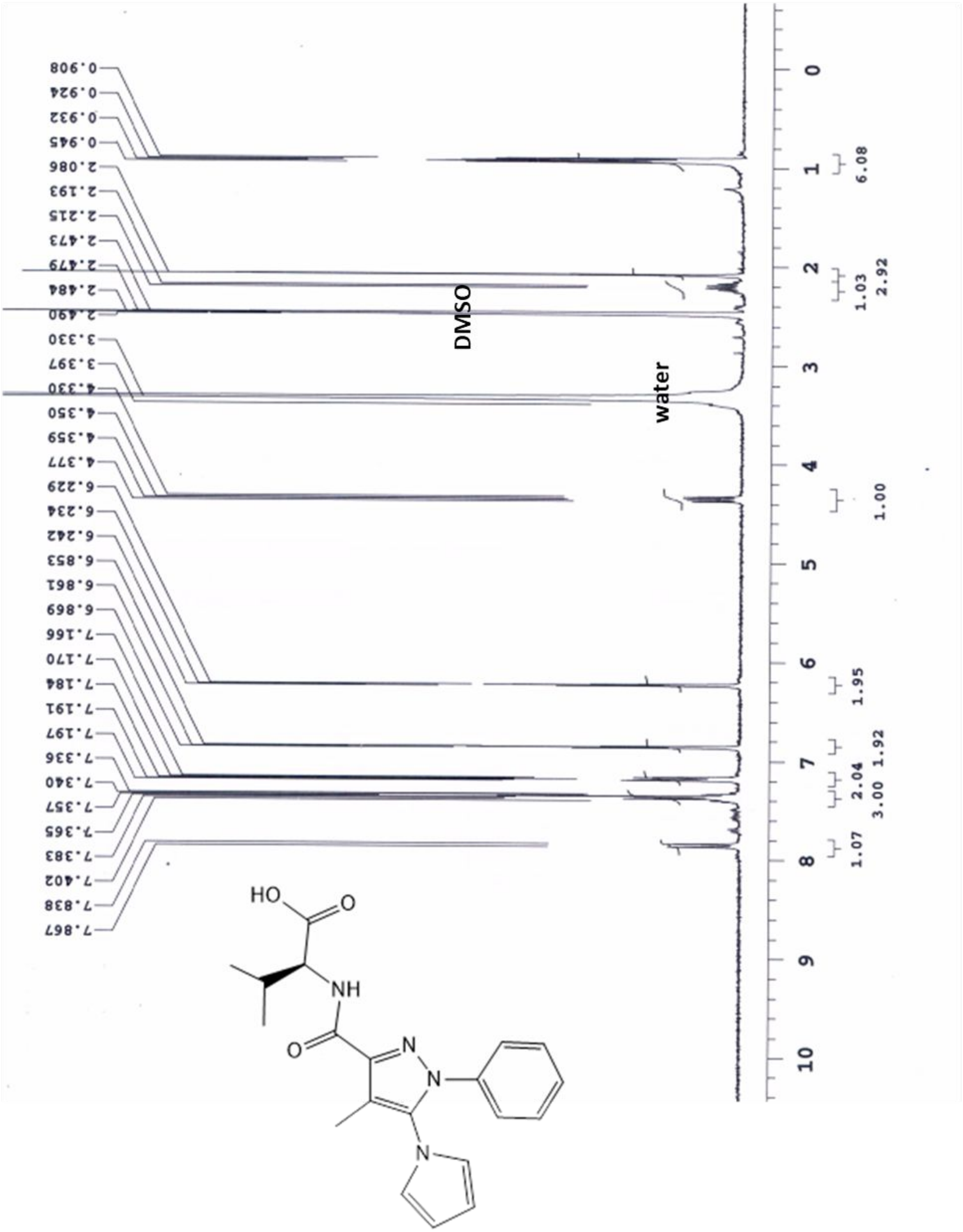

0.908
0.924
0.932
0.945
2.086
2.193
2.215
2.473
2.479
2.484
2.490
3.330
3.397
4.330
4.350
4.359
4.377
6.229
6.234
6.242
6.853
6.861
6.869
7.166
7.170
7.184
7.191
7.197
7.336
7.340
7.357
7.365
7.383
7.402
7.838
7.867
DMSO
water
6.08
1.03
2.92
1.00
1.95
1.92
2.04
3.00
1.07
0 1 2 3 4 5 6 7 8 9 10
HO
O
NH
O
N
N
N

8

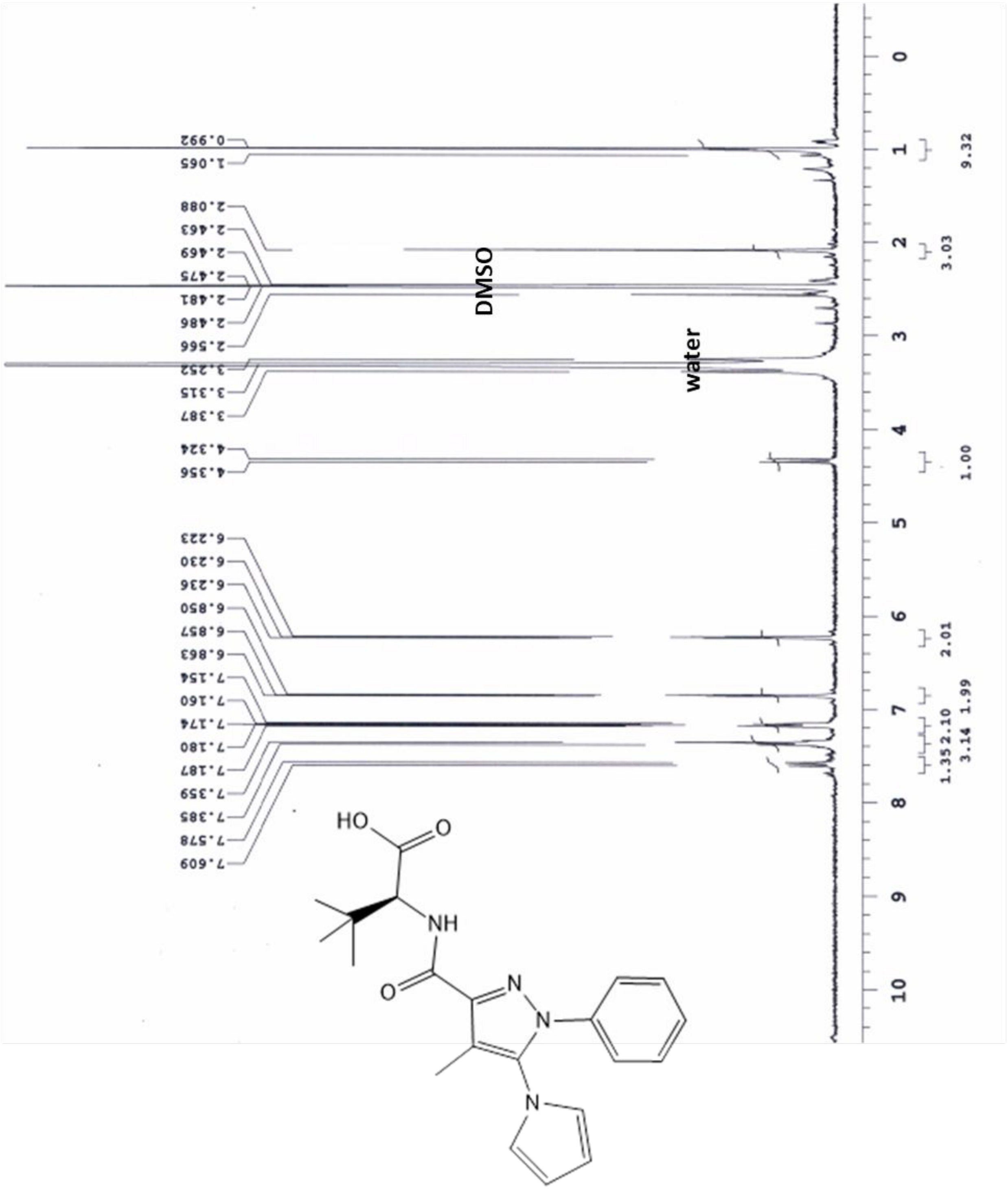

9

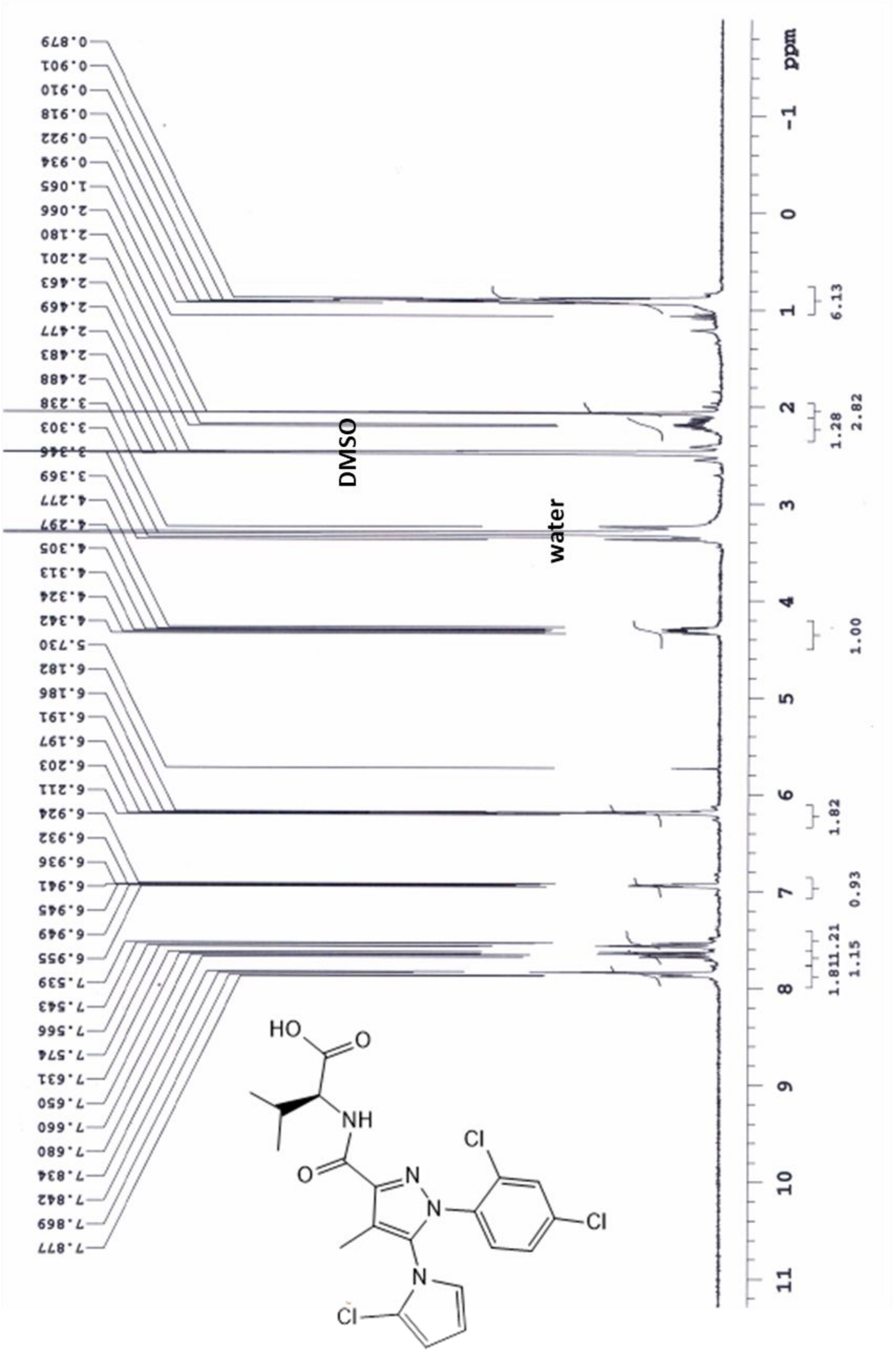

10

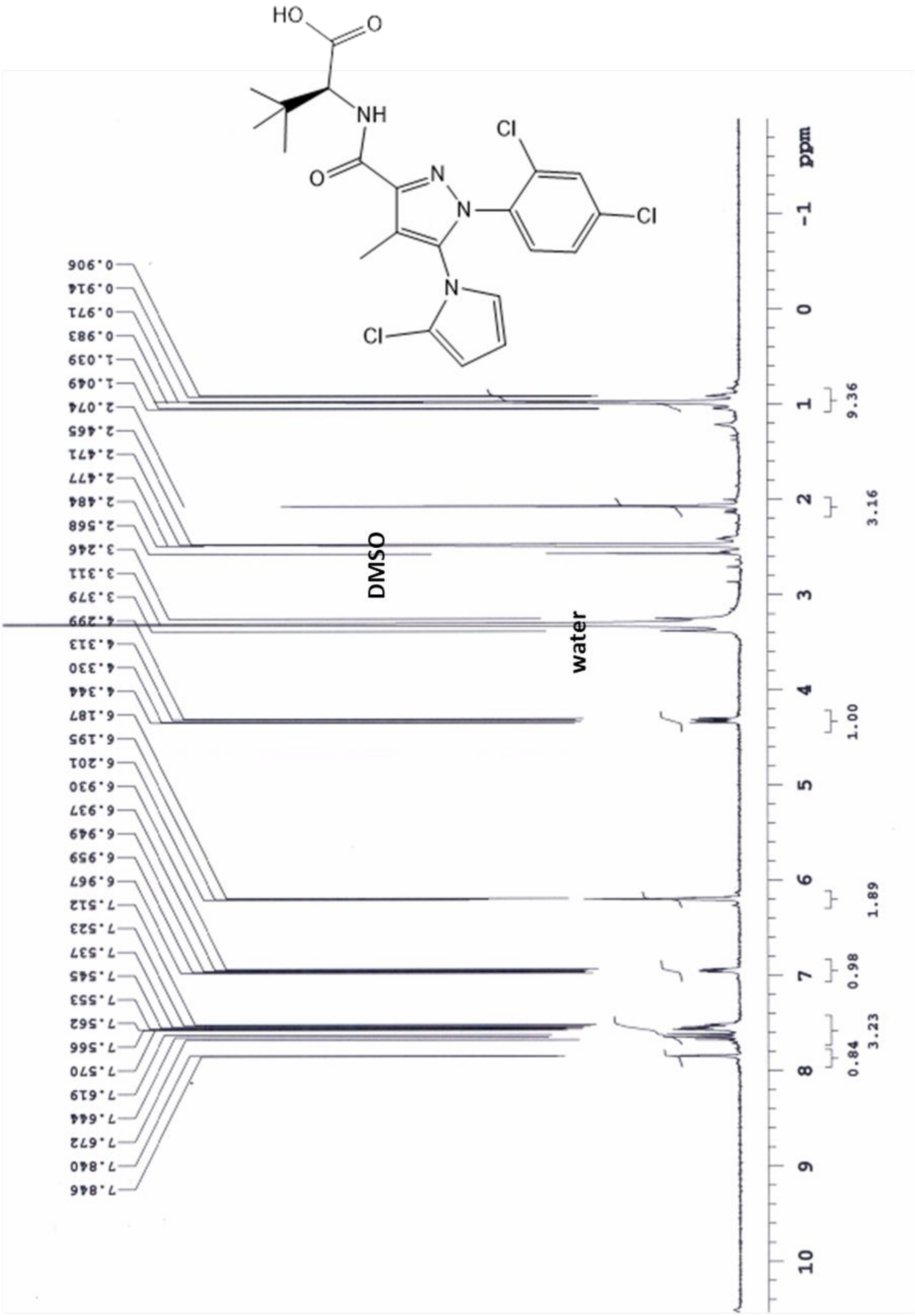
HO
O
NH
O
N
N
Cl
Cl
N
Cl
DMSO
water
ppm
0.906
0.914
0.971
0.983
1.039
1.049
2.074
2.465
2.471
2.477
2.484
2.568
3.246
3.311
3.379
4.299
4.313
4.330
4.344
6.187
6.195
6.201
6.930
6.937
6.949
6.959
6.967
7.512
7.523
7.537
7.545
7.553
7.562
7.566
7.570
7.619
7.644
7.672
7.840
7.846
9.36
3.16
1.00
1.89
0.98
0.84
3.23

11

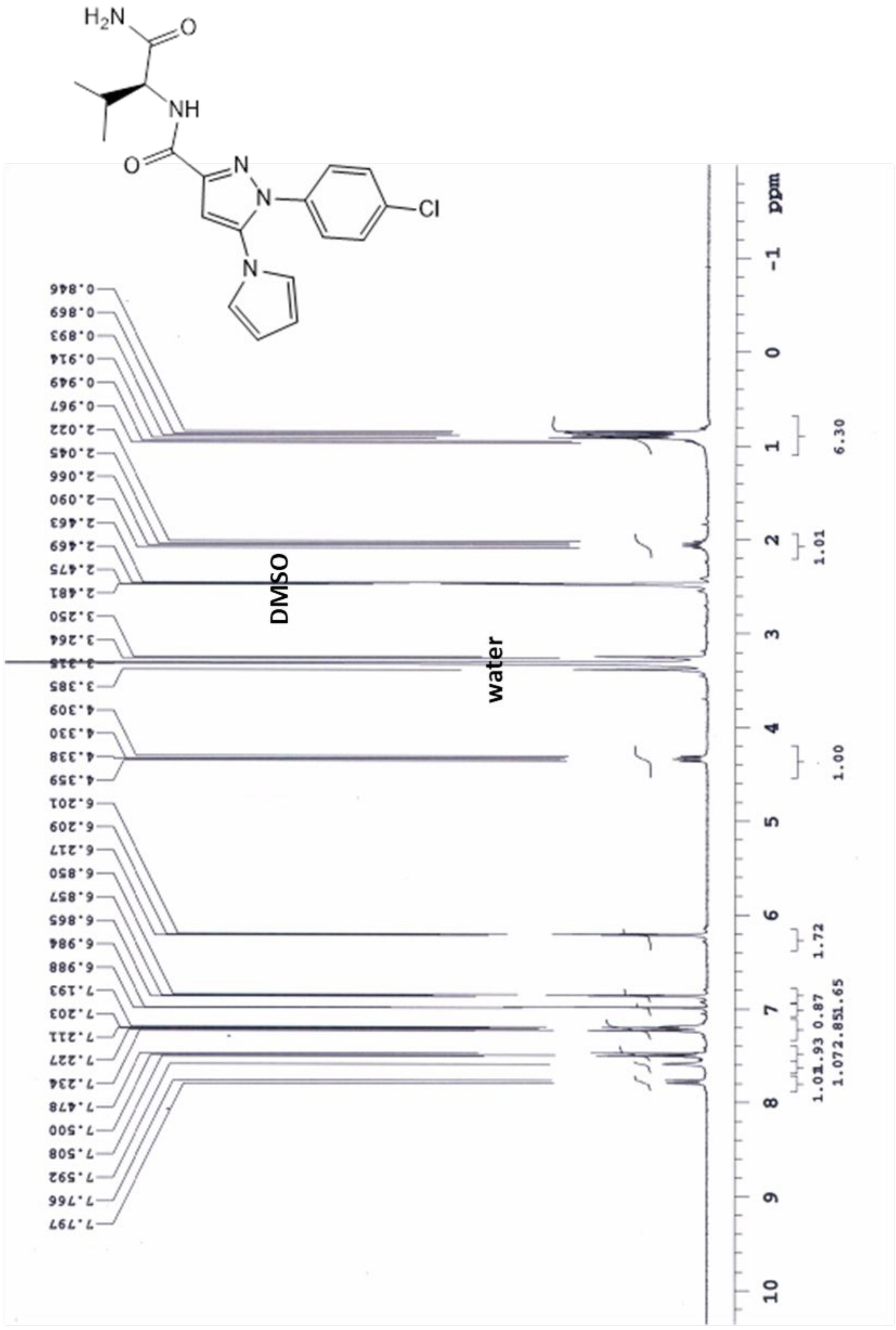

12

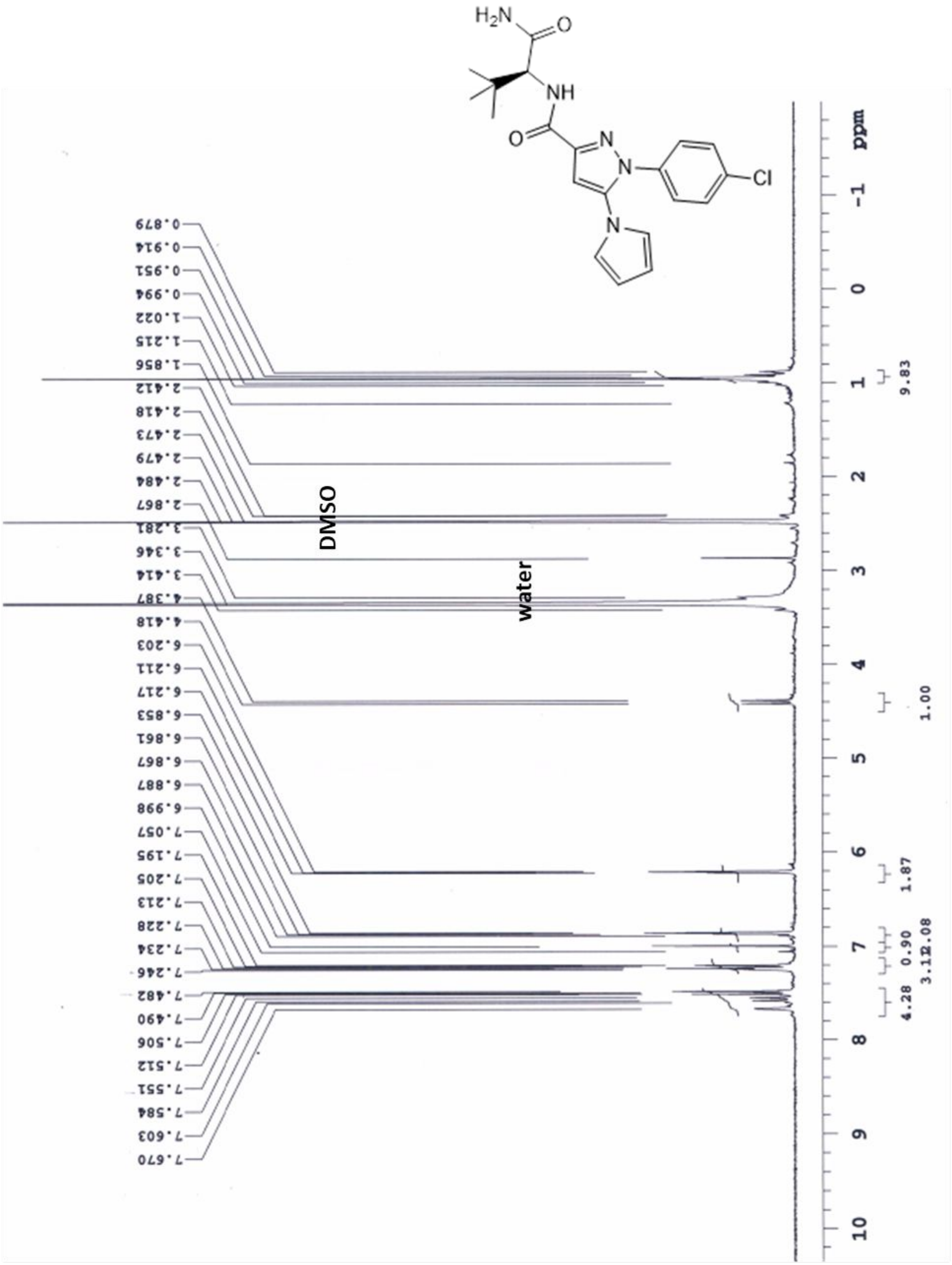
H2N
O
NH
O
N
N
Cl
N
DMSO
water
ppm
0.879
0.914
0.951
0.994
1.022
1.215
1.856
2.412
2.418
2.473
2.479
2.484
2.867
3.281
3.346
3.414
4.387
4.418
6.203
6.211
6.217
6.853
6.861
6.867
6.887
6.998
7.057
7.195
7.205
7.213
7.228
7.234
7.246
7.482
7.490
7.506
7.512
7.551
7.584
7.603
7.670
9.83
1.00
1.87
4.28
0.90
3.12
2.08

13

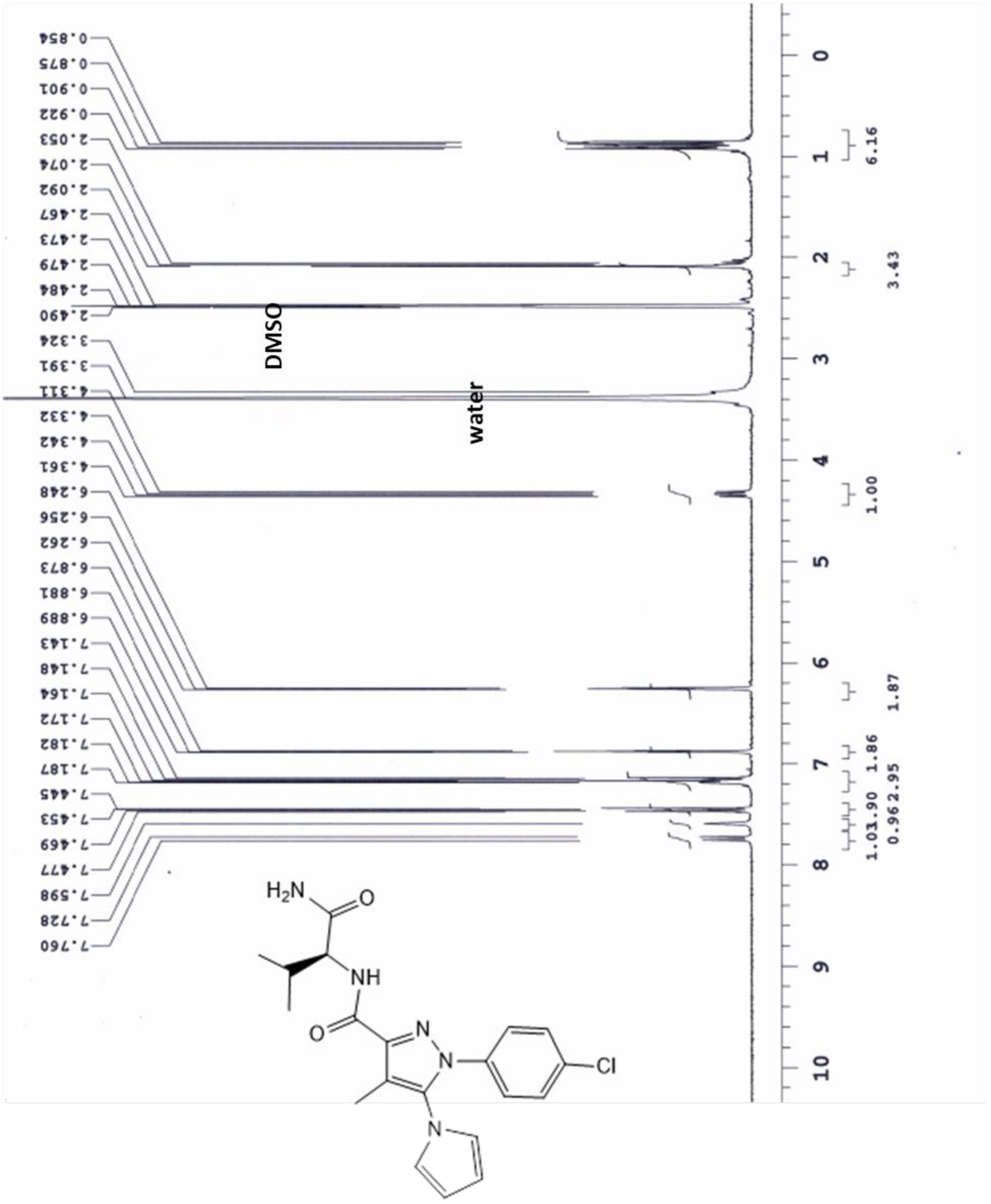
0.854
0.875
0.901
0.922
2.053
2.074
2.092
2.467
2.473
2.479
2.484
2.490
3.324
3.391
4.311
4.332
4.342
4.361
6.248
6.256
6.262
6.873
6.881
6.889
7.143
7.148
7.164
7.172
7.182
7.187
7.445
7.453
7.469
7.477
7.598
7.728
7.760
DMSO
water
6.16
3.43
1.00
1.87
1.01.90 1.86
0.962.95
H2N
O
NH
N
Cl

14

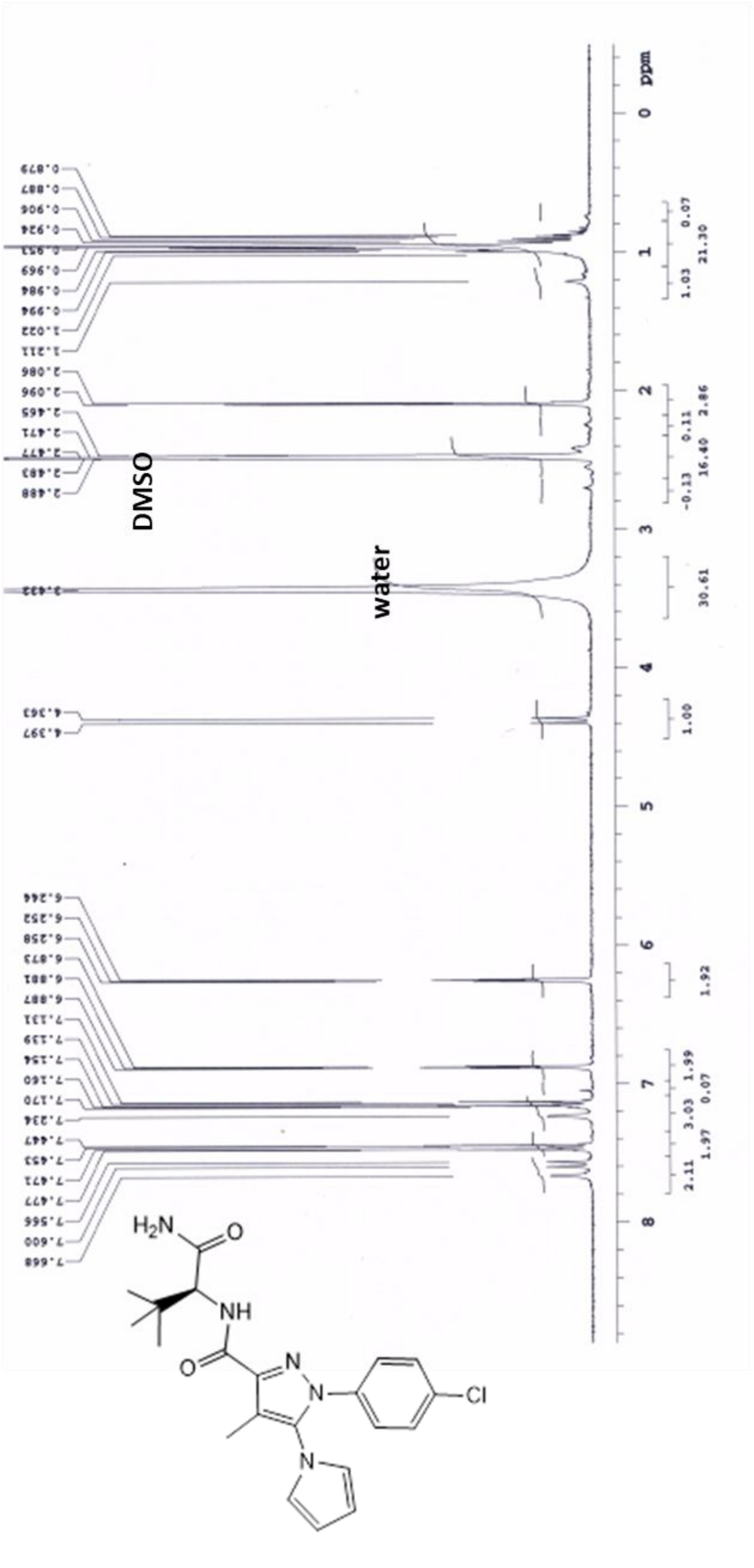

15

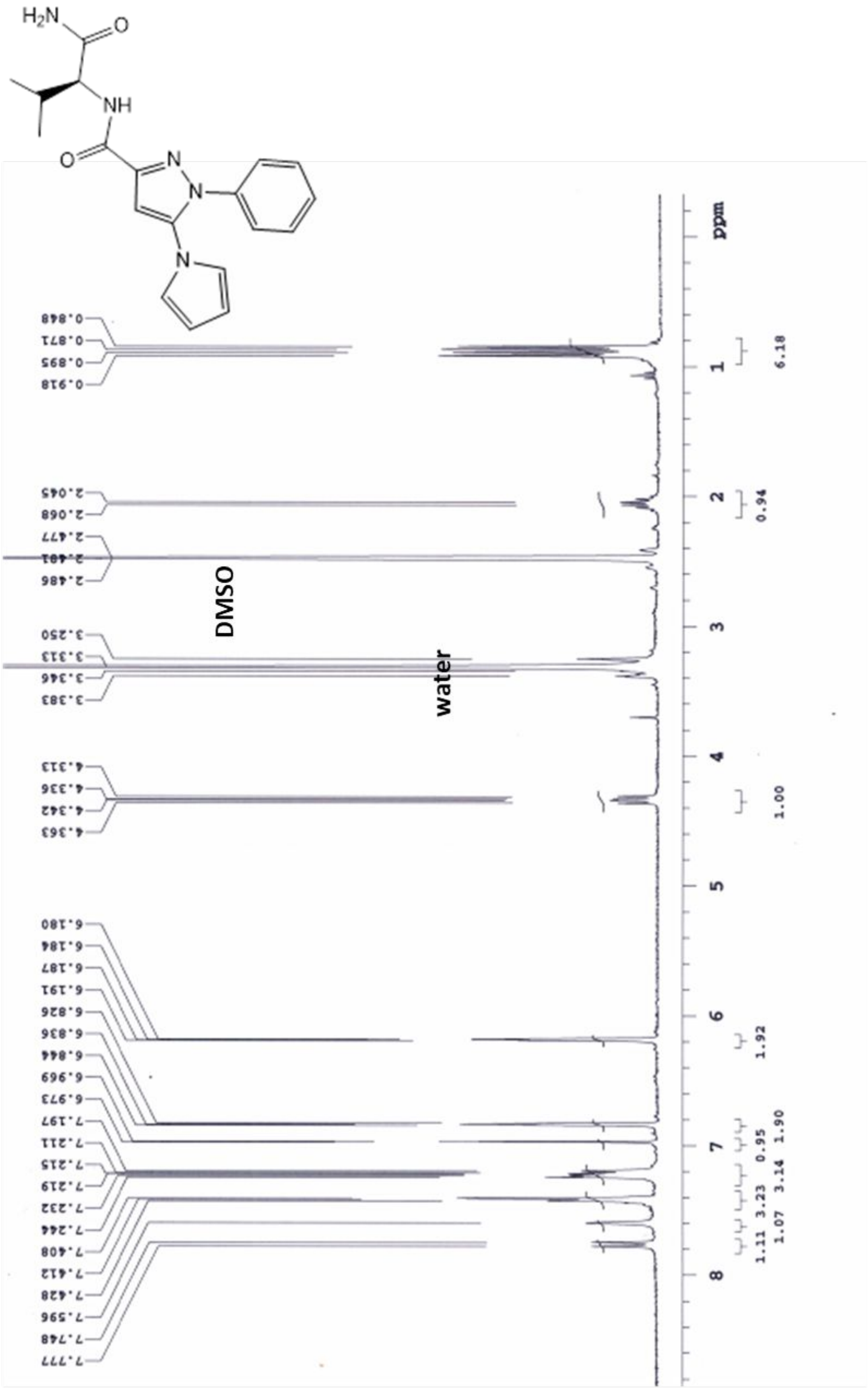

16

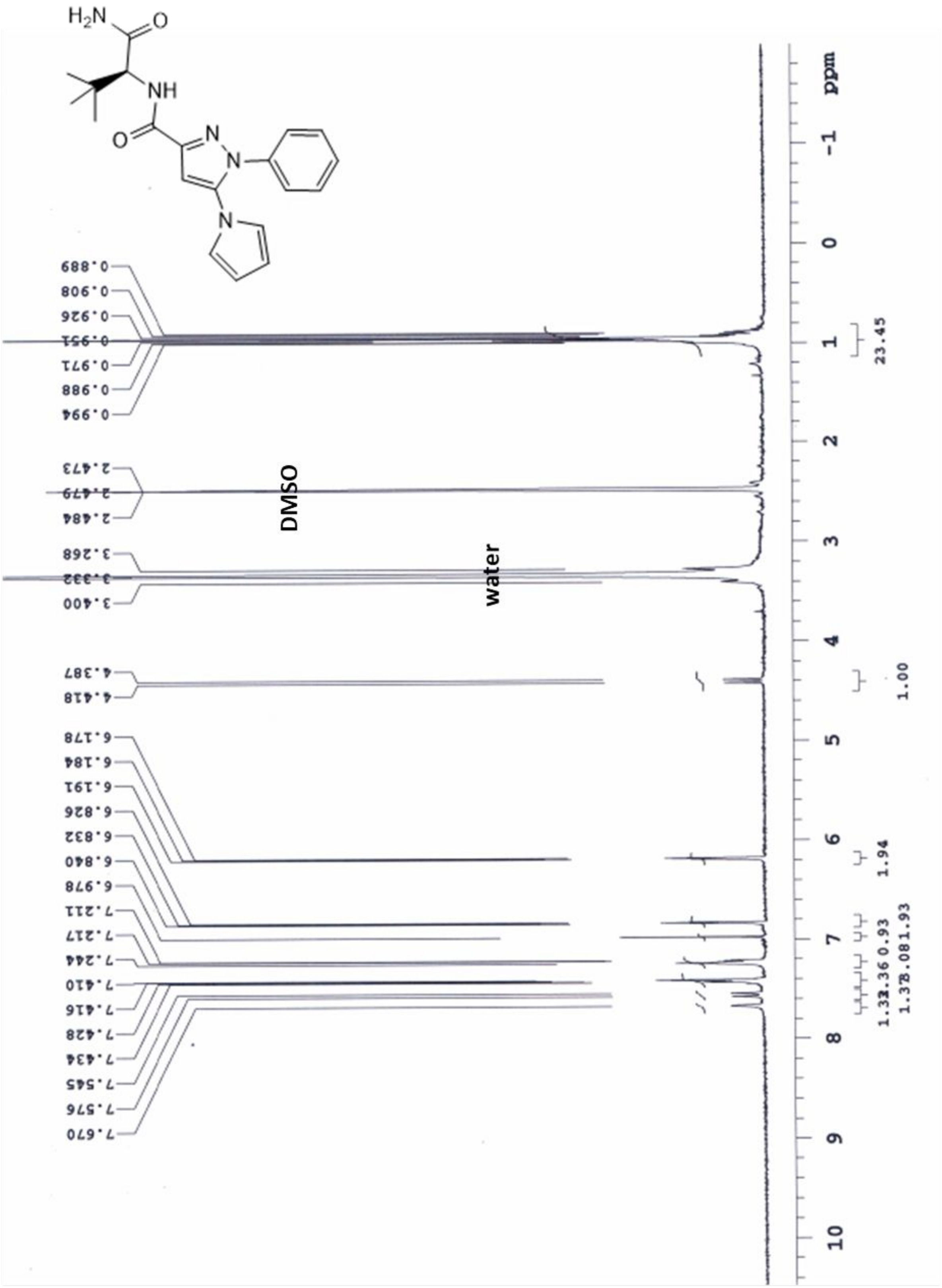
H2N
O
NH
O
N
N
N
0.889
0.908
0.926
0.951
0.971
0.988
0.994
2.473
2.479
2.484
DMSO
3.268
3.332
3.400
water
4.387
4.418
6.178
6.184
6.191
6.826
6.832
6.840
6.978
7.211
7.217
7.244
7.410
7.416
7.428
7.434
7.545
7.576
7.670
23.45
1.00
1.94
ppm

17

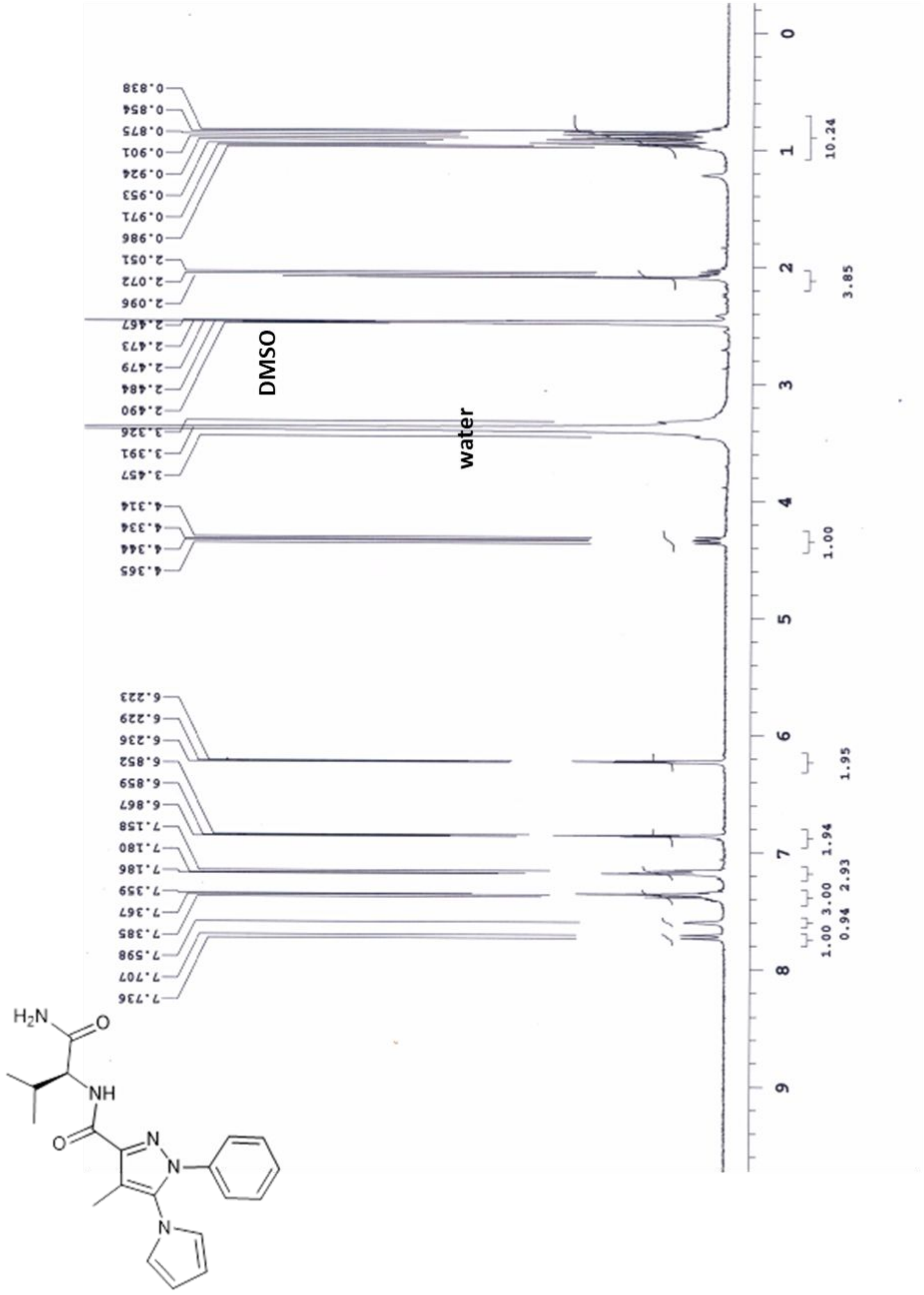

18

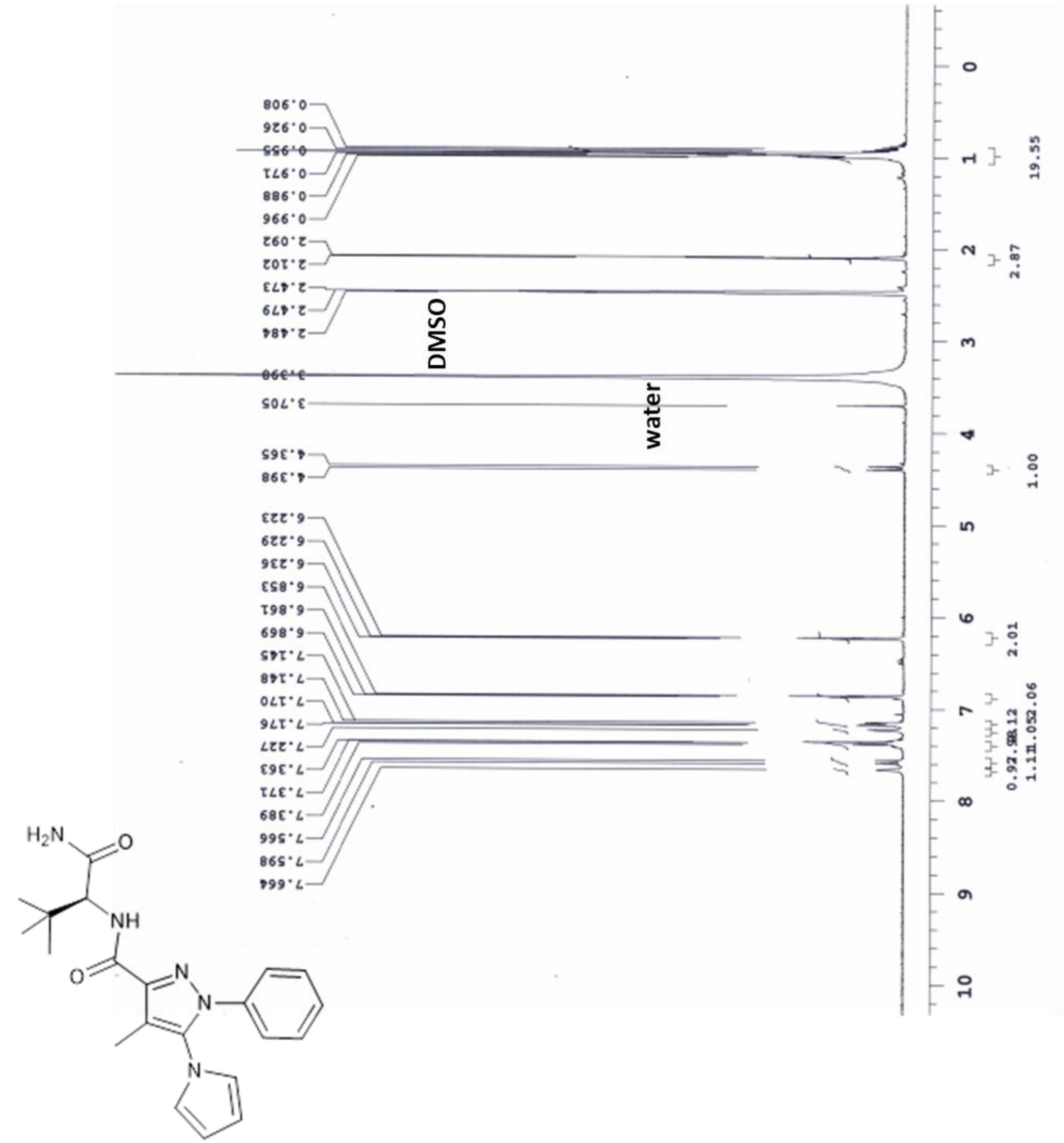

19

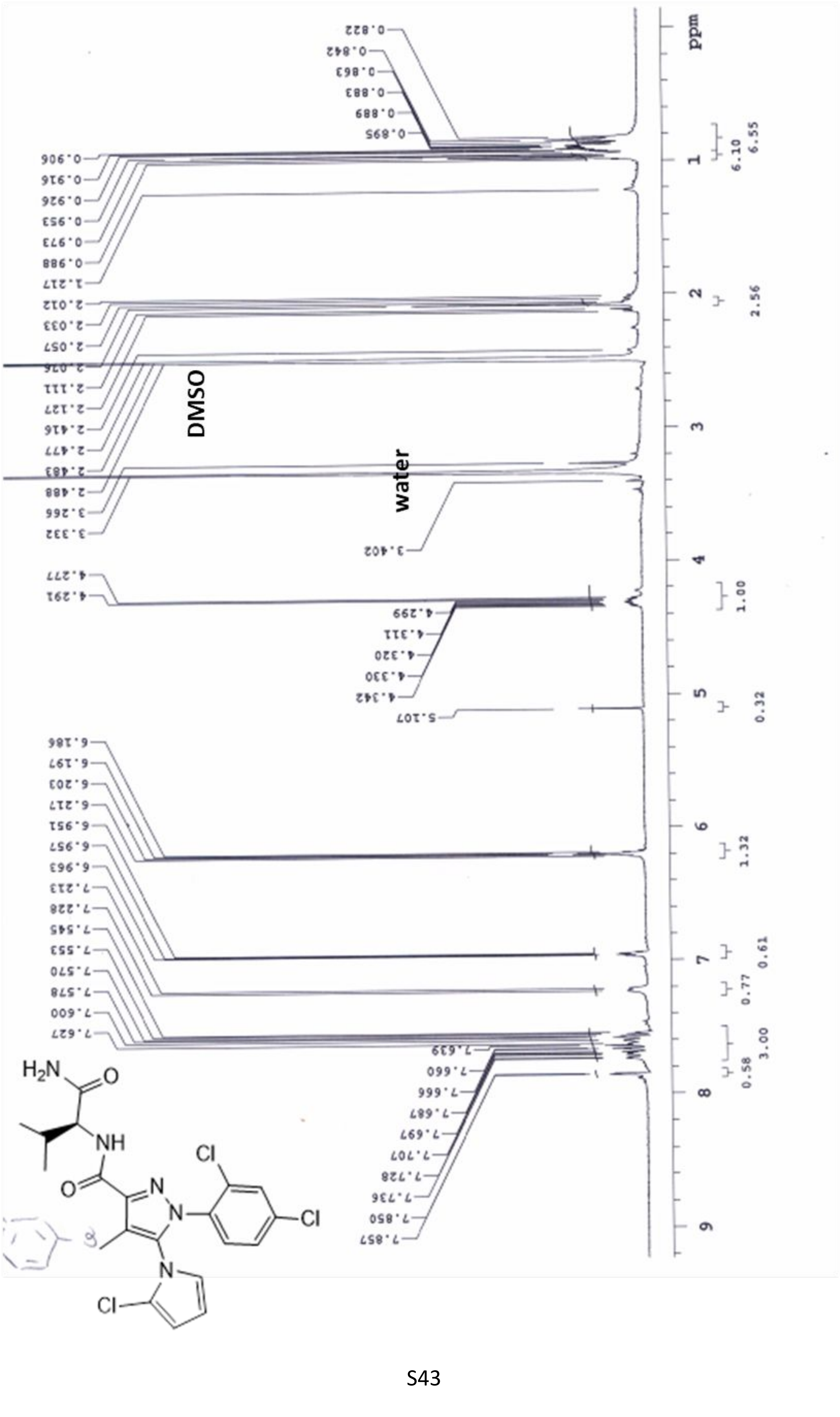

20

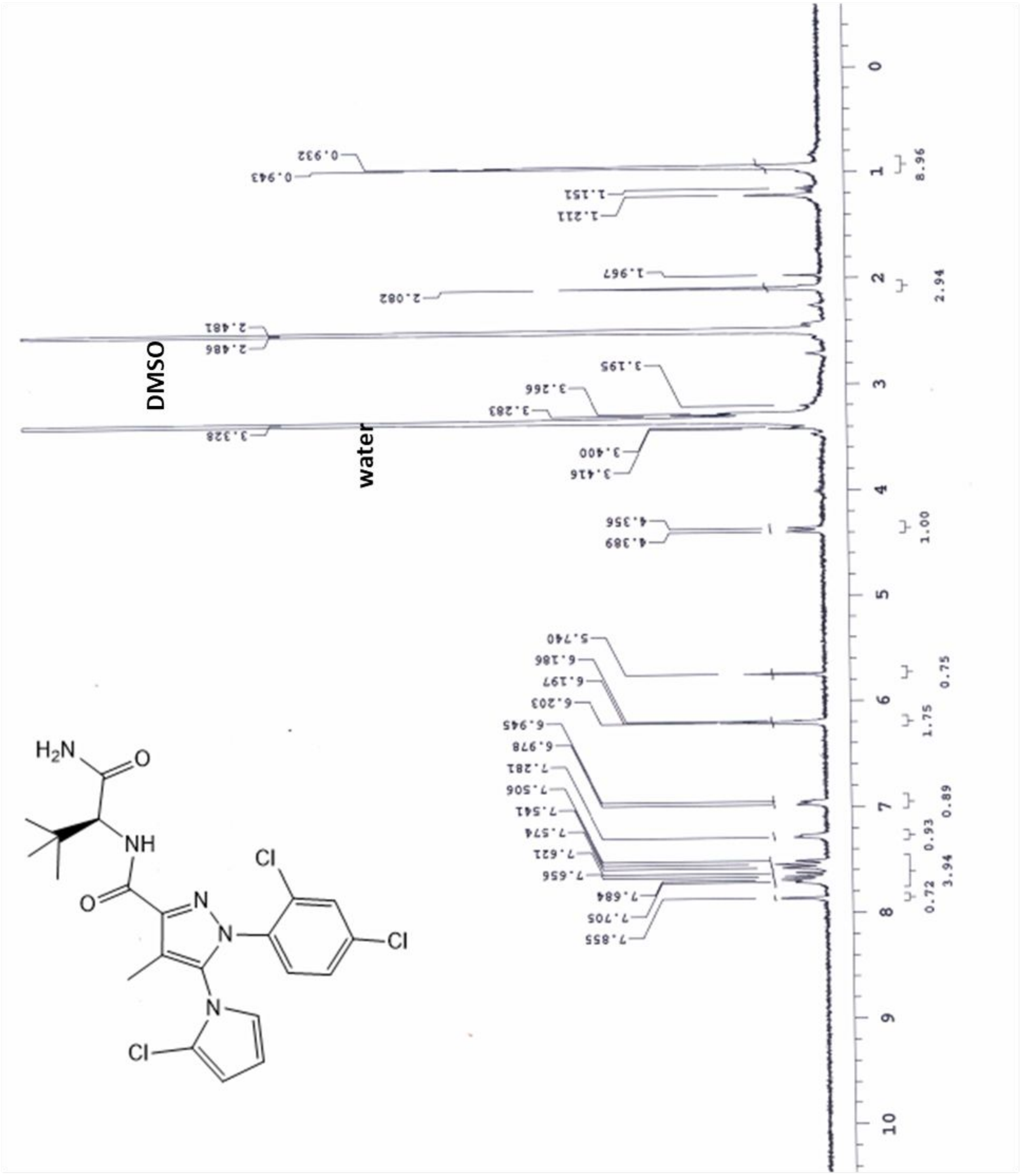

21

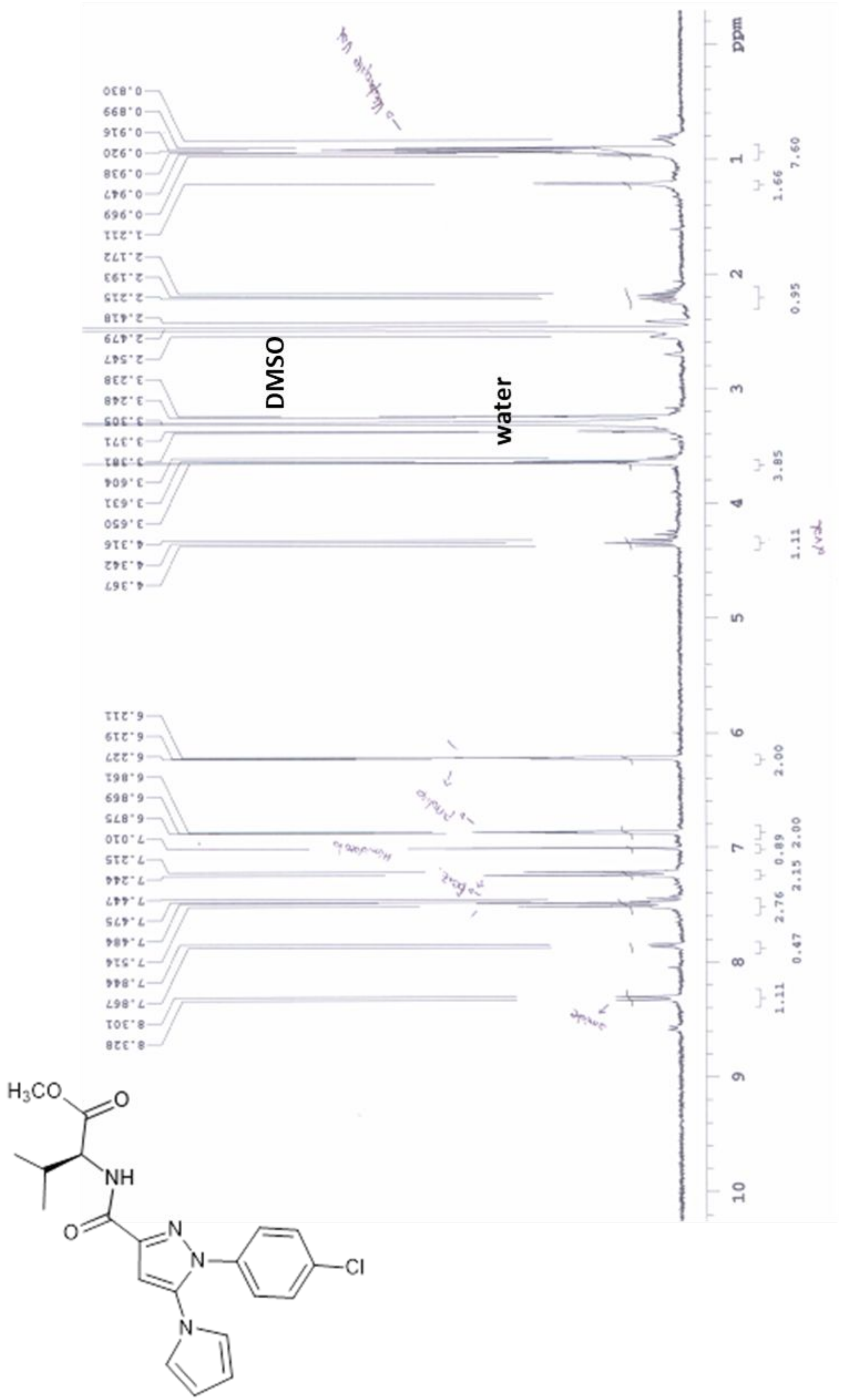

22

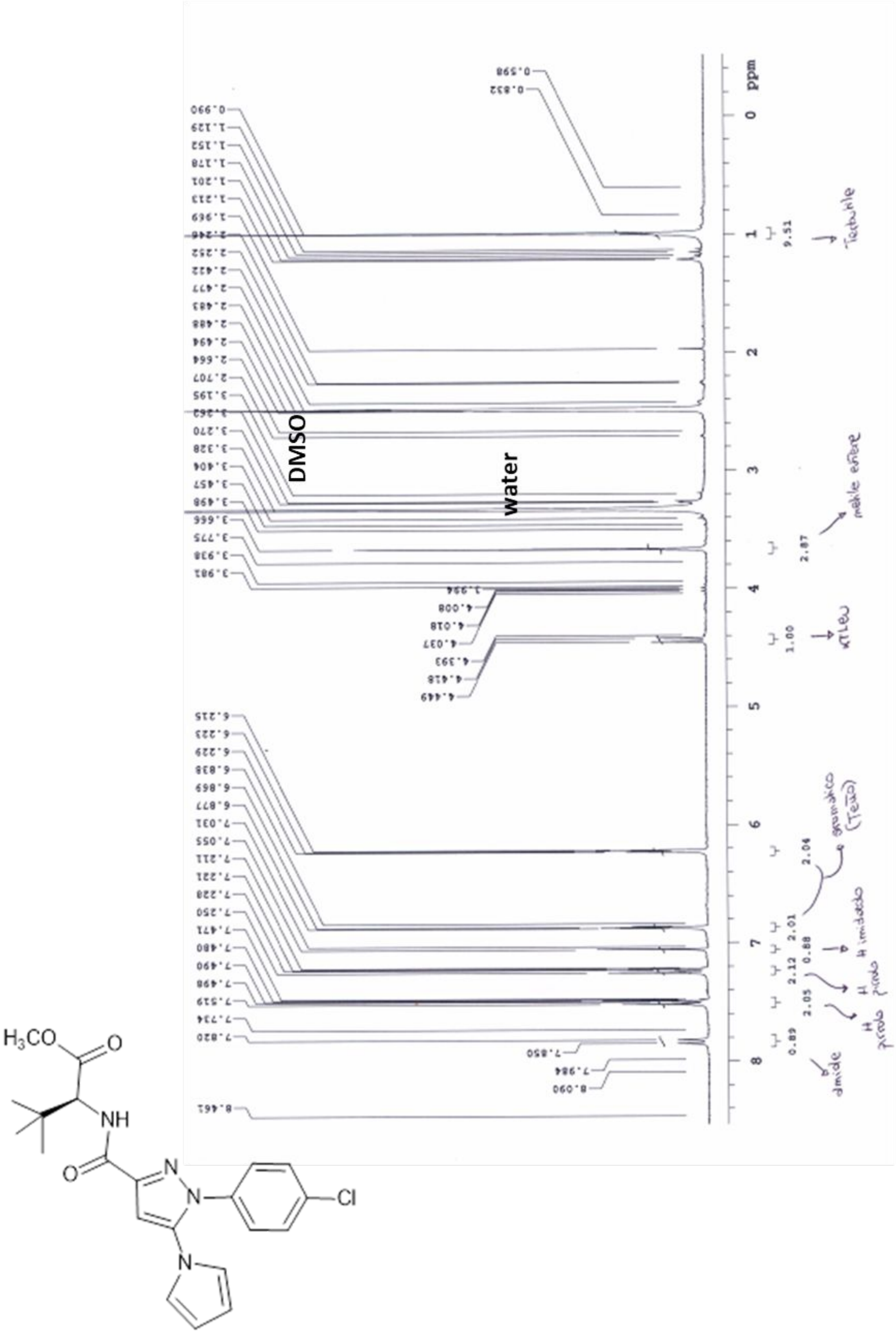

23

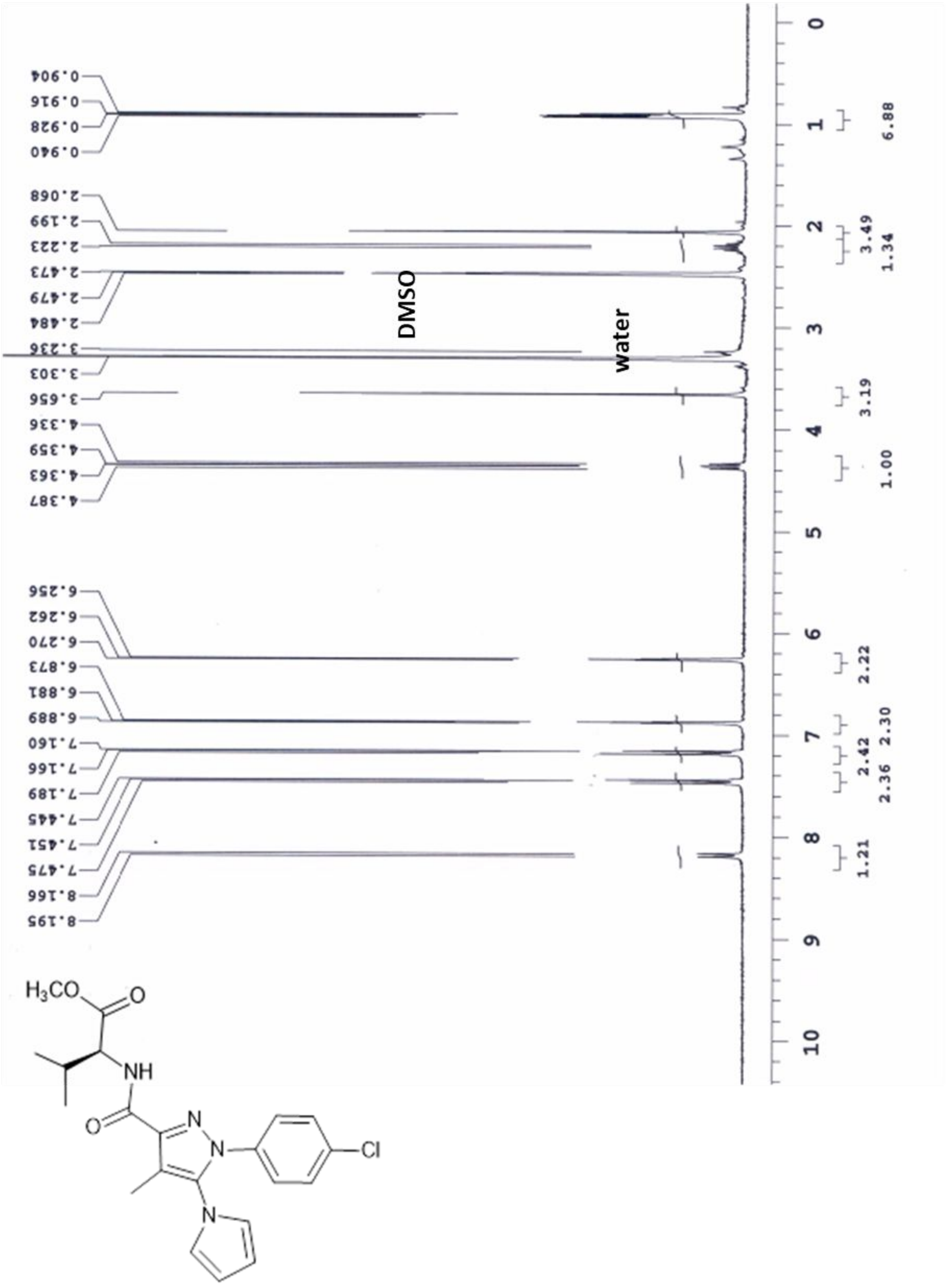

24

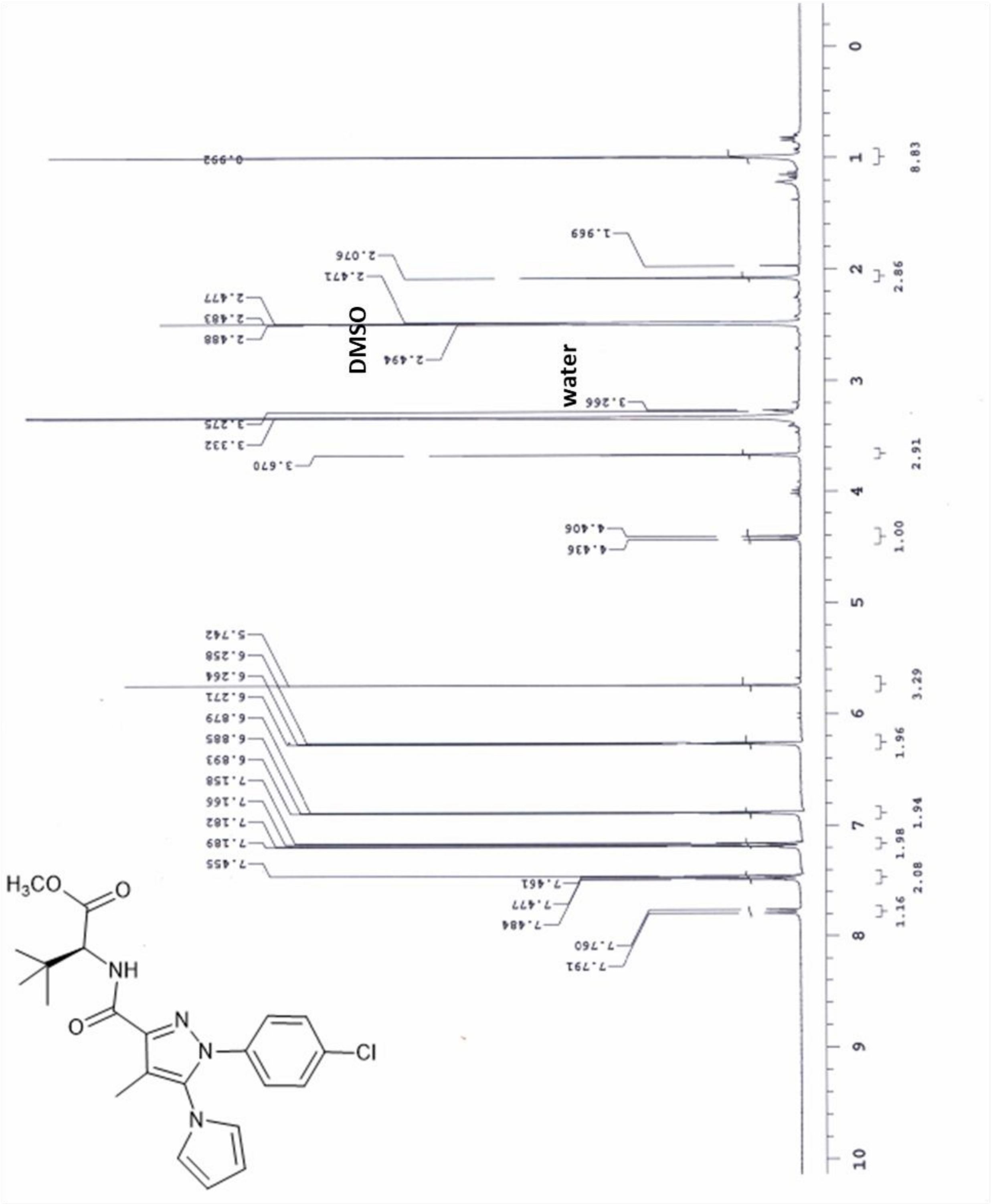

25

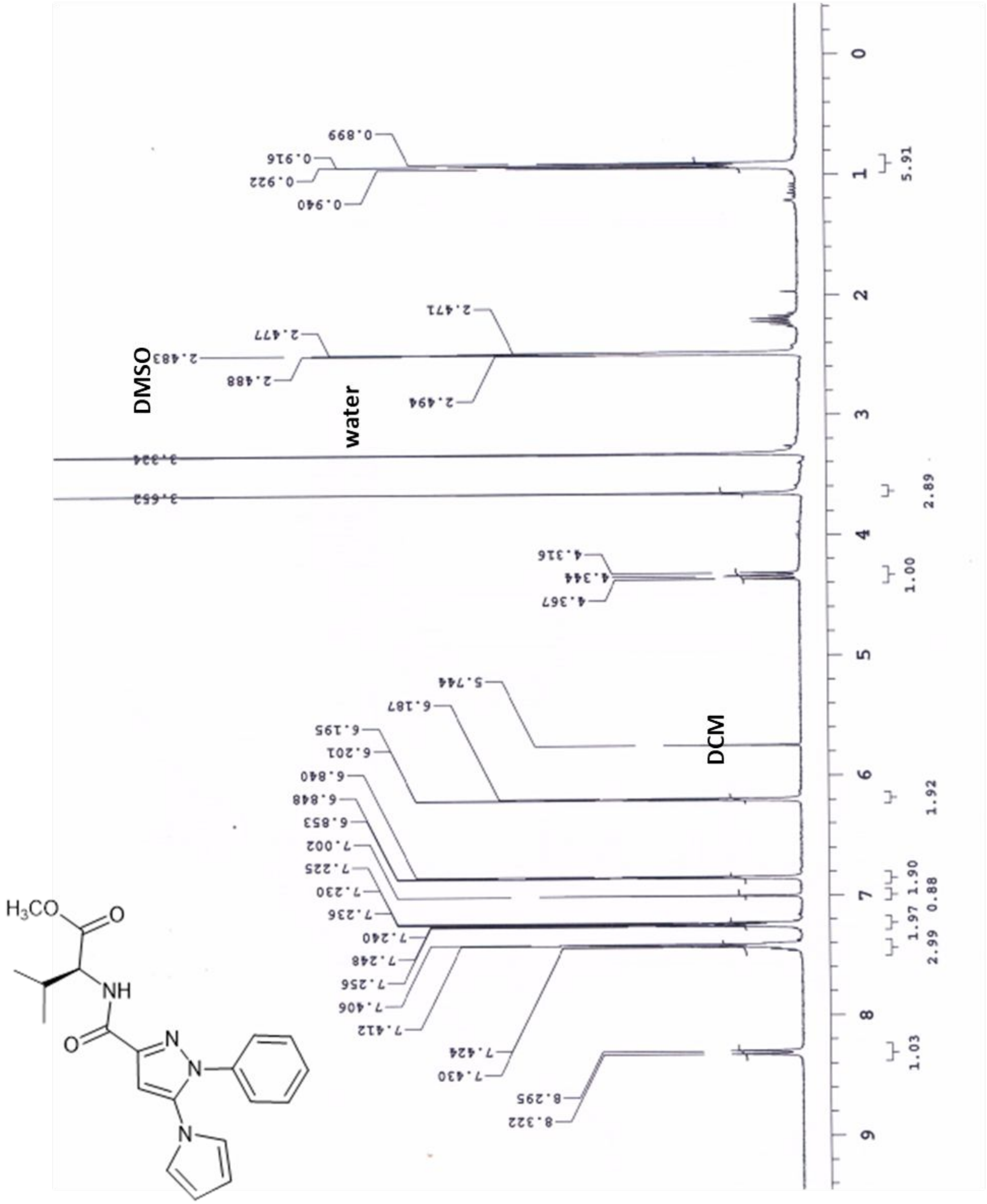

26

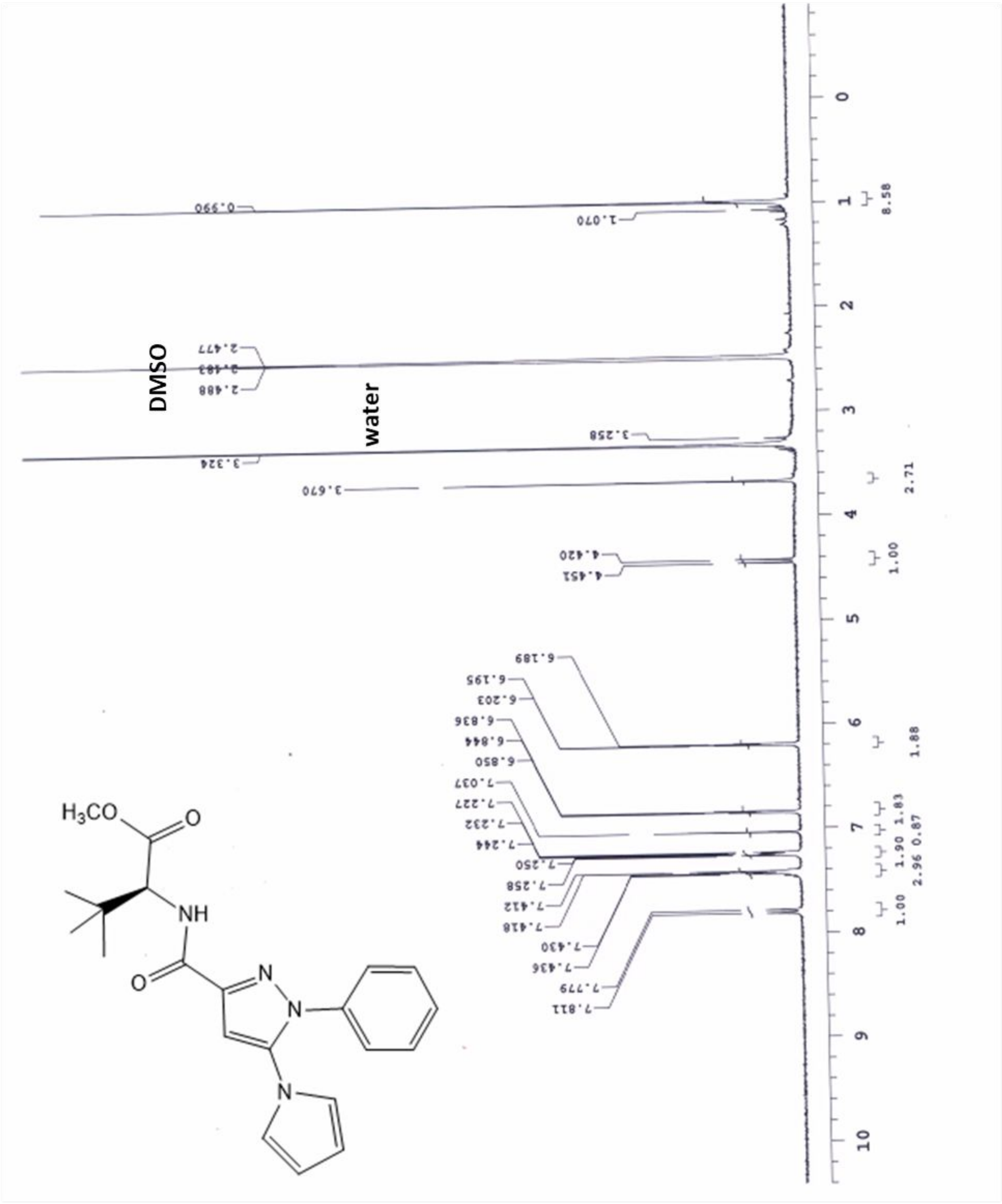

27

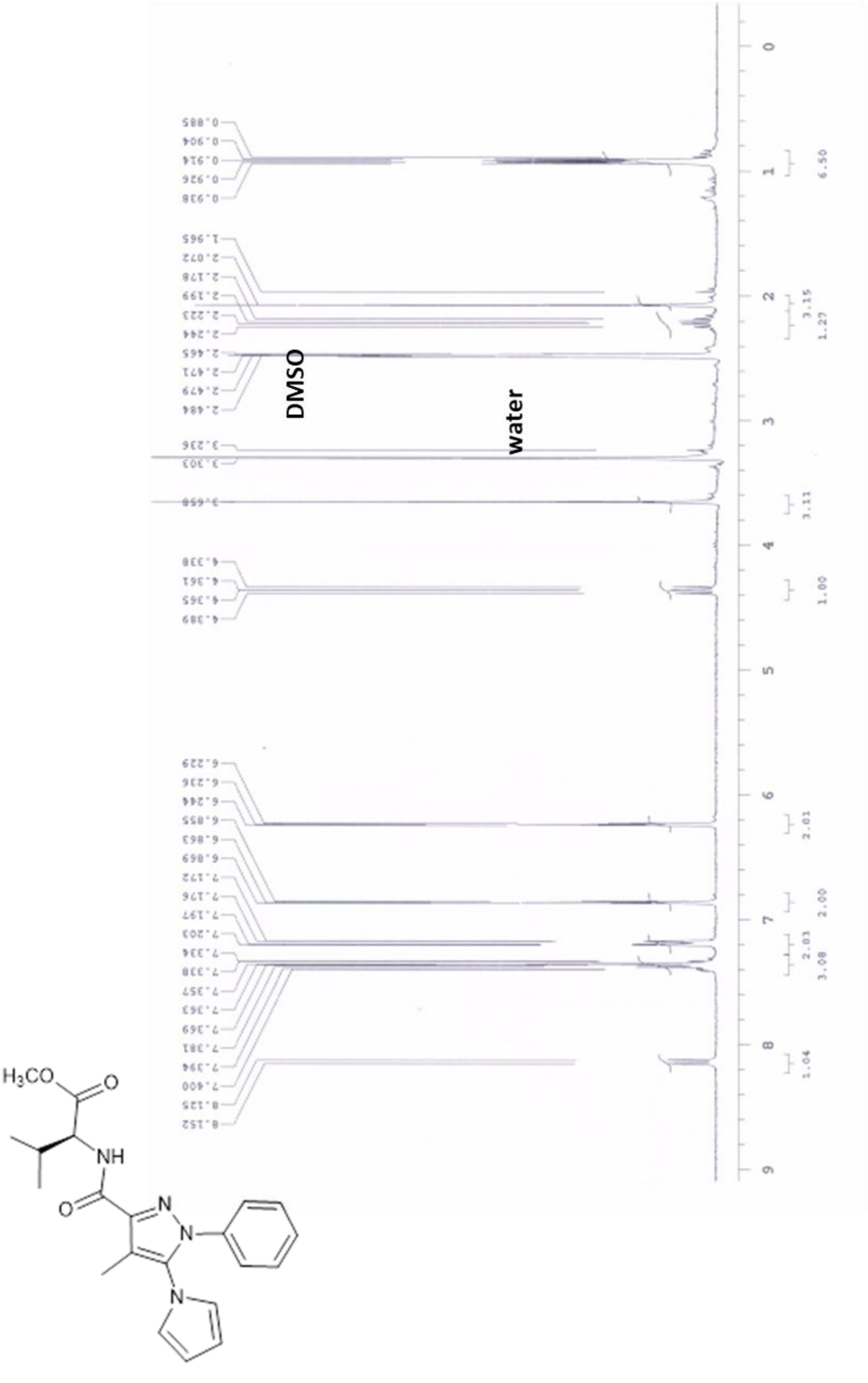

28

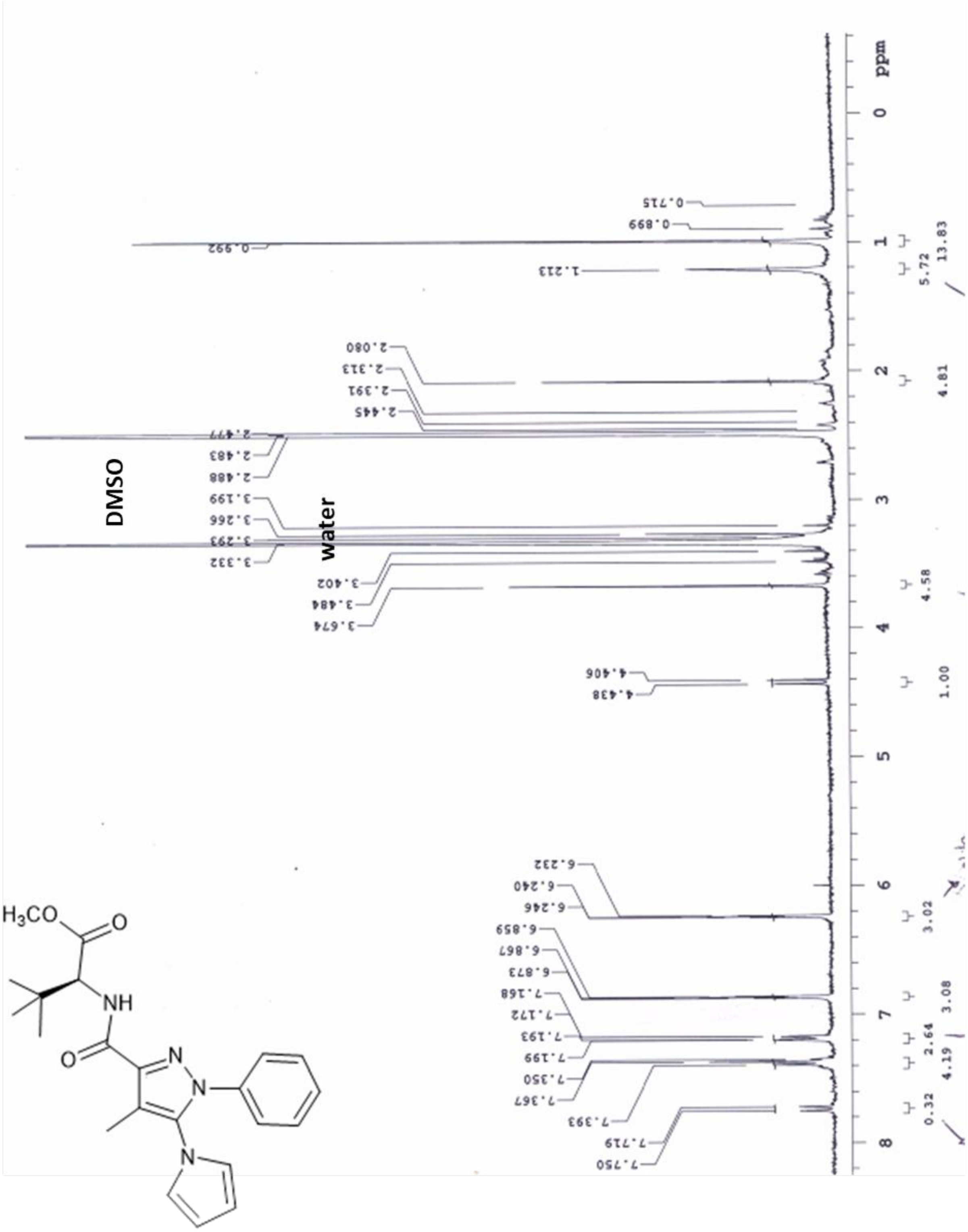

29

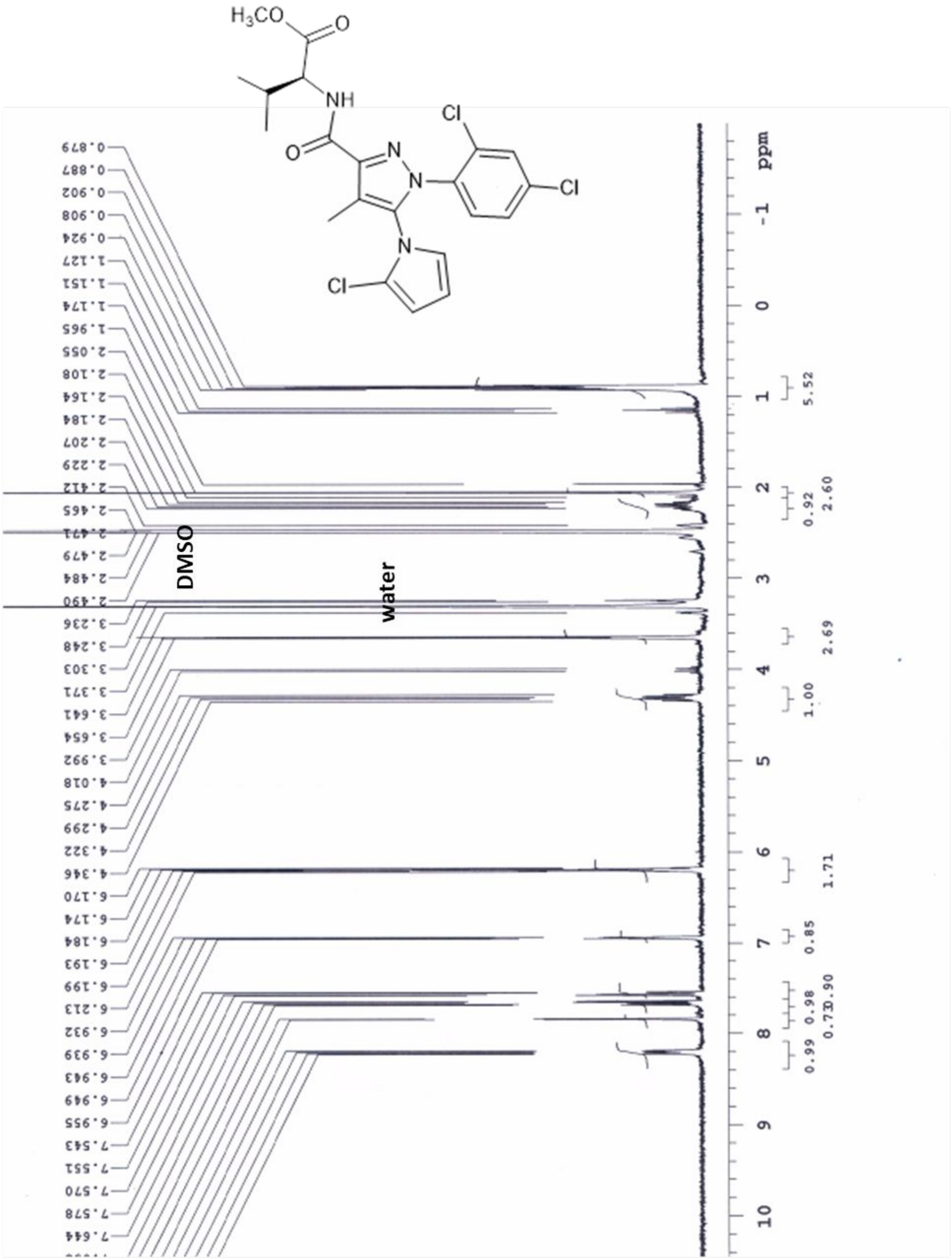

30

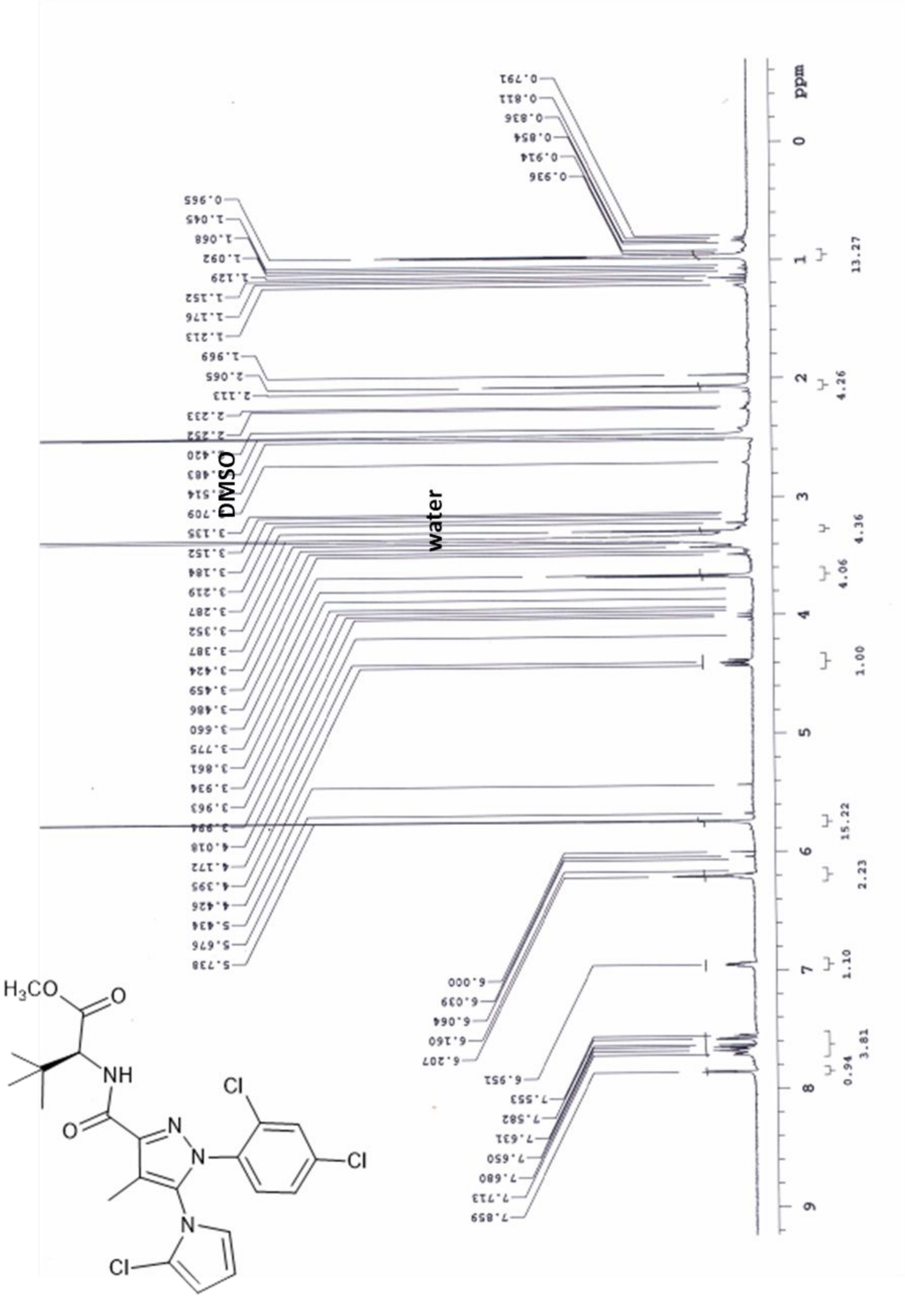

31

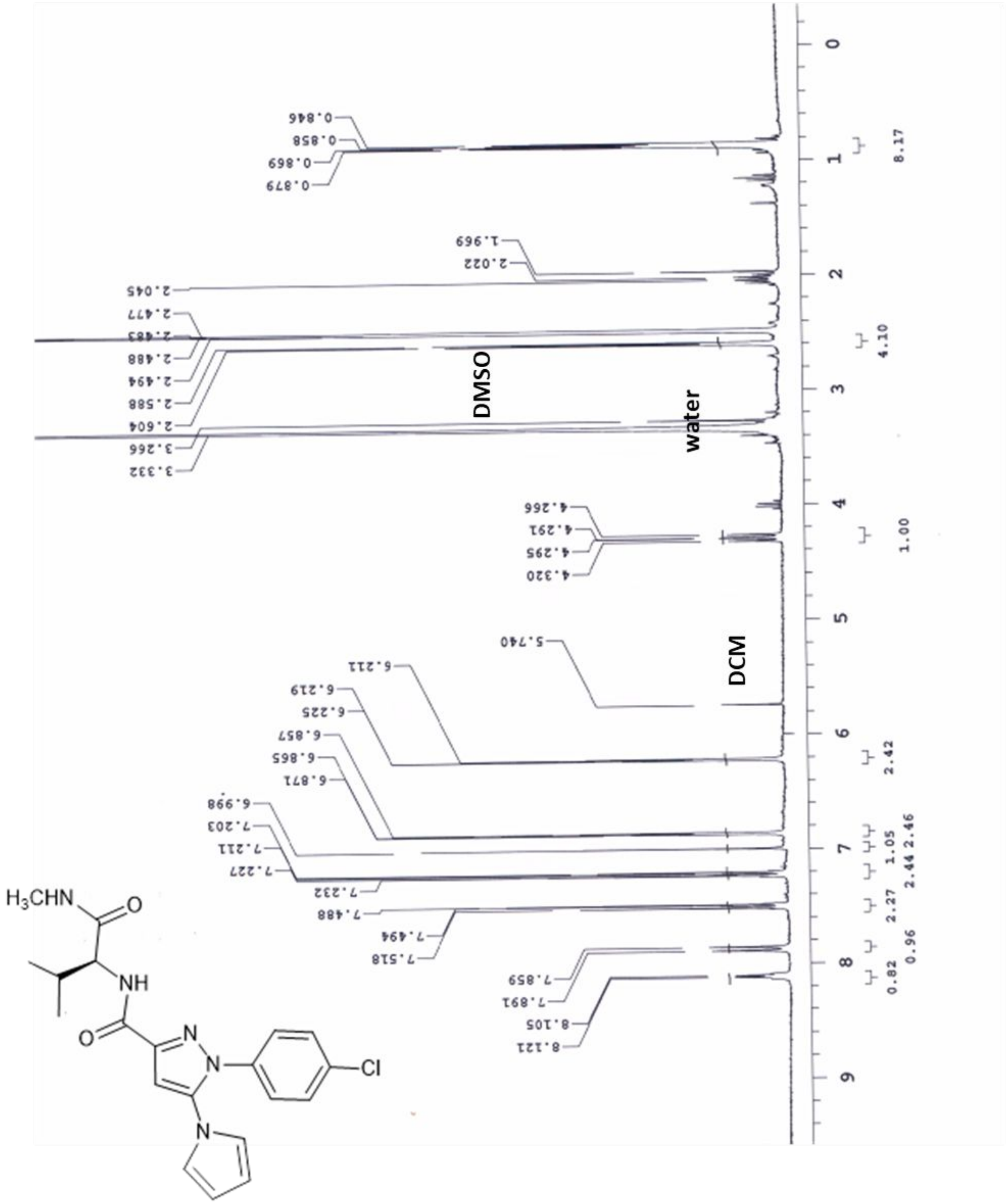

32

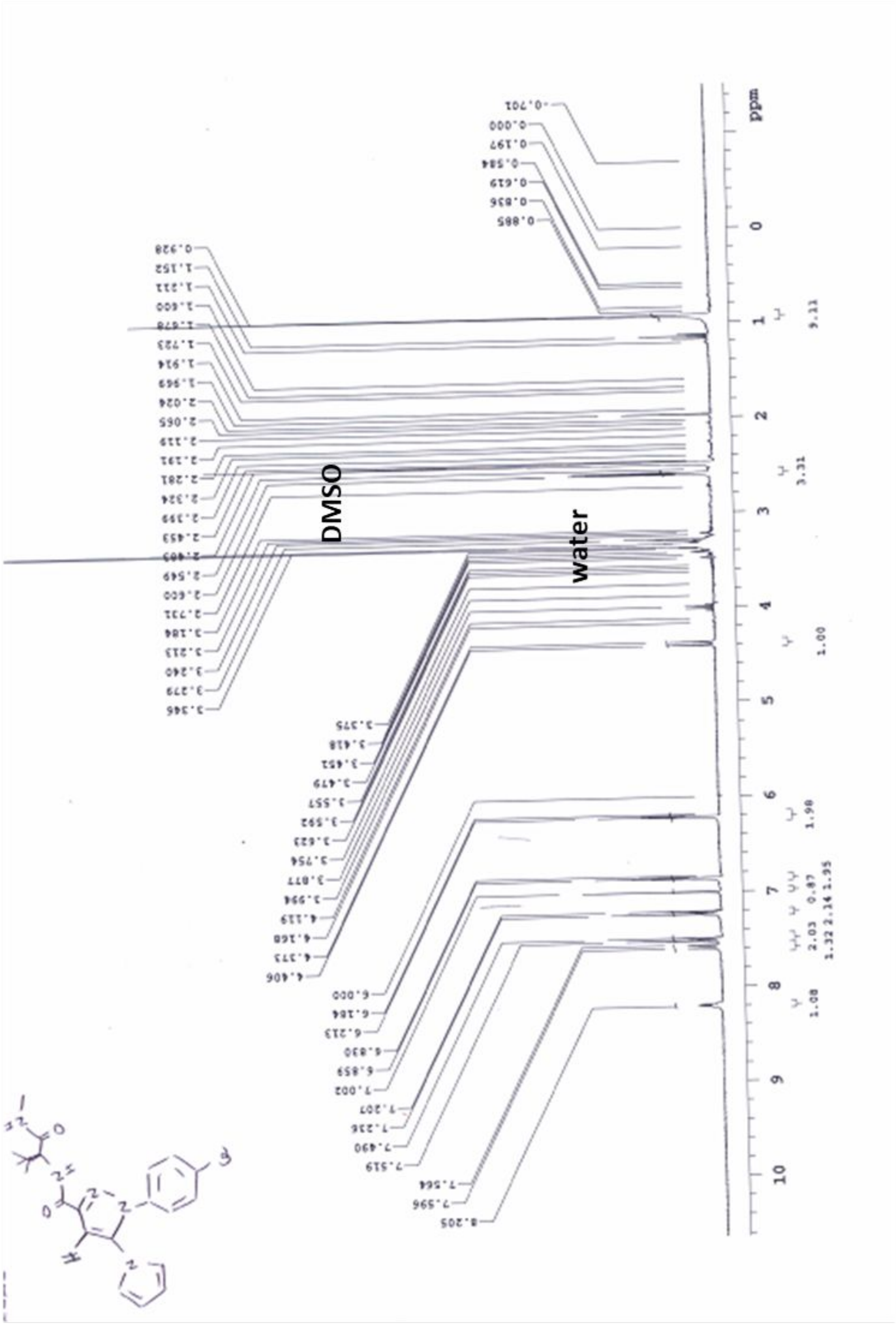

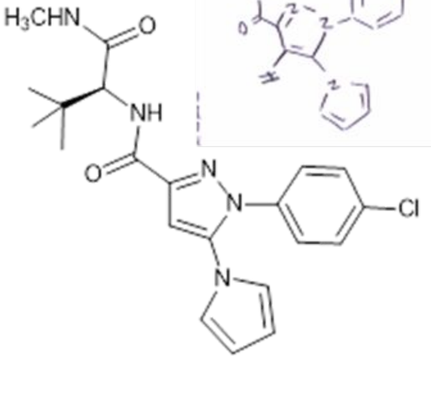

33

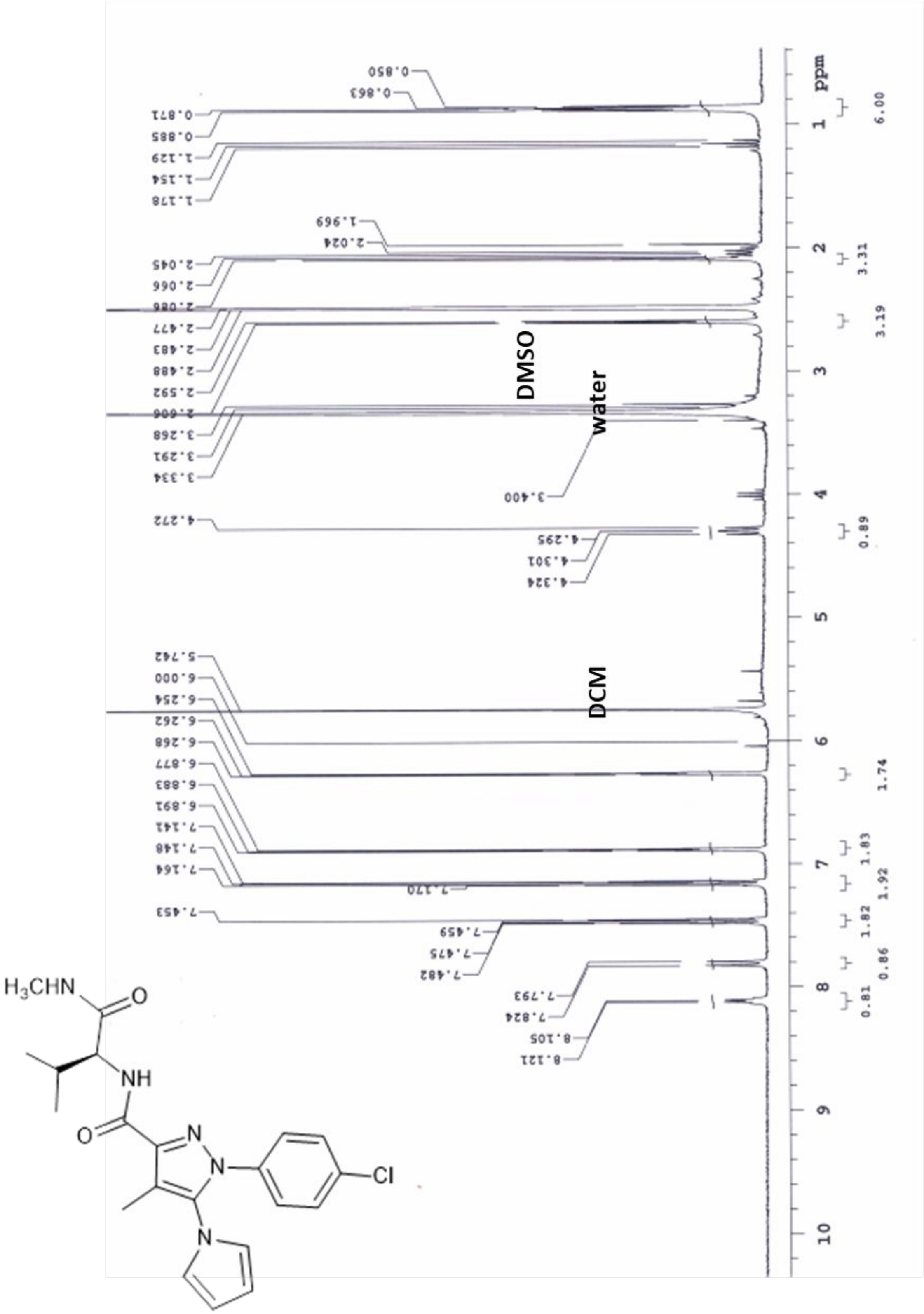

0.850
0.863
0.871
0.885
1.129
1.154
1.178
1.969
2.024
2.045
2.066
2.086
2.477
2.483
2.488
2.592
2.606
3.268
3.291
3.334
3.400
4.272
4.295
4.301
4.324
5.742
6.000
6.254
6.262
6.268
6.877
6.883
6.891
7.141
7.148
7.164
7.170
7.453
7.459
7.475
7.482
7.793
7.824
8.105
8.121
DMSO
water
DCM
ppm
6.00
3.31
3.19
0.89
1.74
1.83
1.92
1.82
0.86
0.81
H3CHN
O
NH
O
N
N
Cl

34

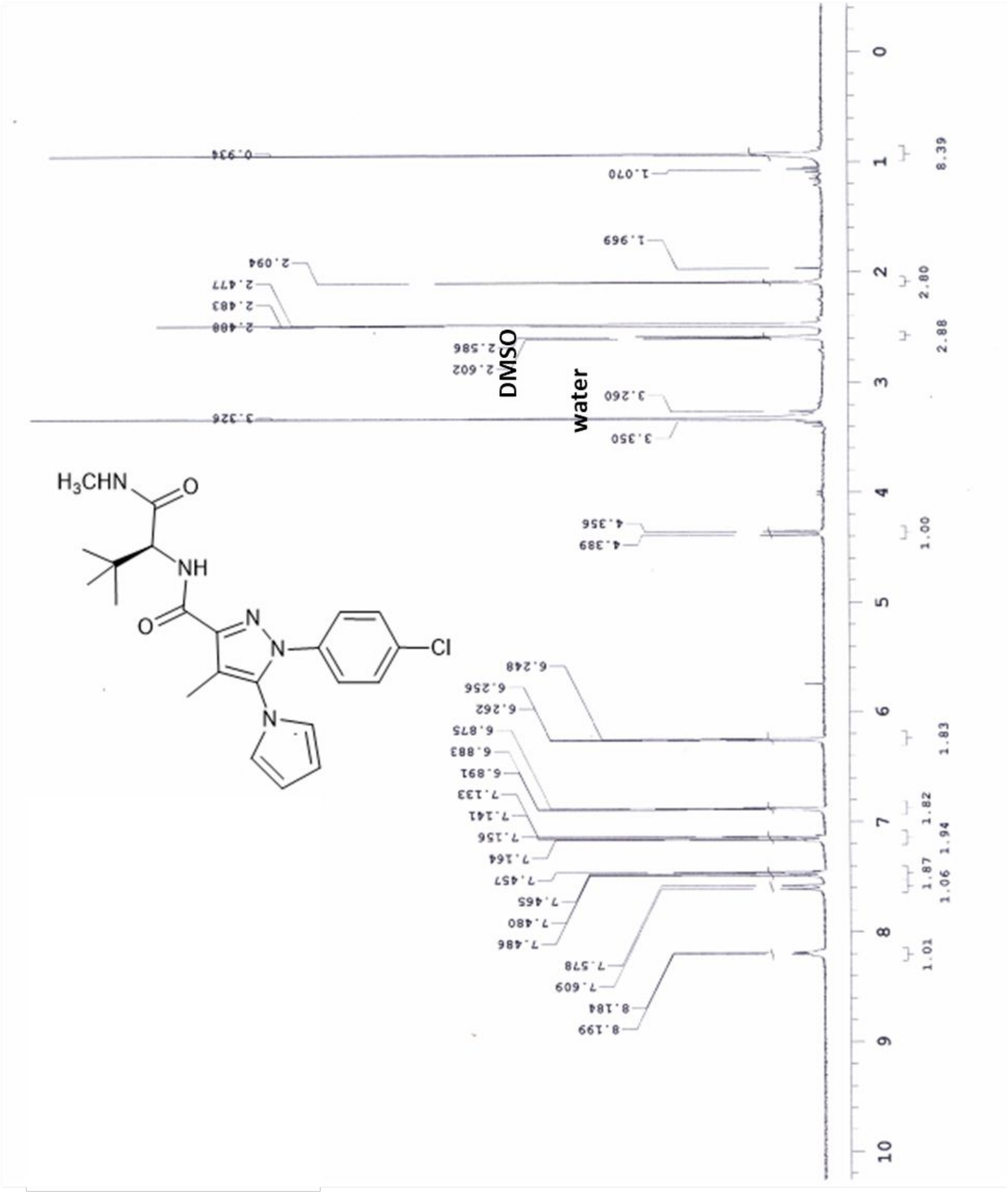

35

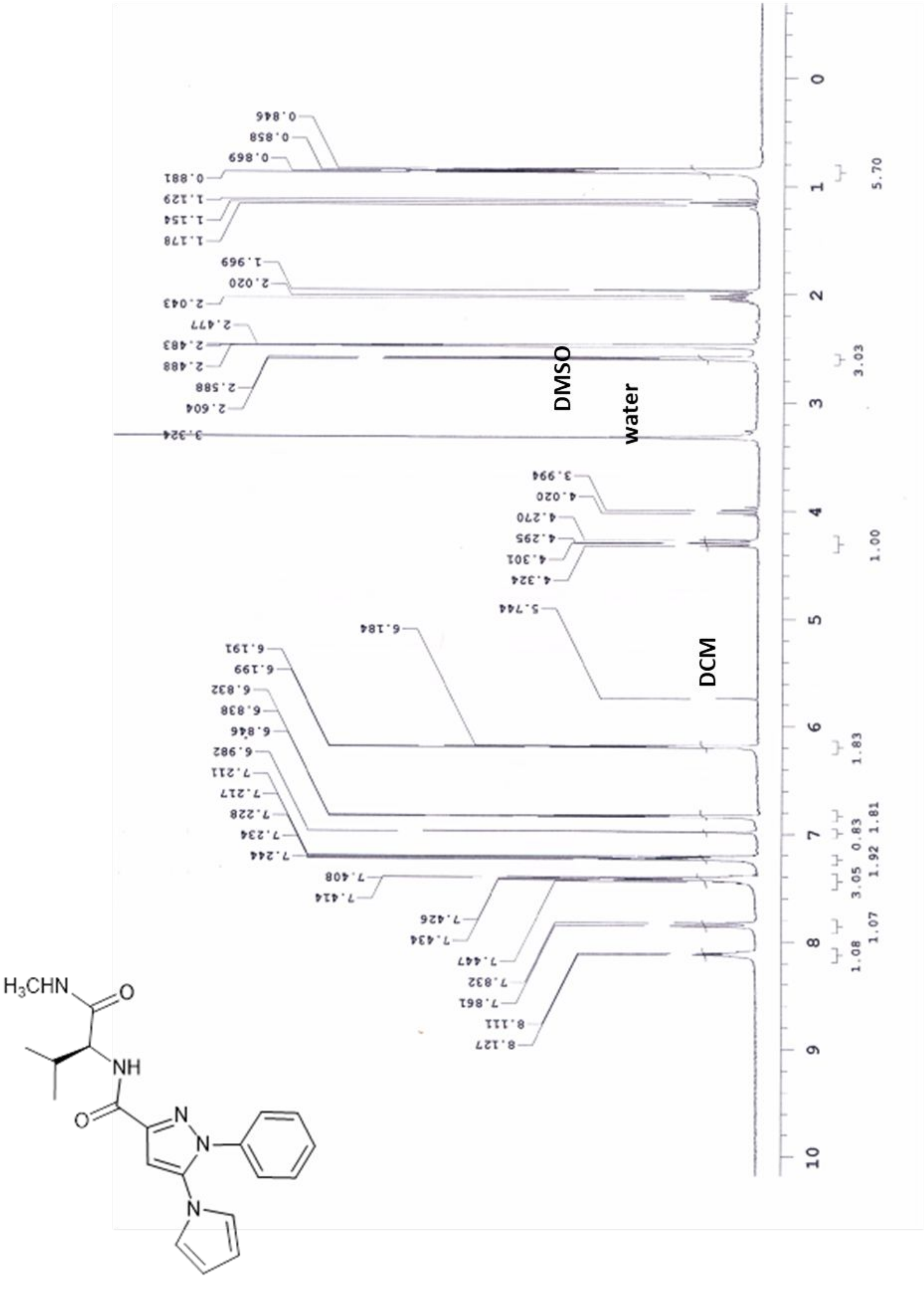

36

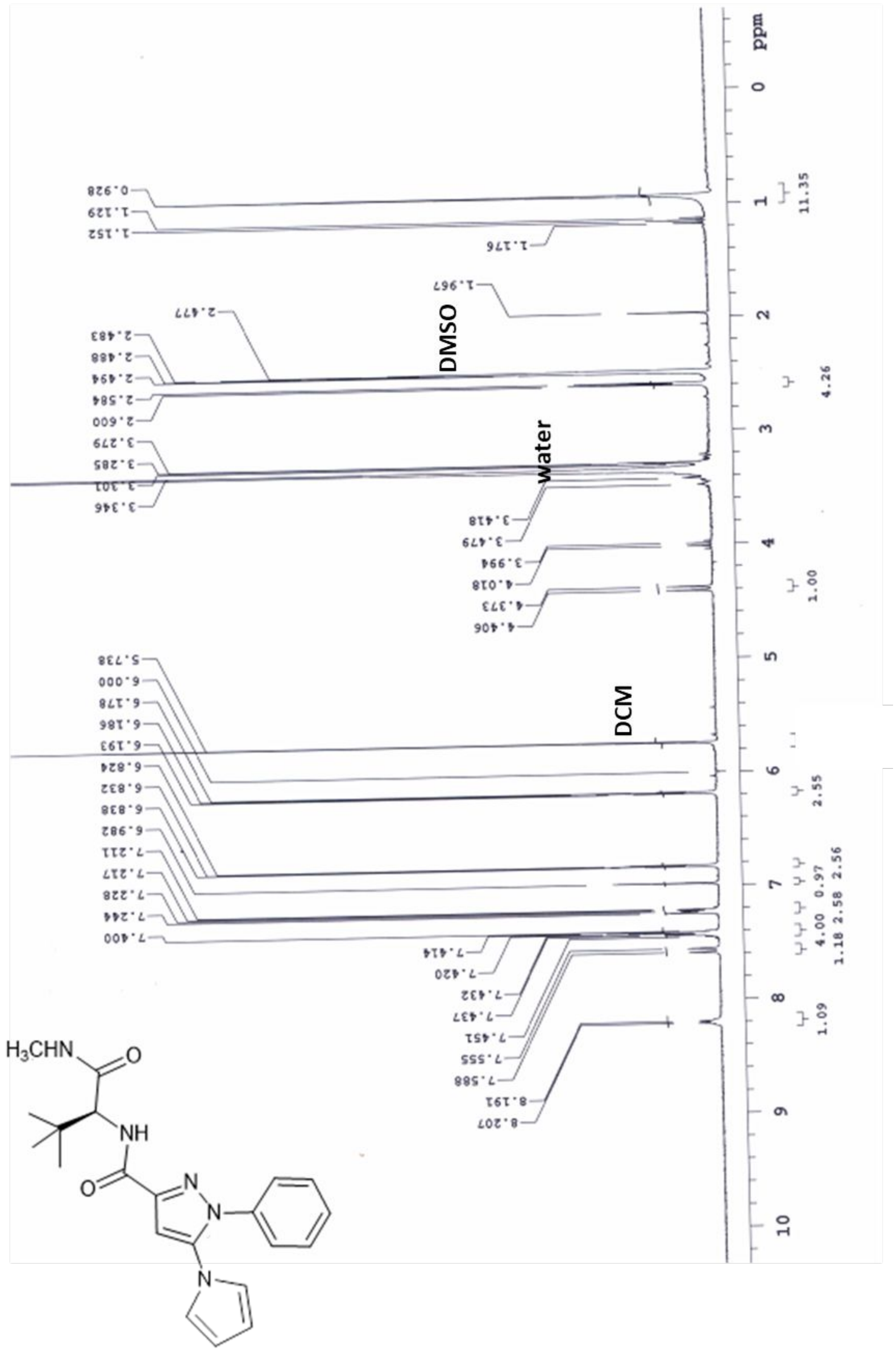

37

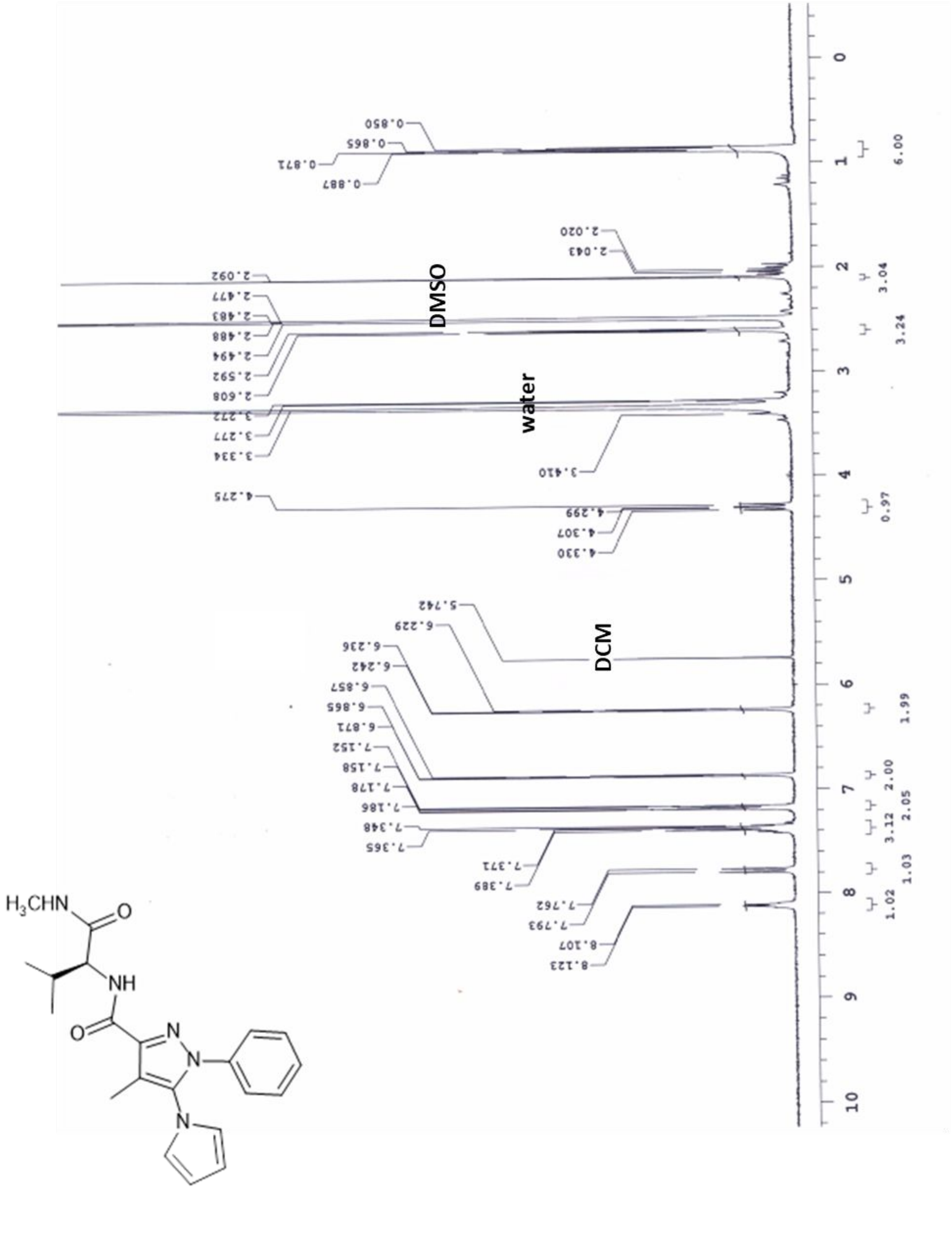

38

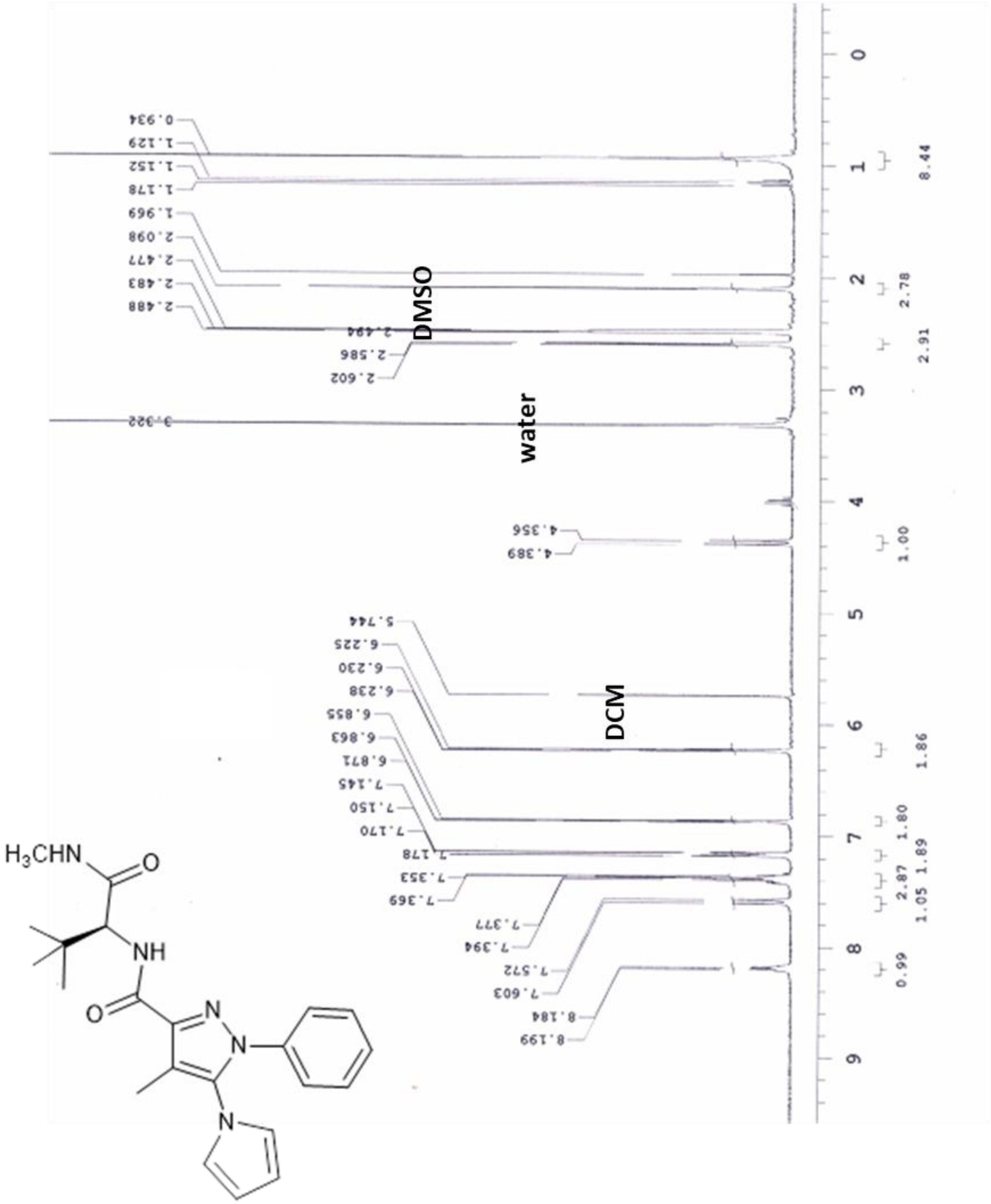

39

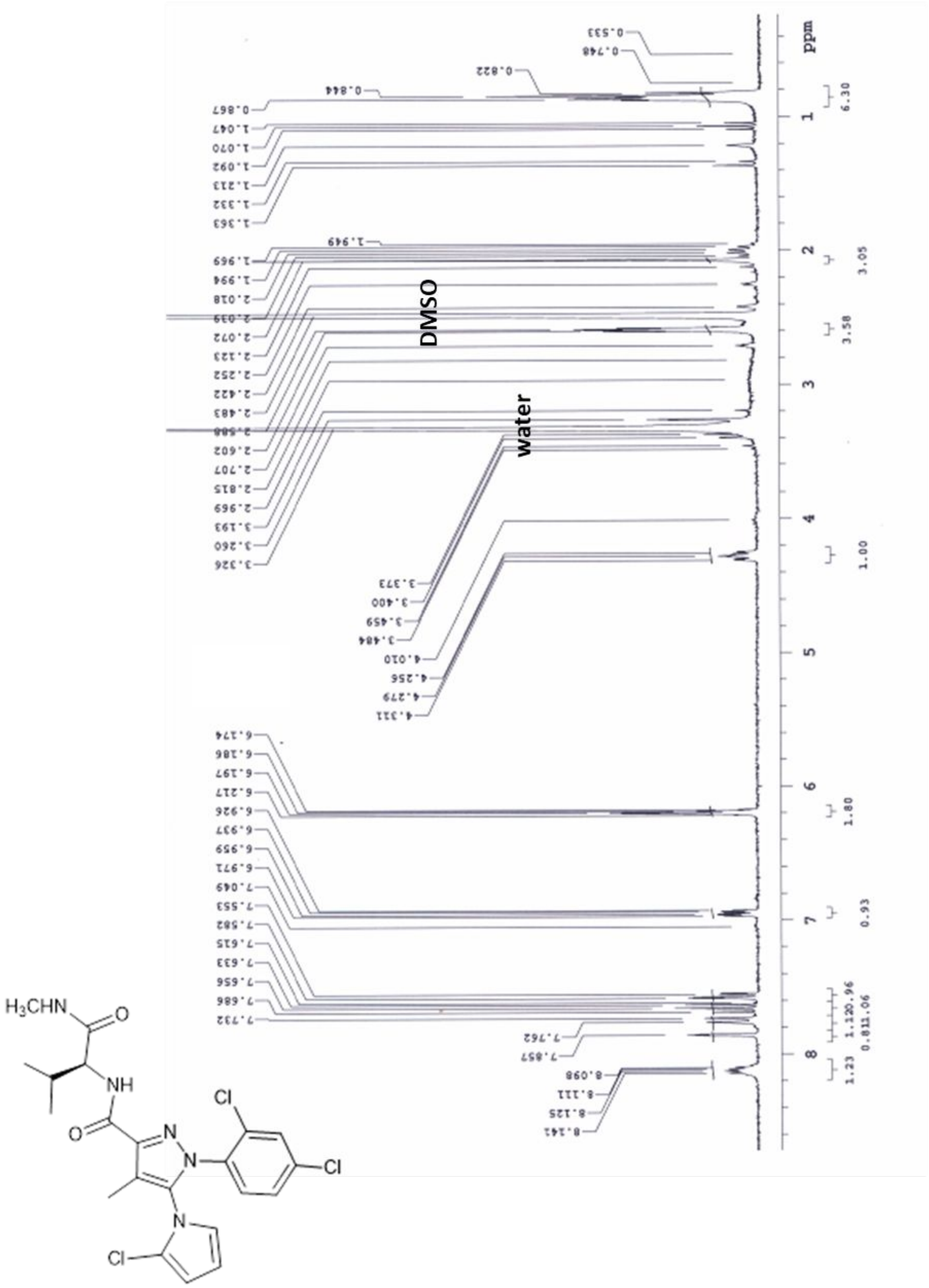

40

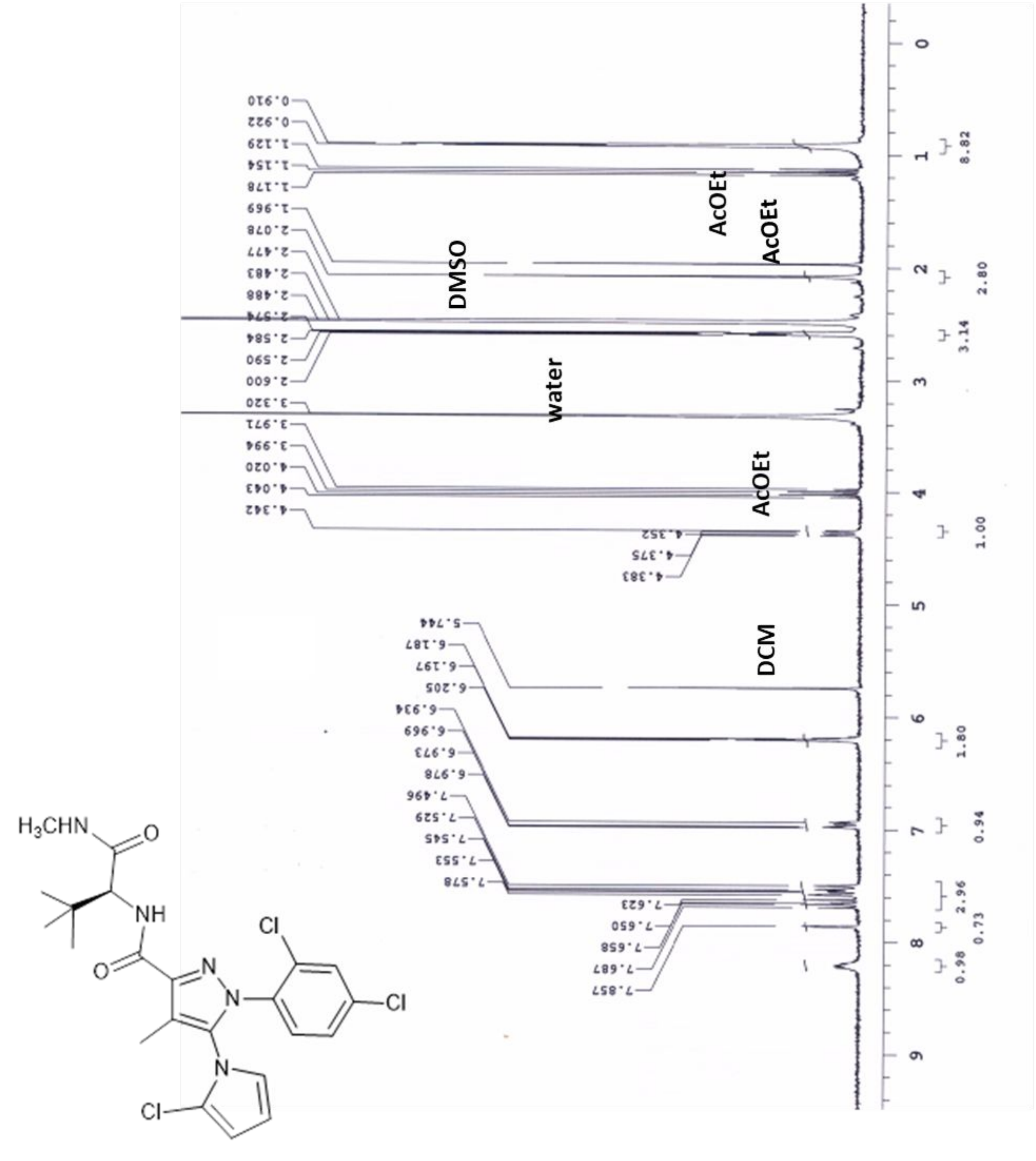

RP-HPLC TRACES

Table S4: Purity data for compounds **1-40.**

Compounds	% purity	r.t. (min)
**1**	98.61%	20.12
**2**	96.14%	20.36
**3**	97.69%	21.01
**4**	96.21%	21.10
**5**	98.76%	19.32
**6**	97.30%	19.74
**7**	96.31%	19.81
**8**	96.97%	20.62
**9**	95.72%	21.53
**10**	95.47%	21.82
**11**	98.50%	19.42
**12**	96.35%	19.75
**13**	98.29%	20.21
**14**	95.48%	20.92
**15**	>99%	18.30
**16**	96.49%	19.27
**17**	96.89%	19.80
**18**	96.43%	19.63
**19**	95.33%	24.33
**20**	99.25%	23.84
**21**	>99%	21.50
**22**	96.50%	24.96
**23**	>99%	22.01
**24**	>99%	26.07
**25**	>99%	22.89

**26**	97.85%	23.68
**27**	97.19%	24.20
**28**	95.21%	24.77
**29**	95.75%	22.53
**30**	97.86%	26.34
**31**	98.27%	21.98
**32**	> 99%	23.07
**33**	99.09%	23.09
**34**	>99%	24.09
**35**	>99%	20.77
**36**	>99%	21.61
**37**	>99%	21.59
**38**	>99%	22.62
**39**	98.44%	23.91
**40**	99.16%	24.80

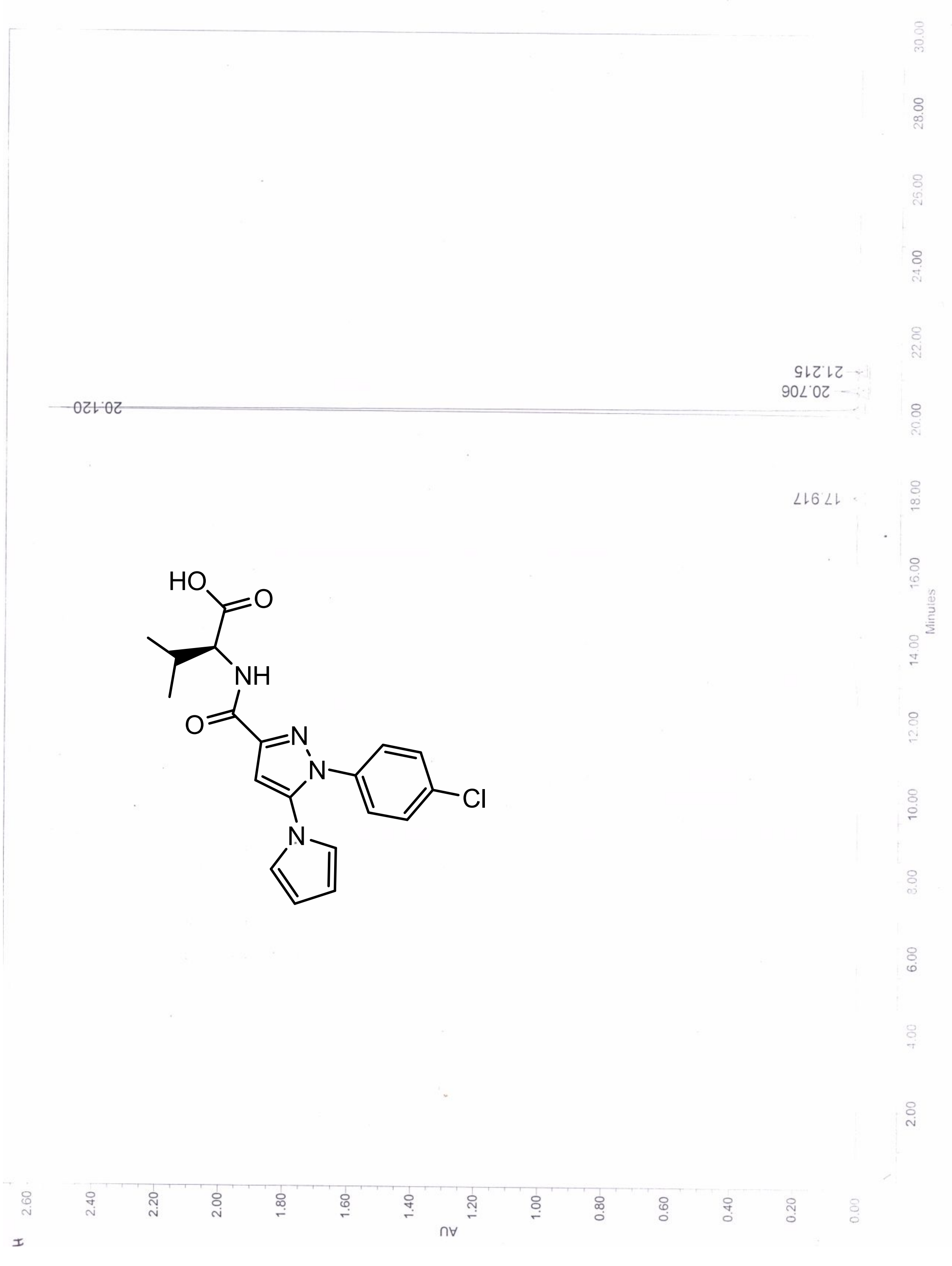

2

3

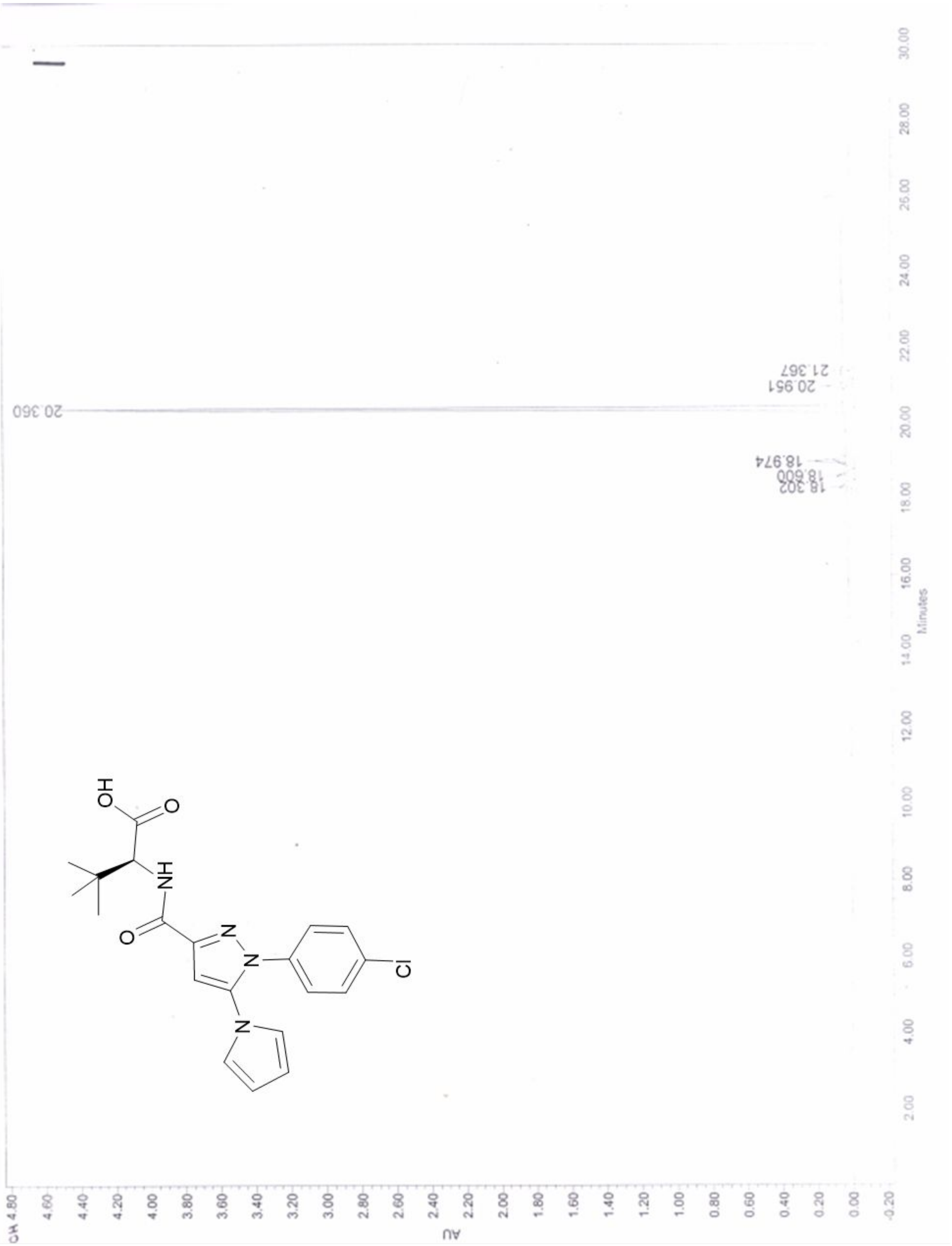

3

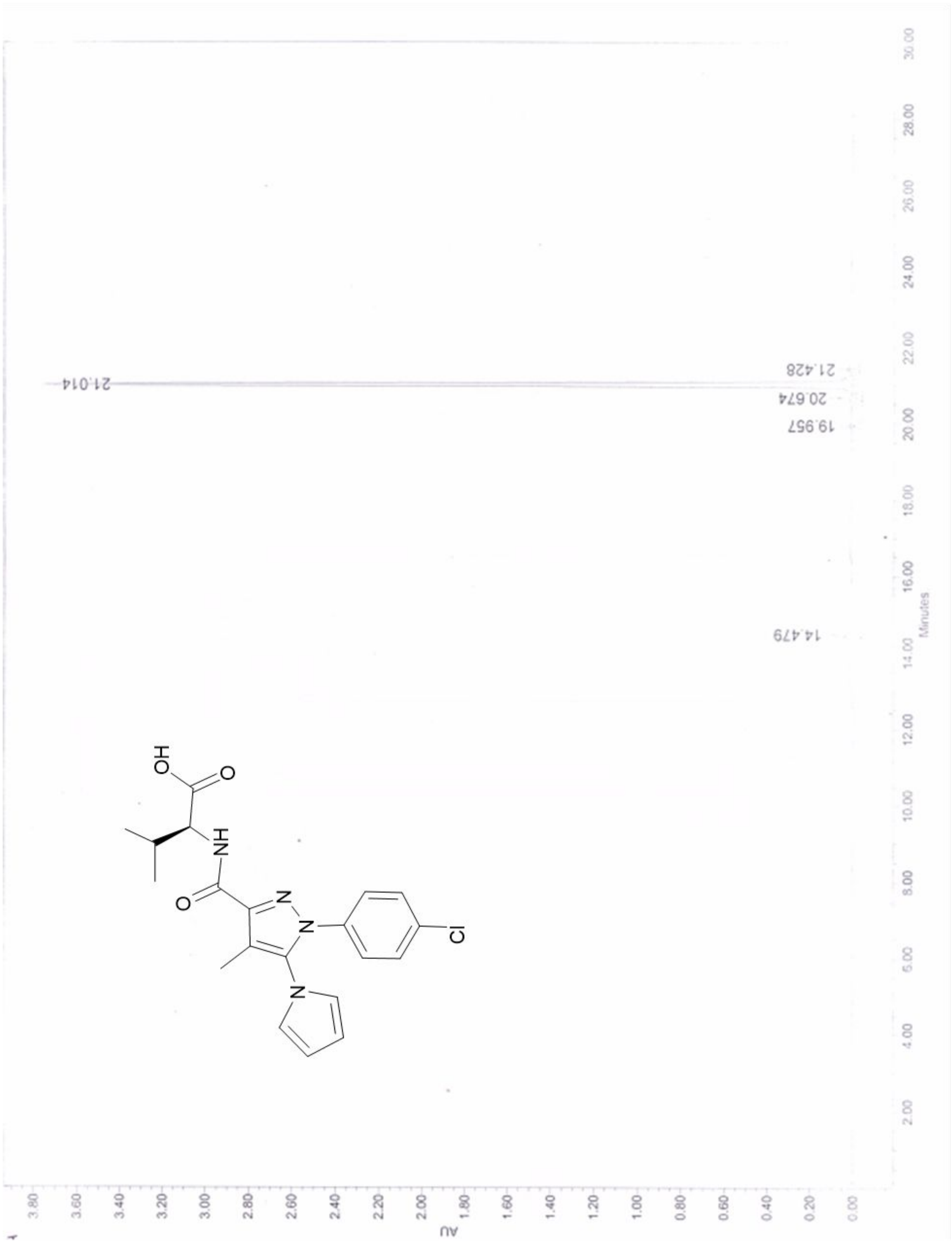

4

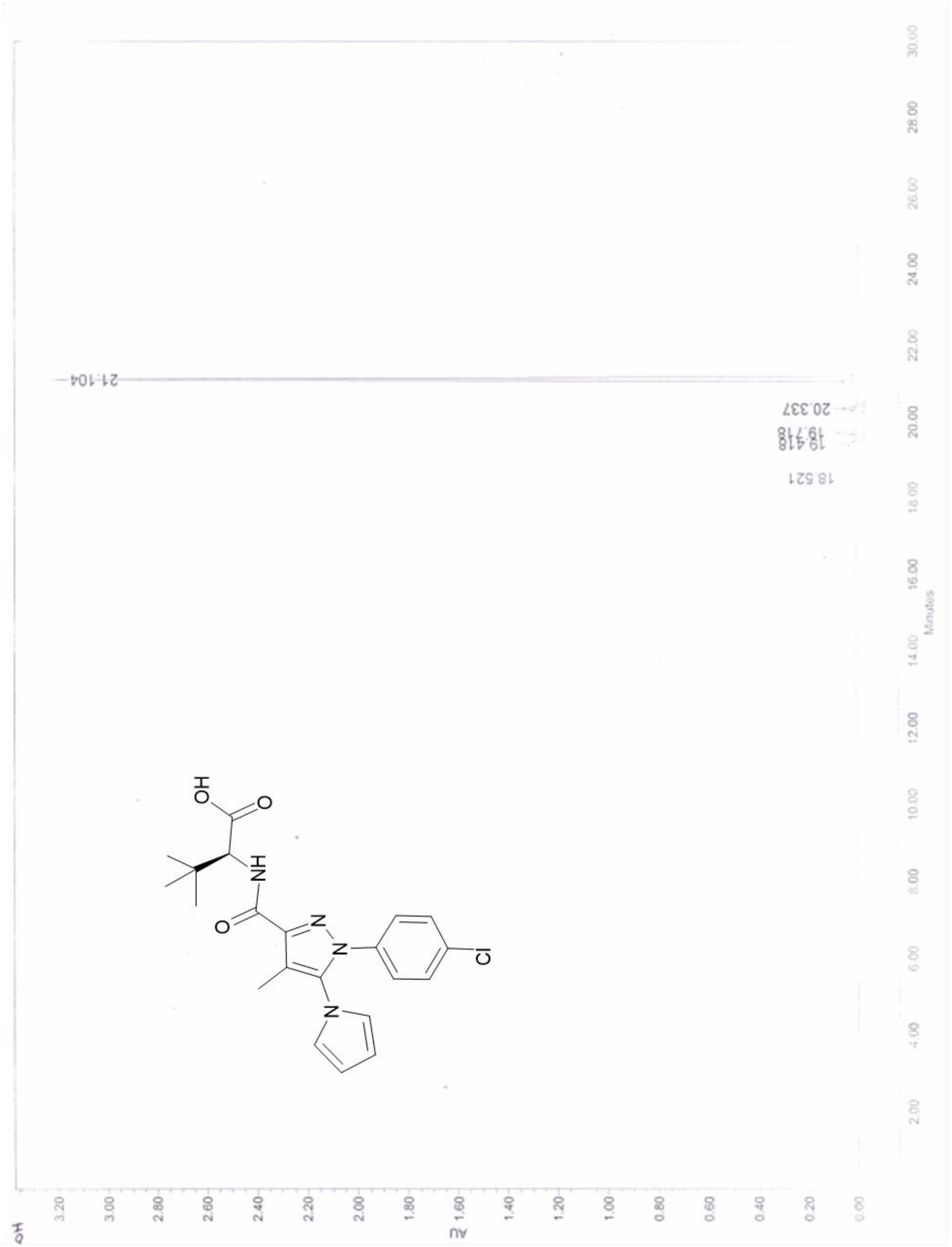

5

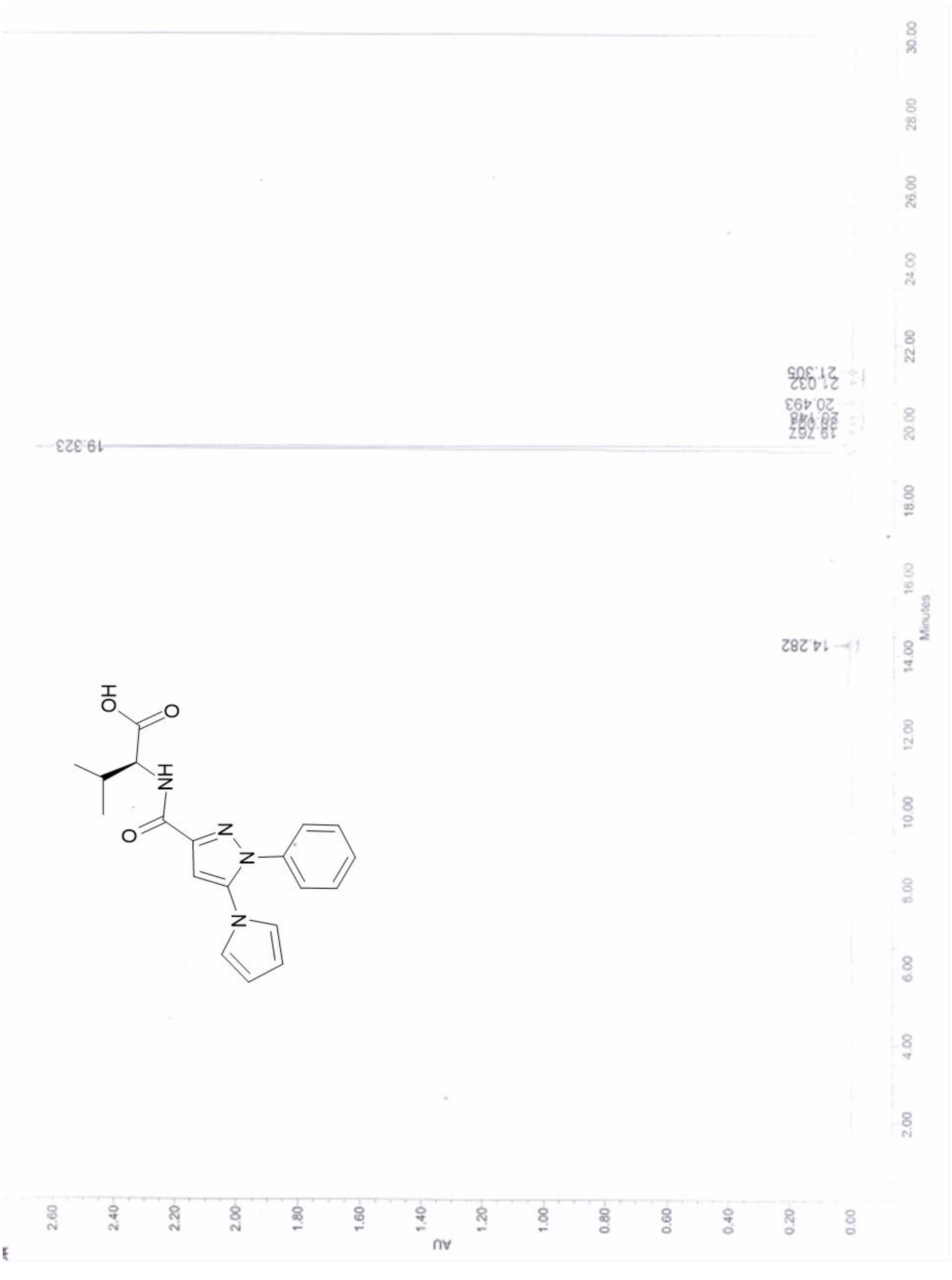

6

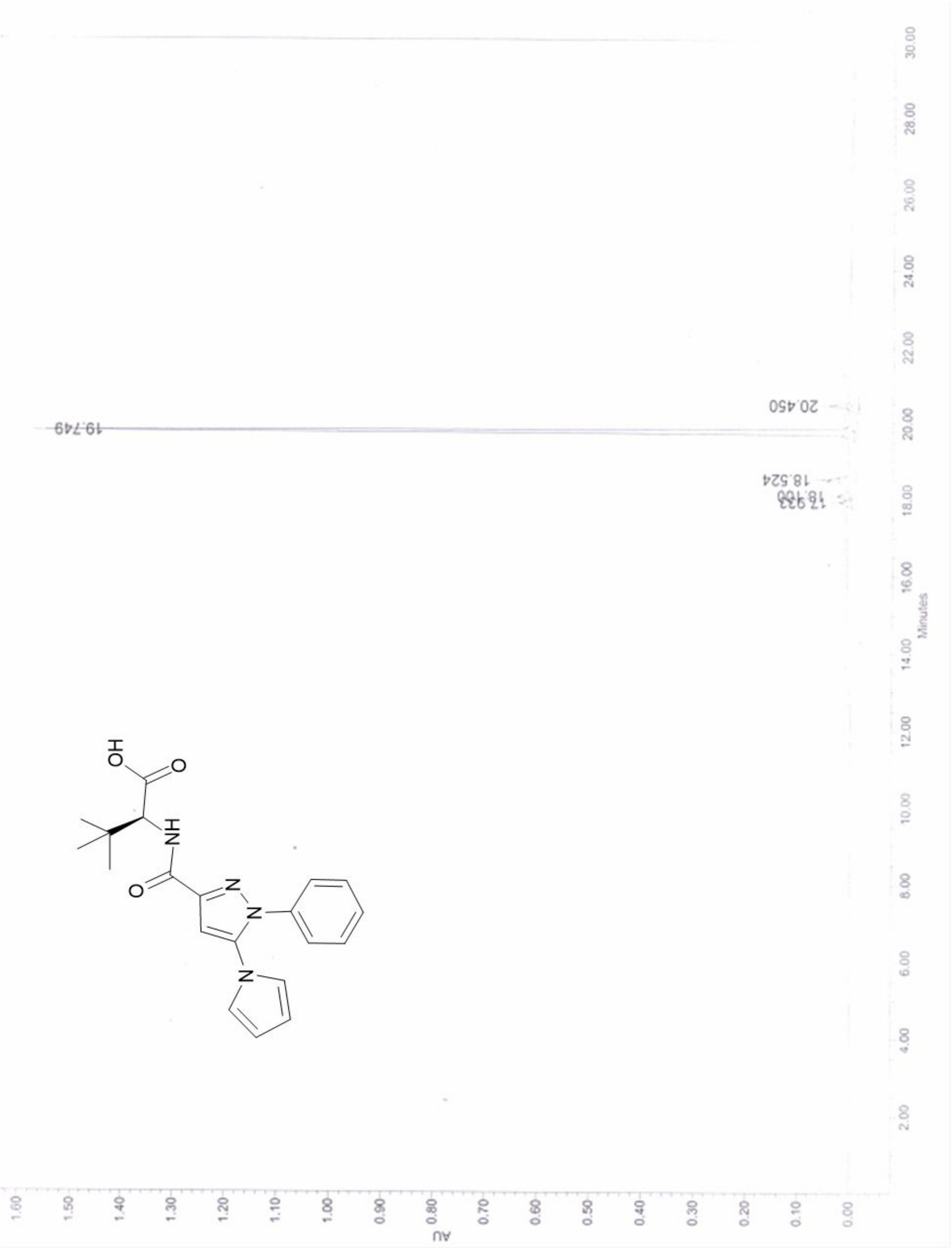

7

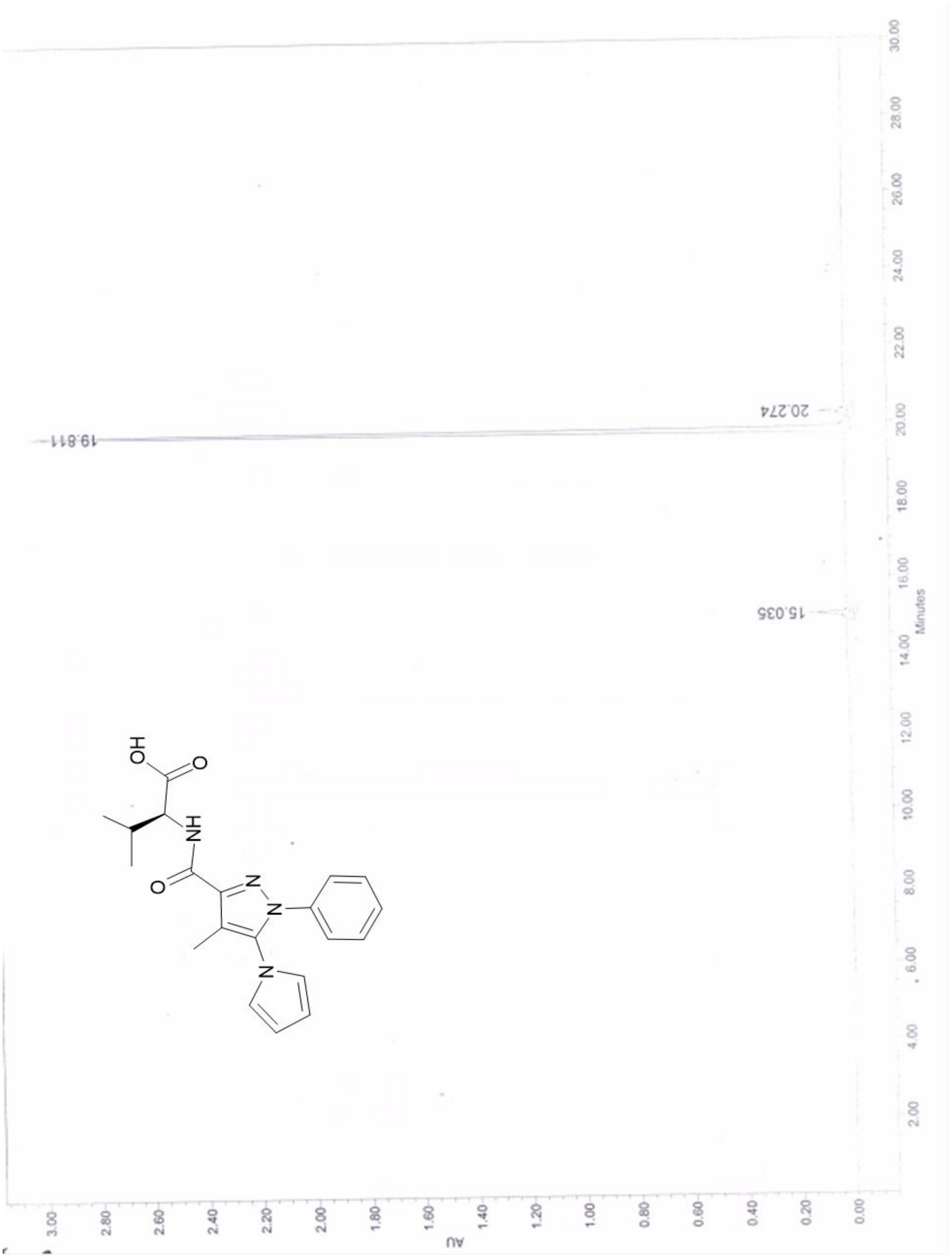

8

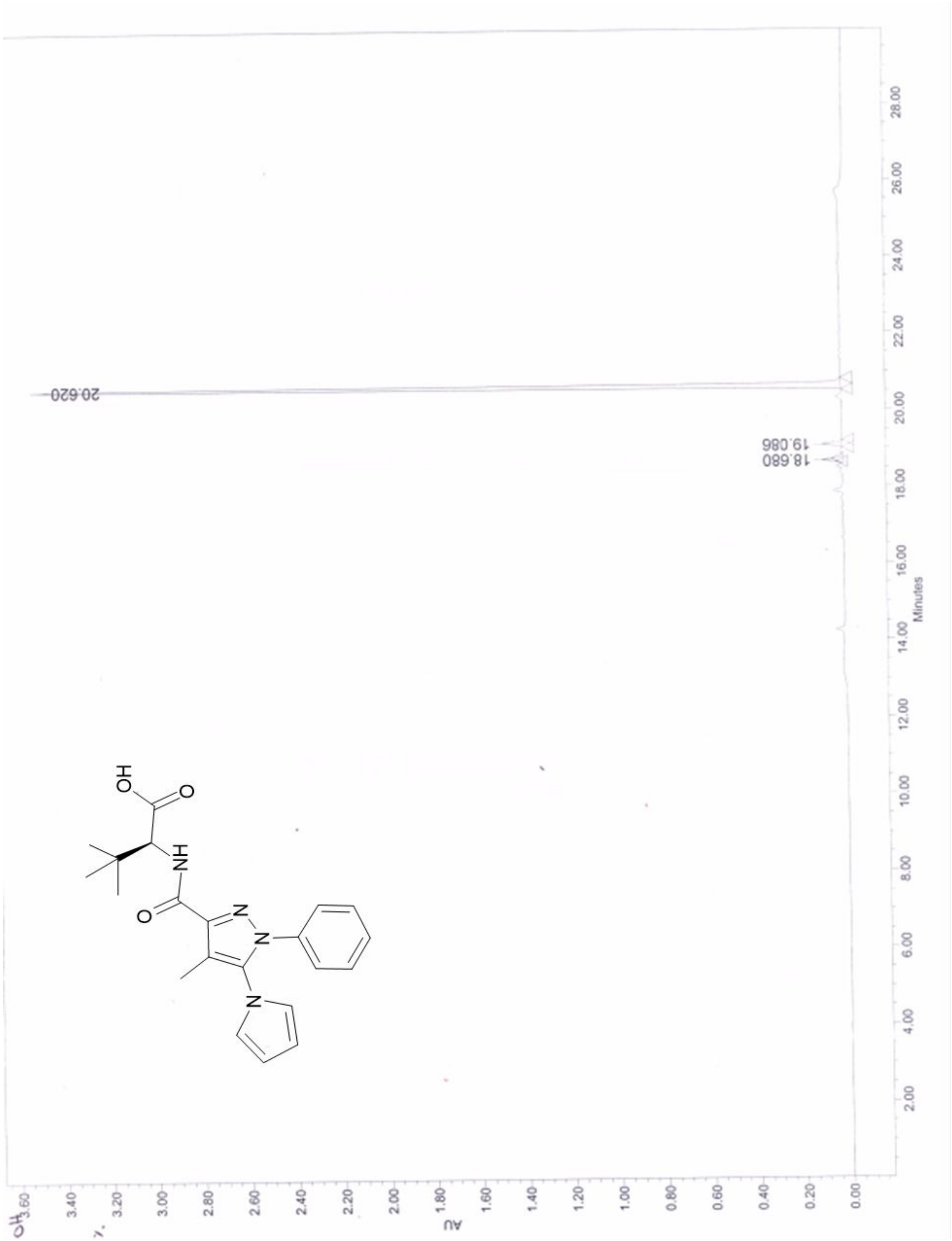

9

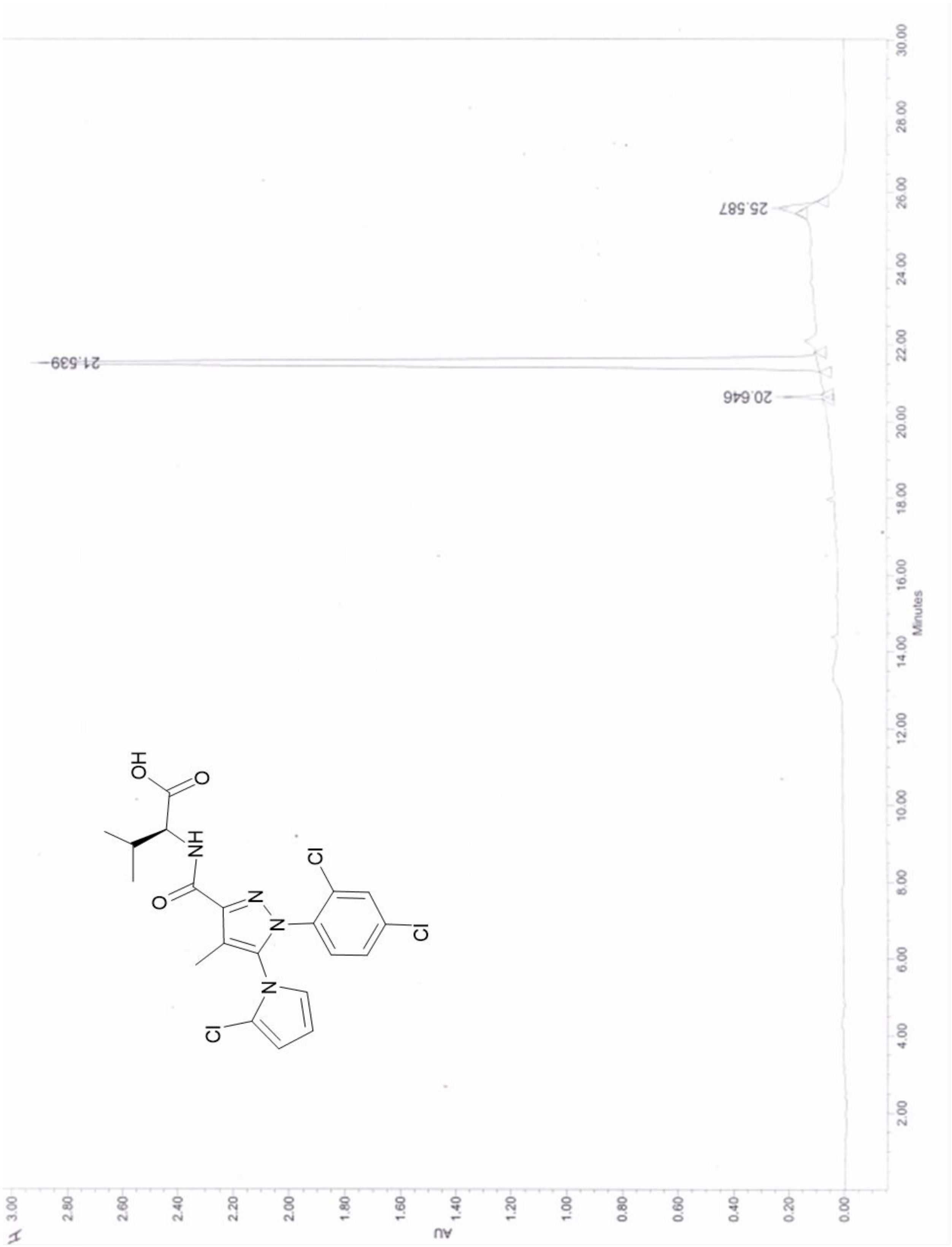

10

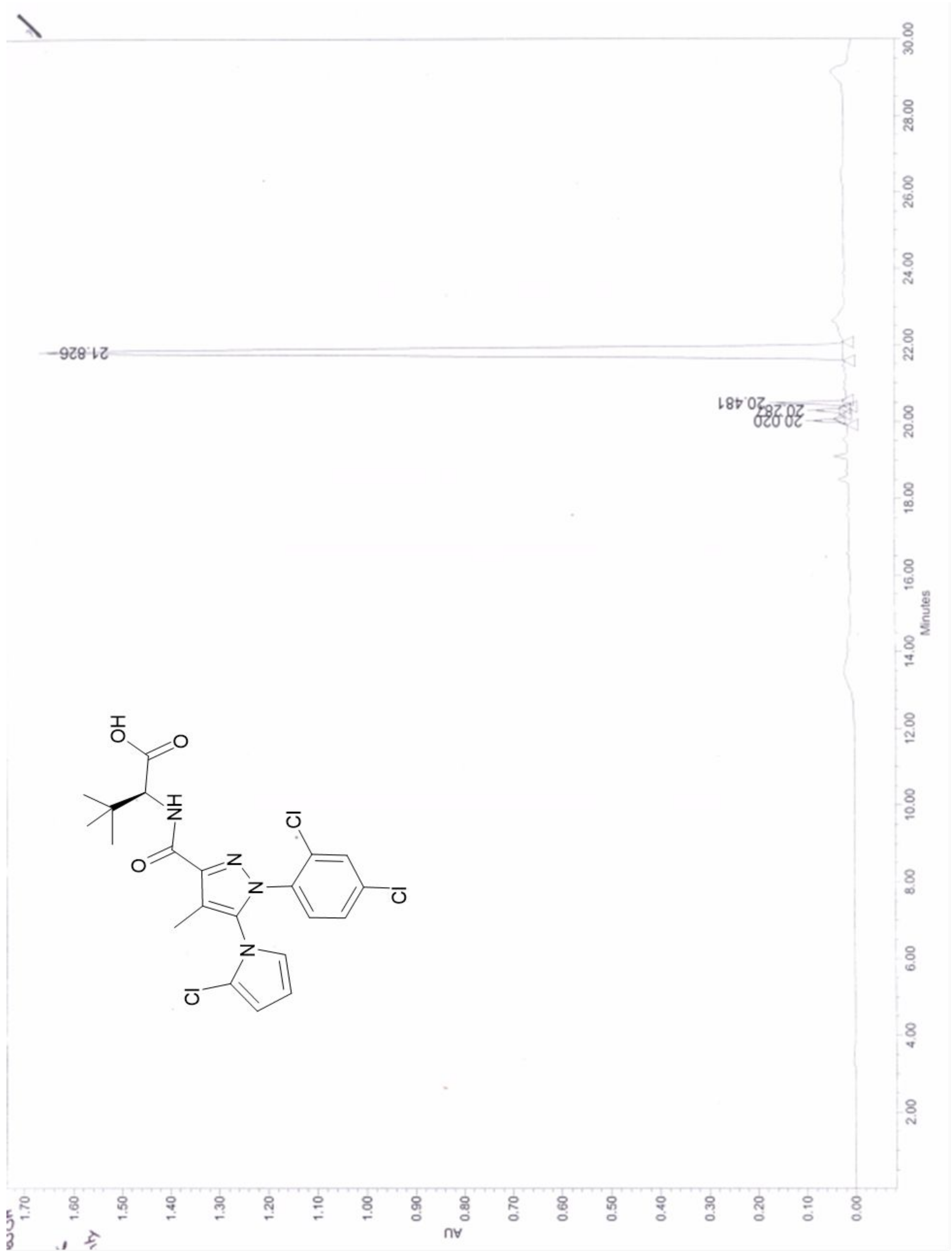

11

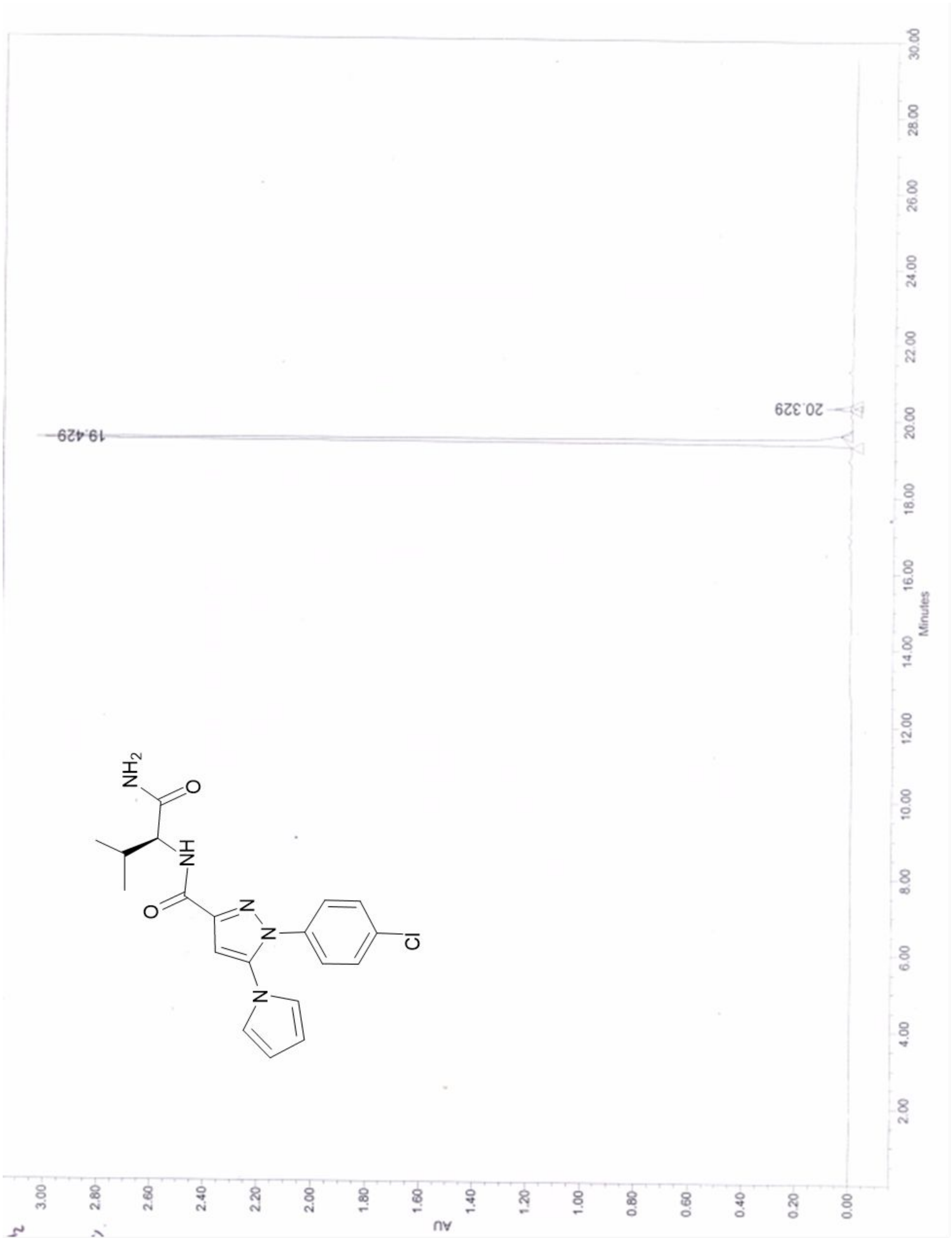

12

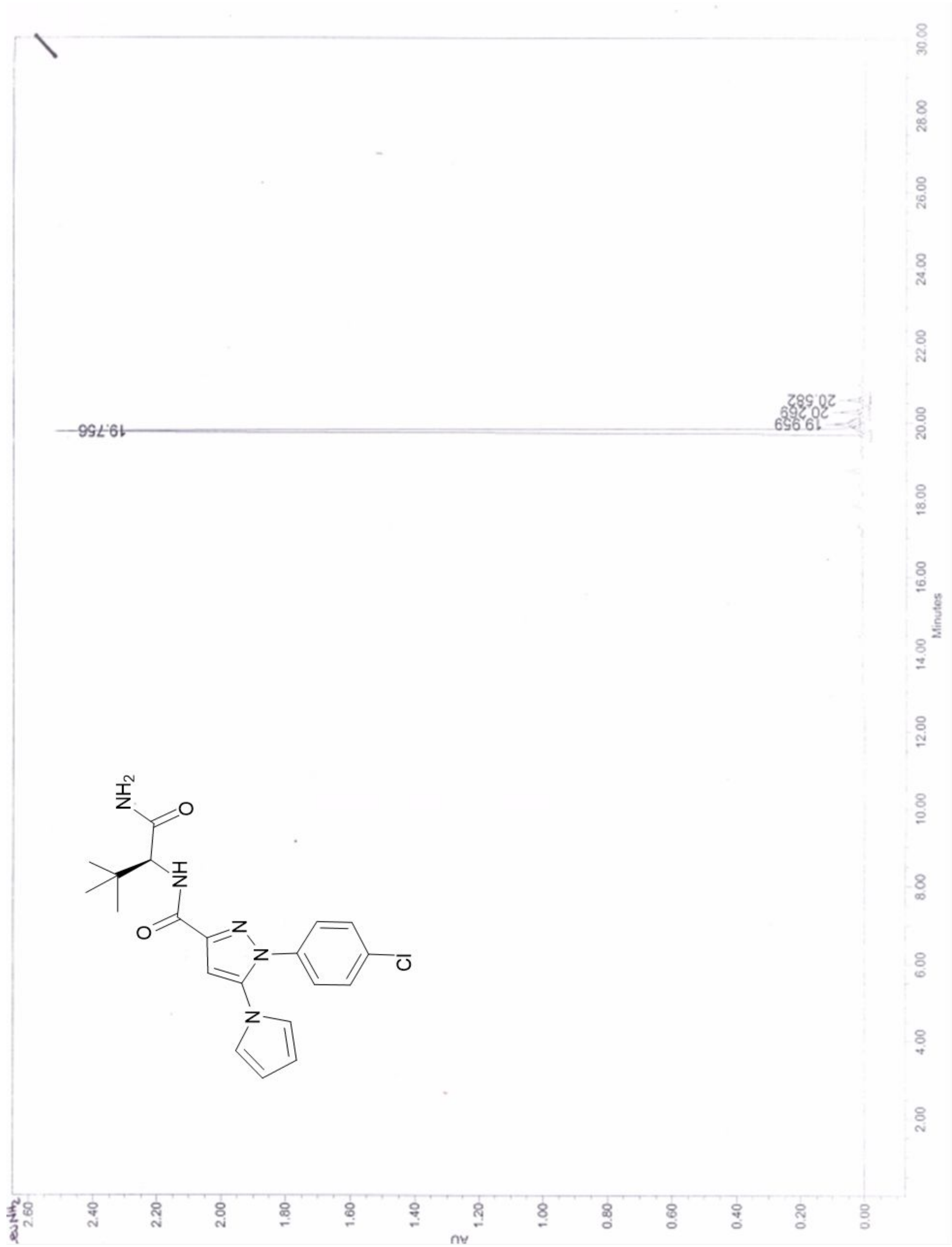

13

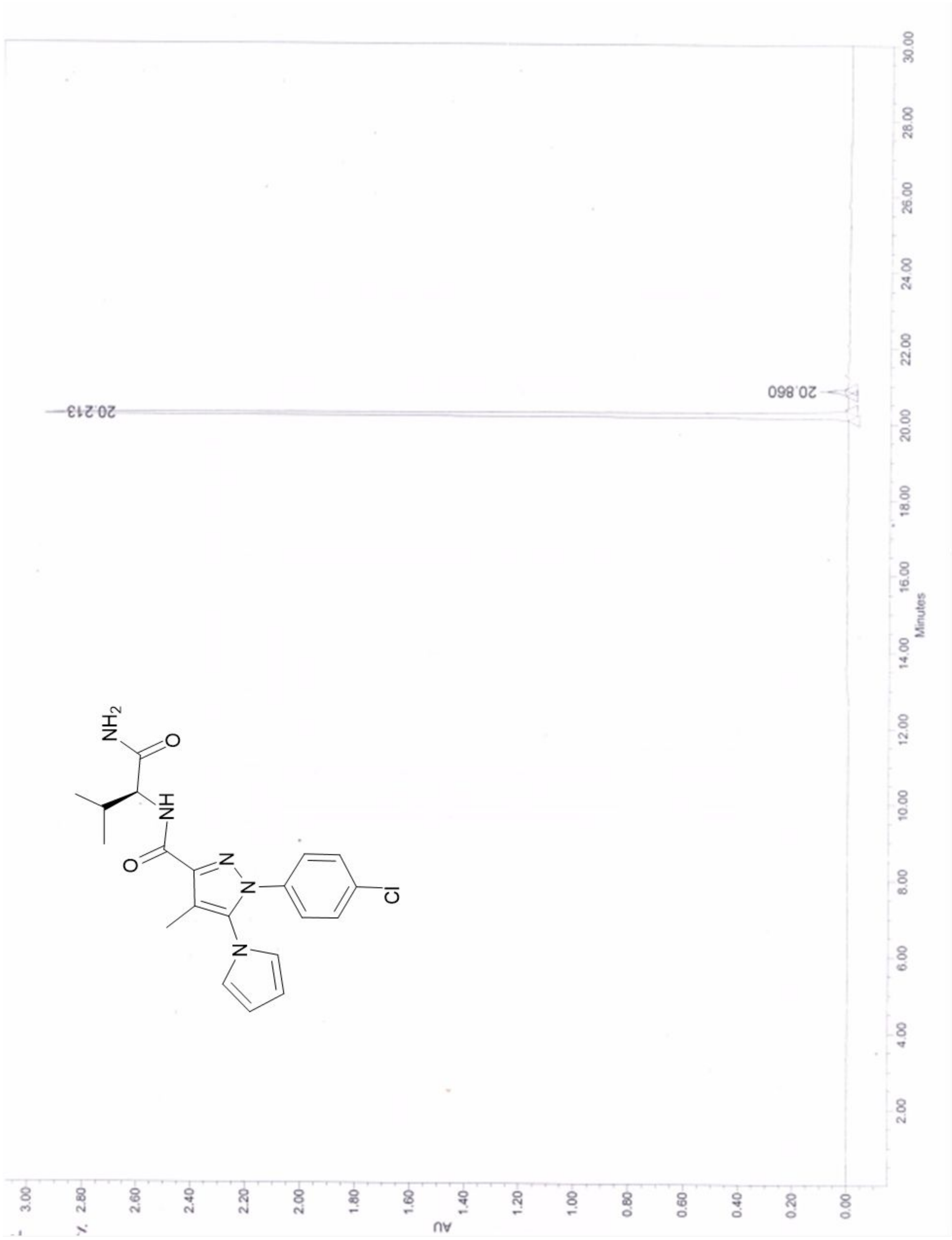

14

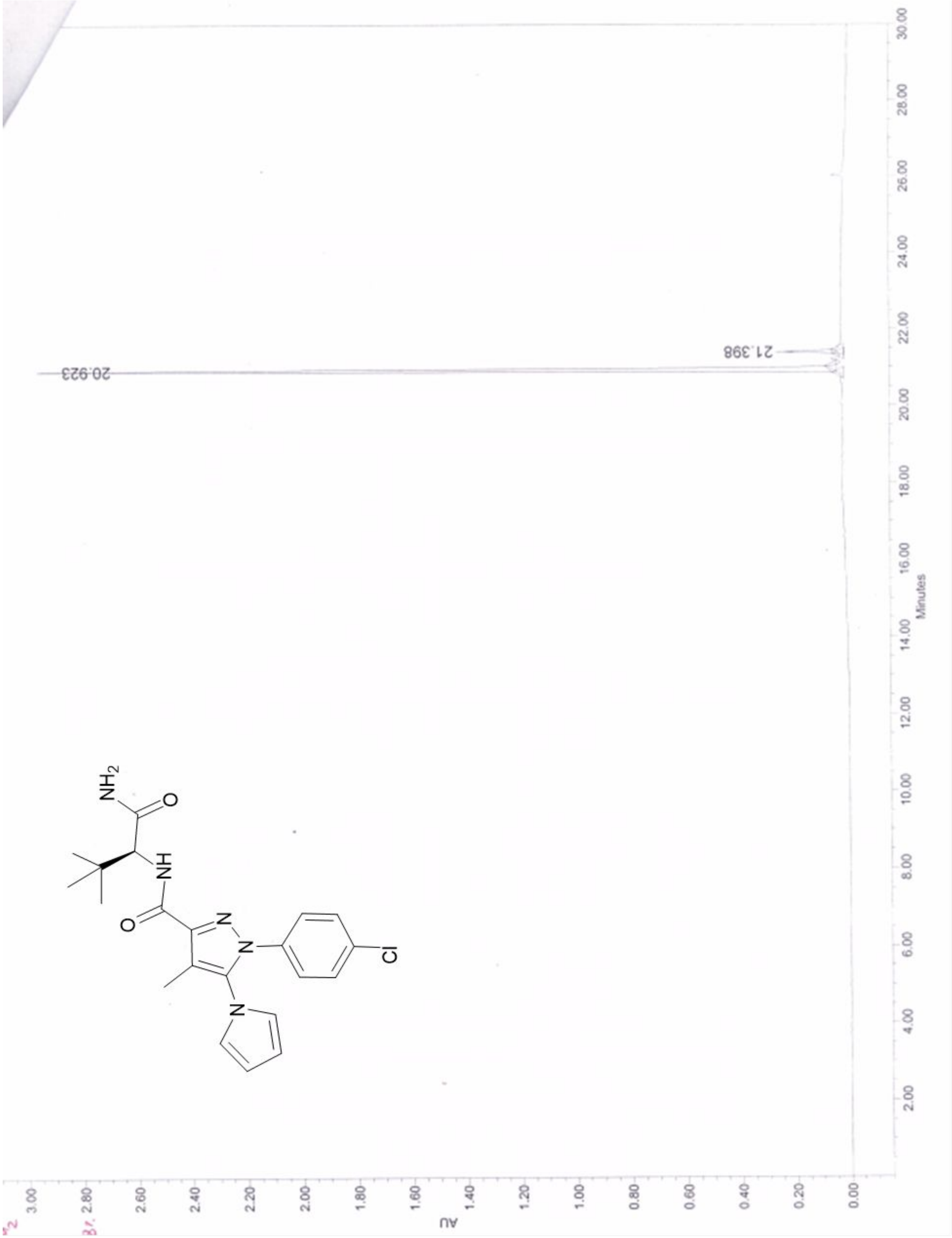

15

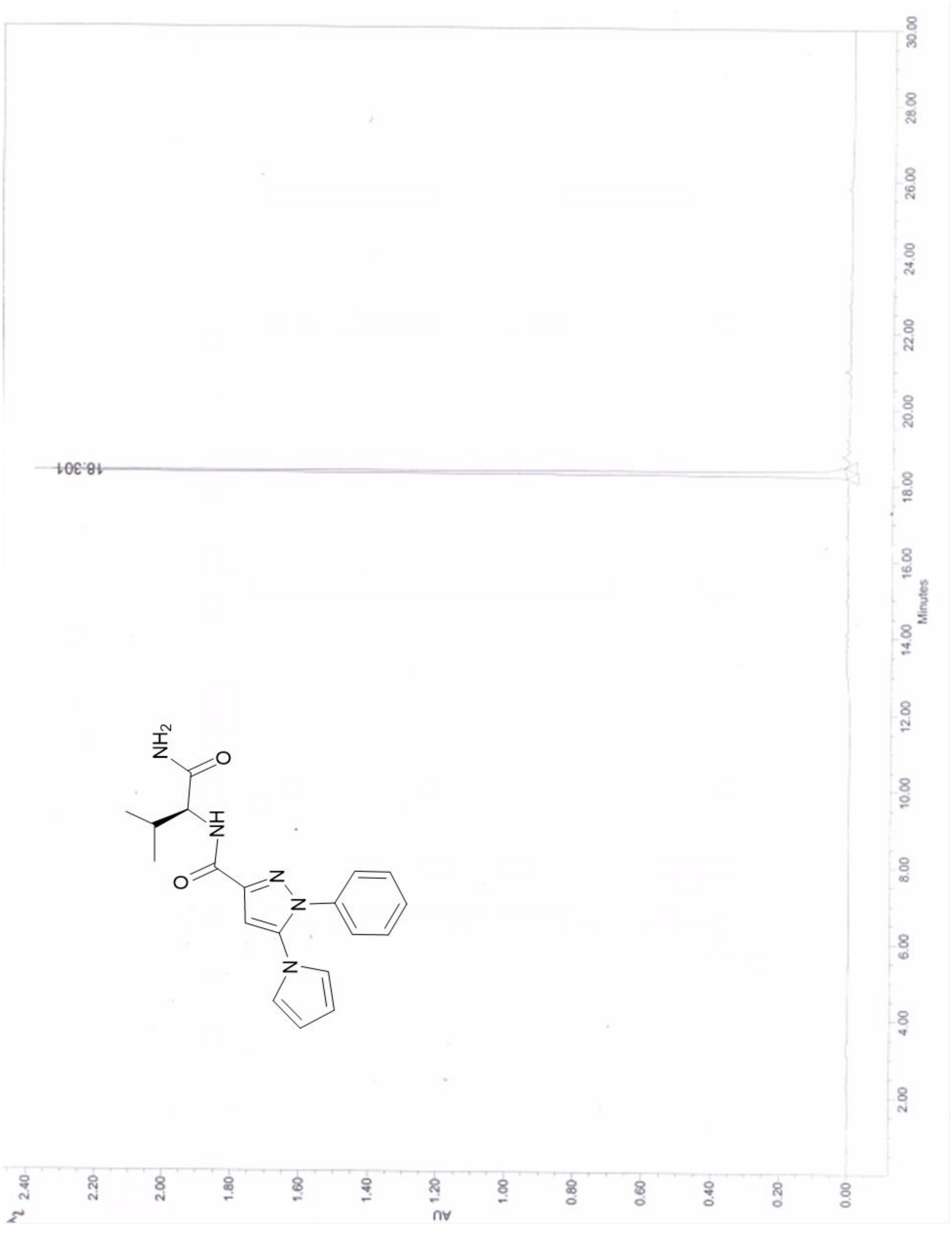

16

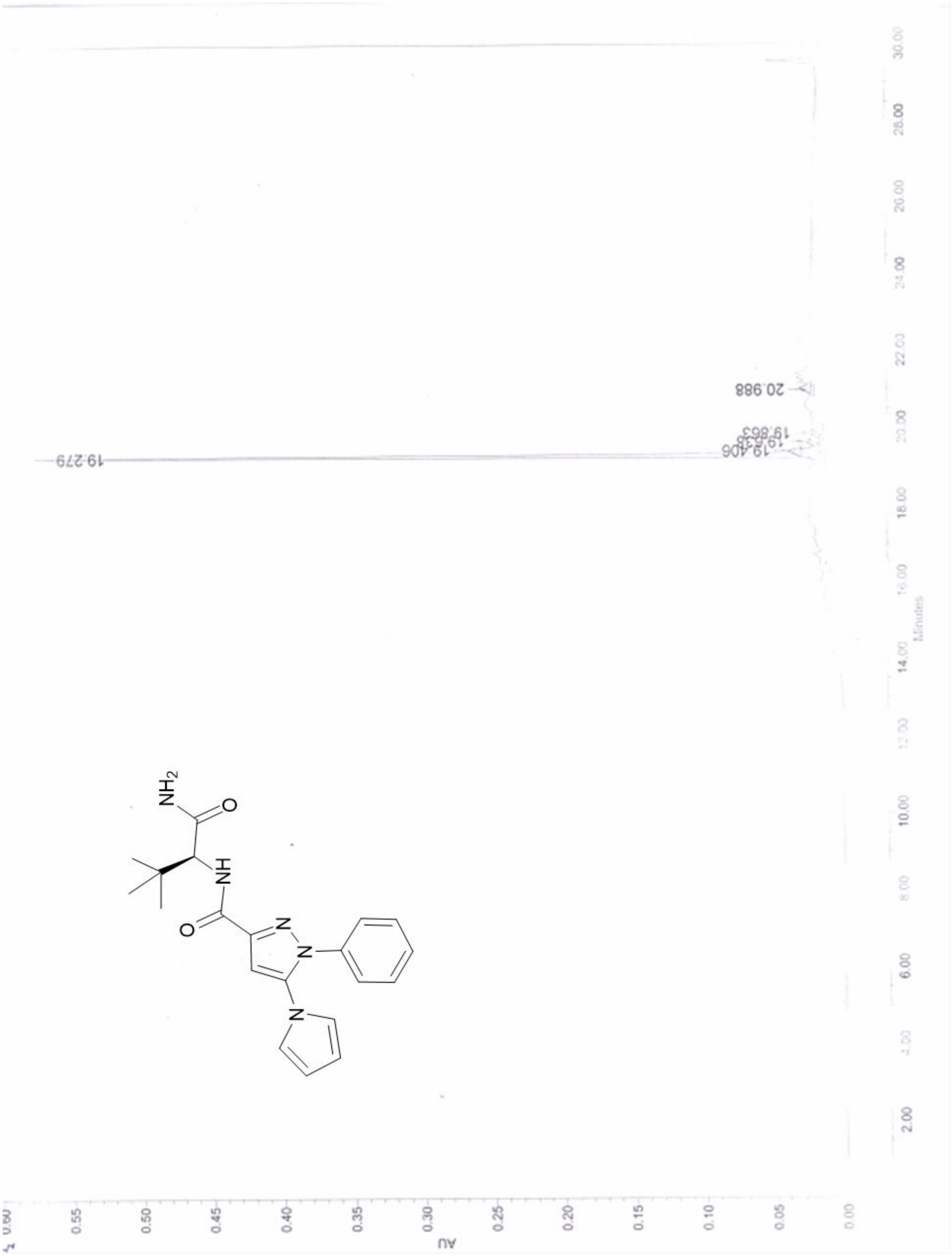

17

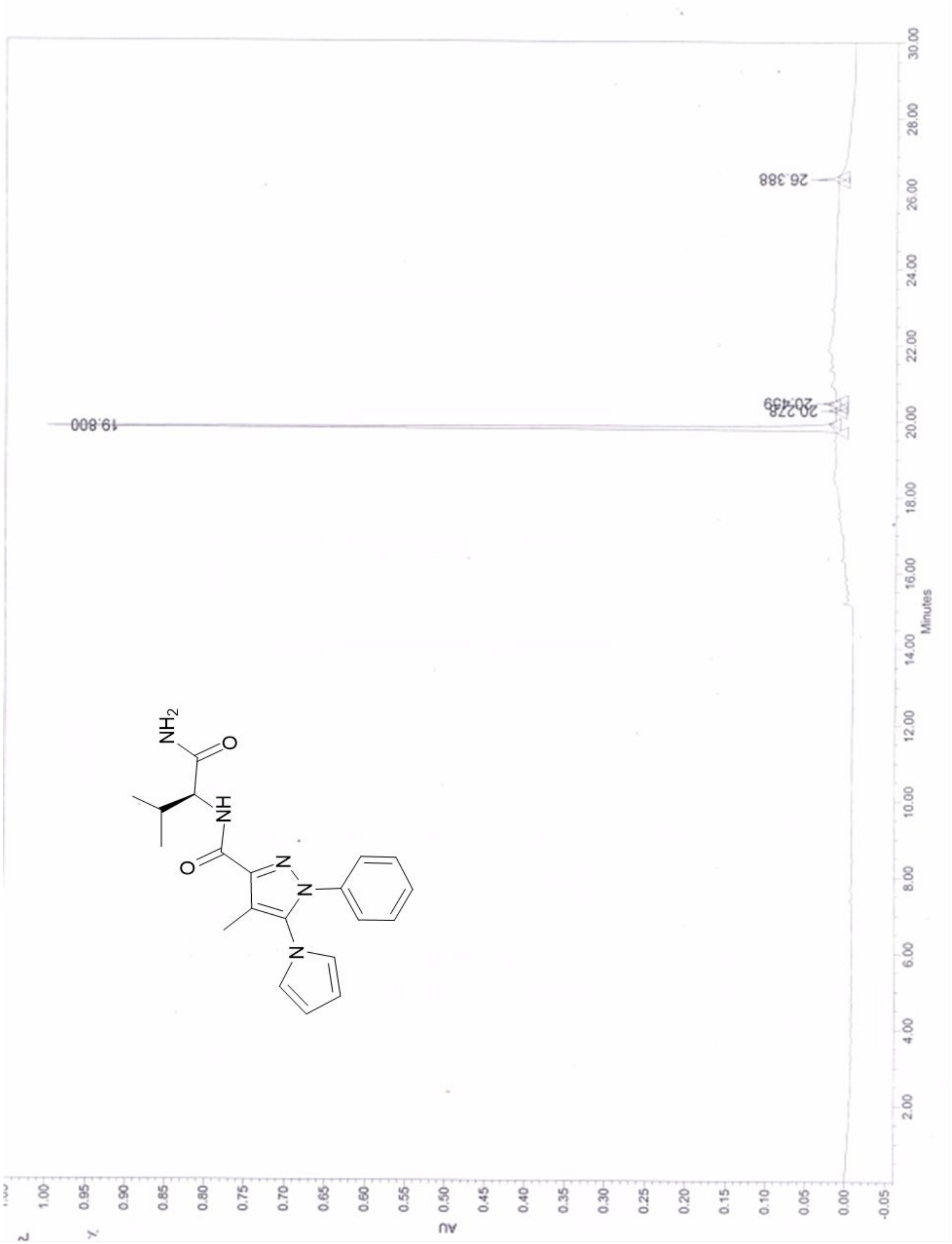

18

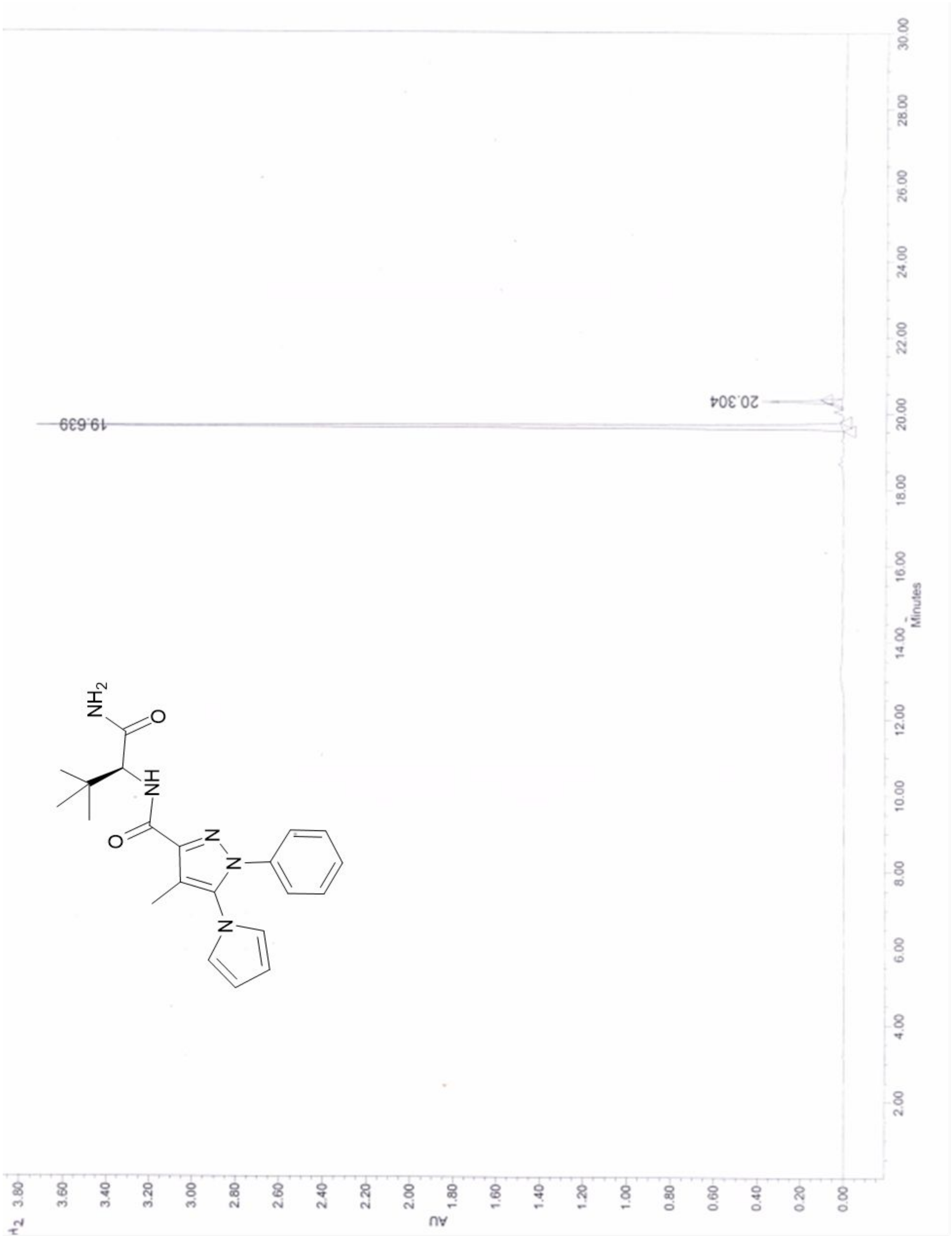

19

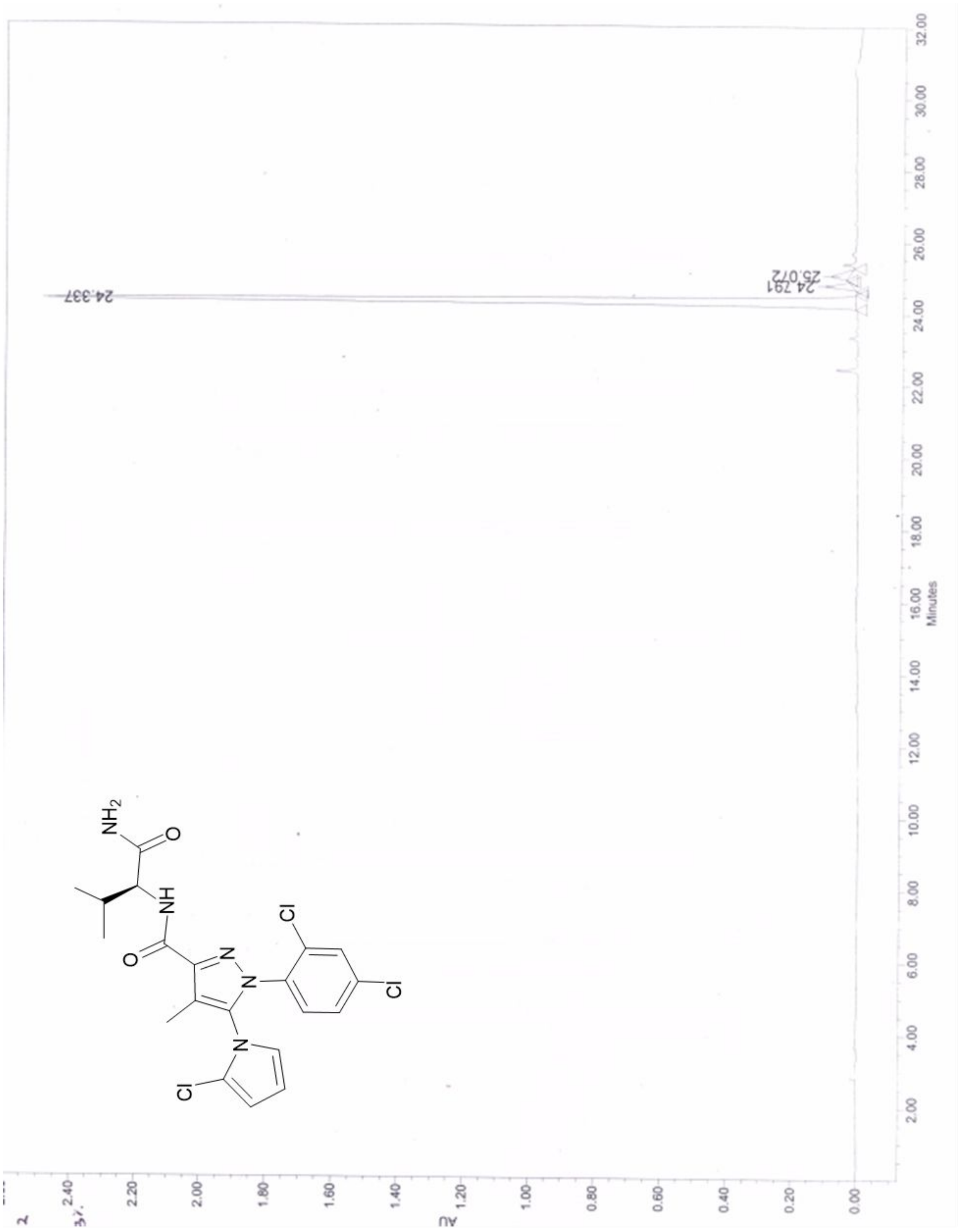

20

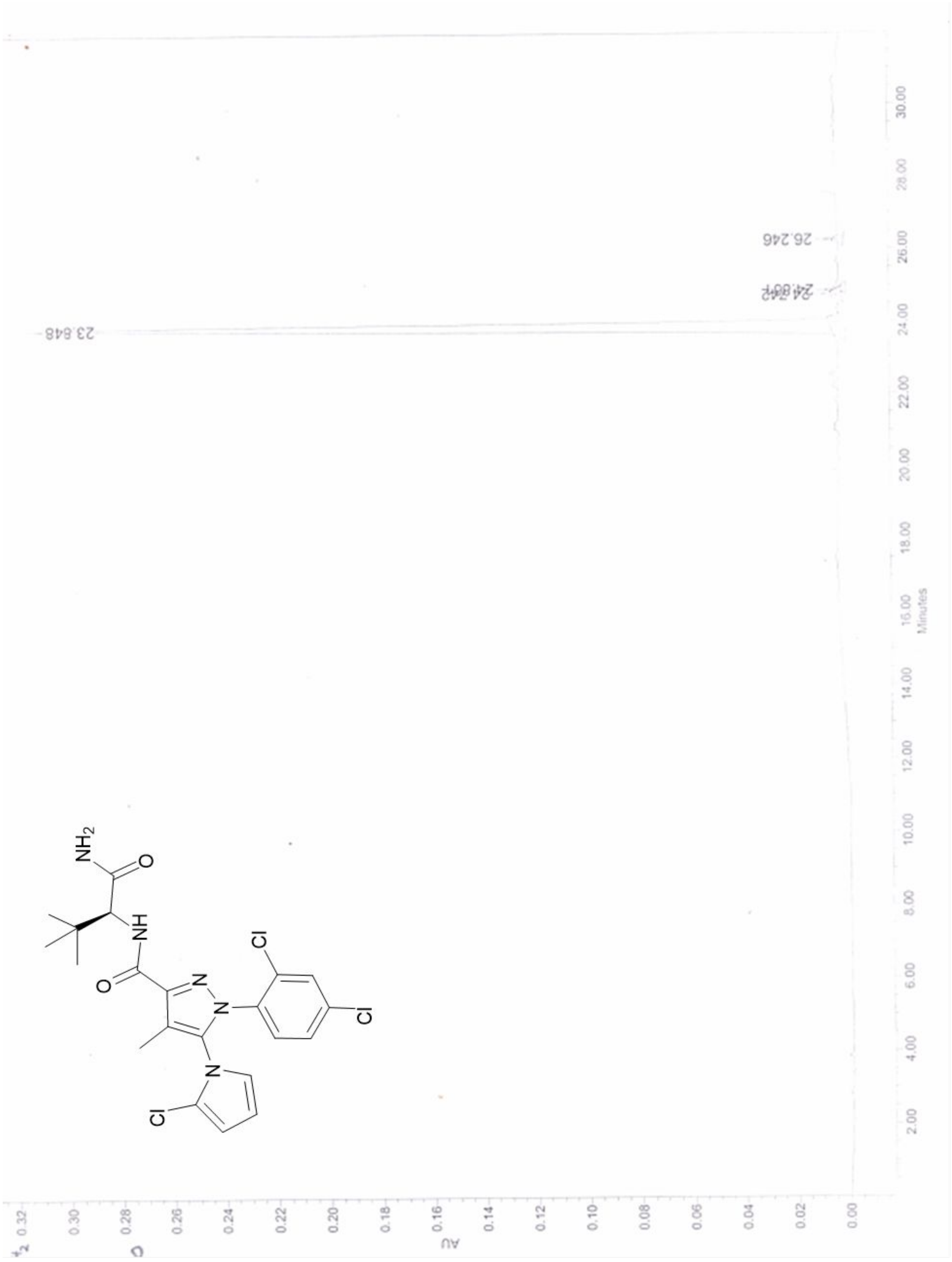

21

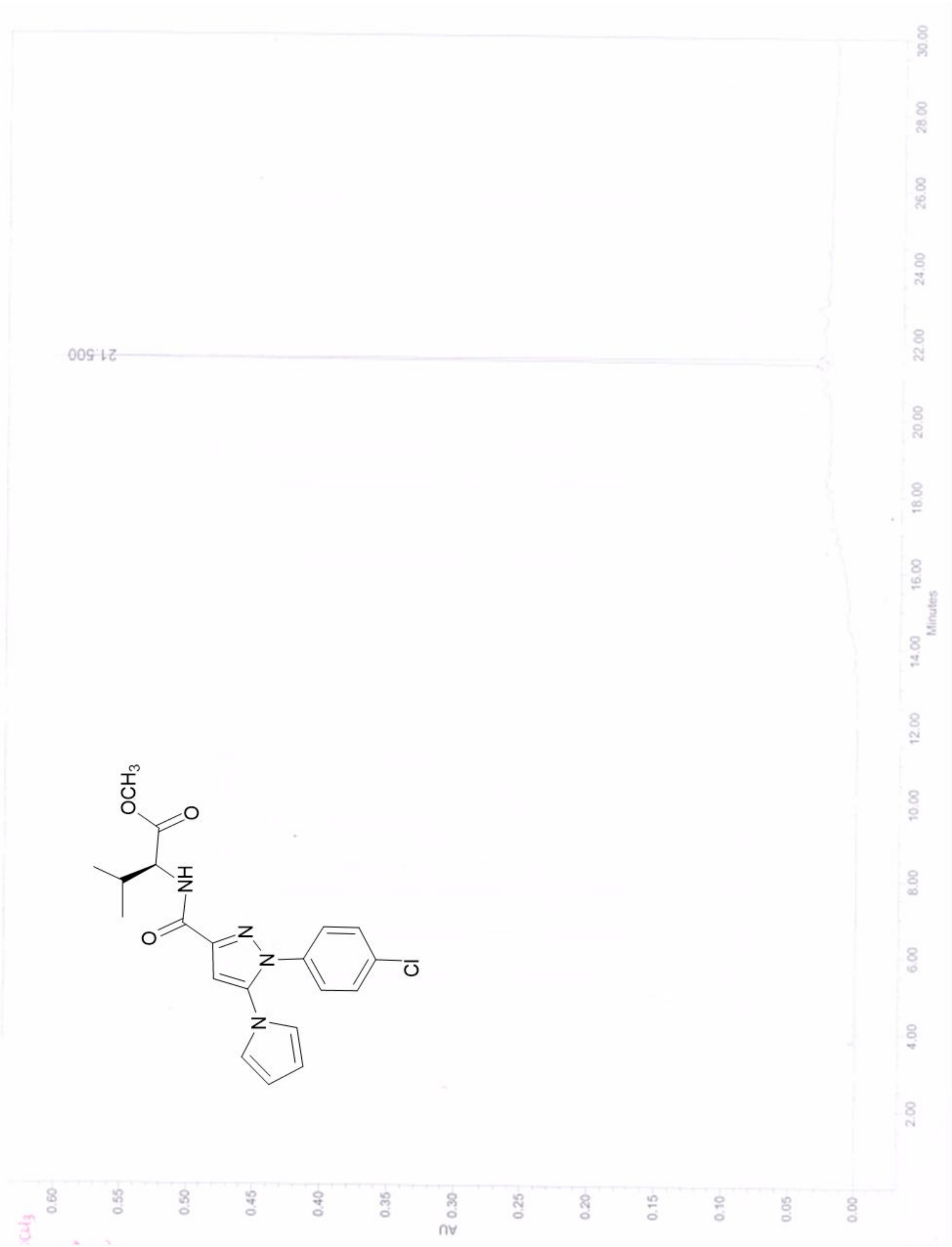

22

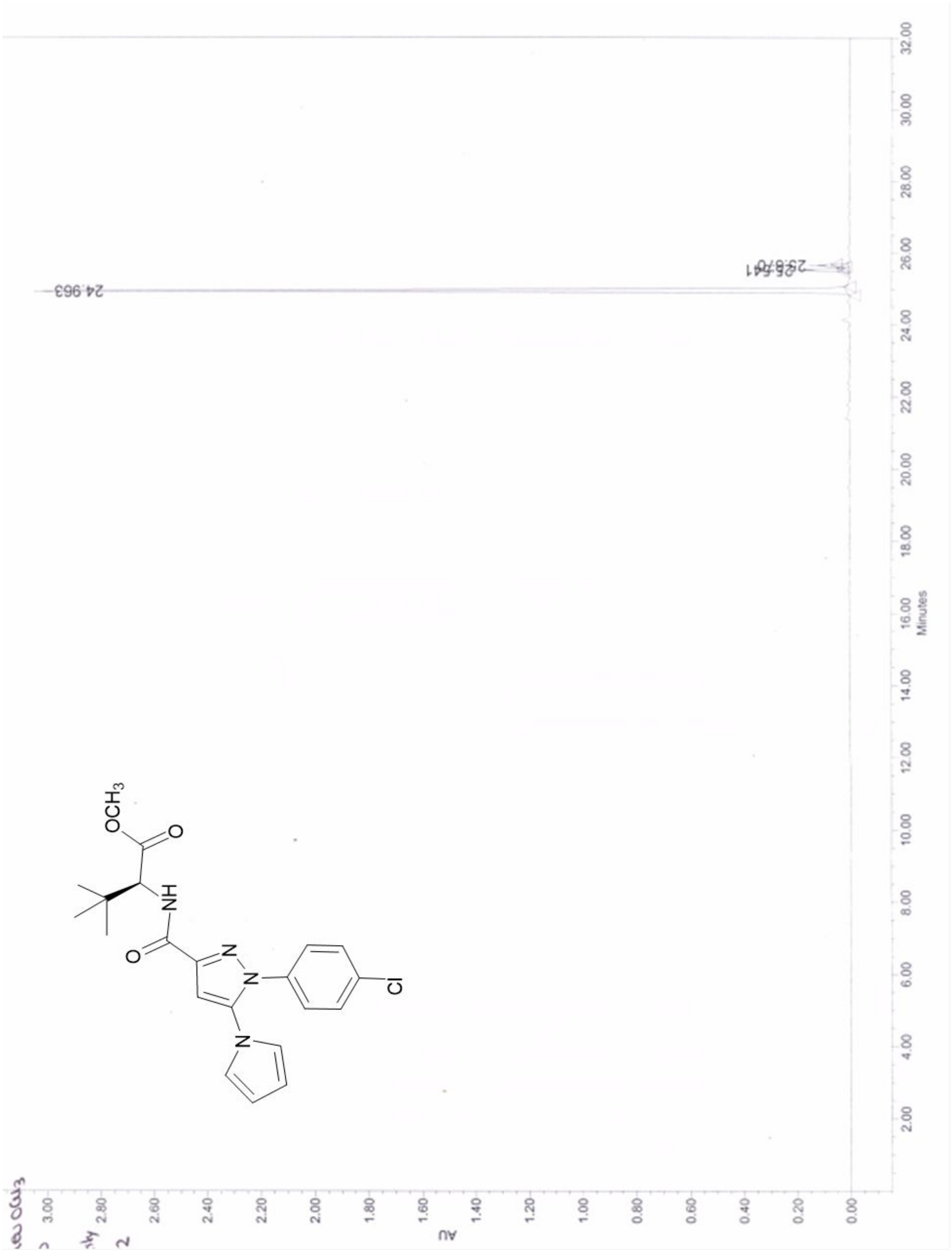

23

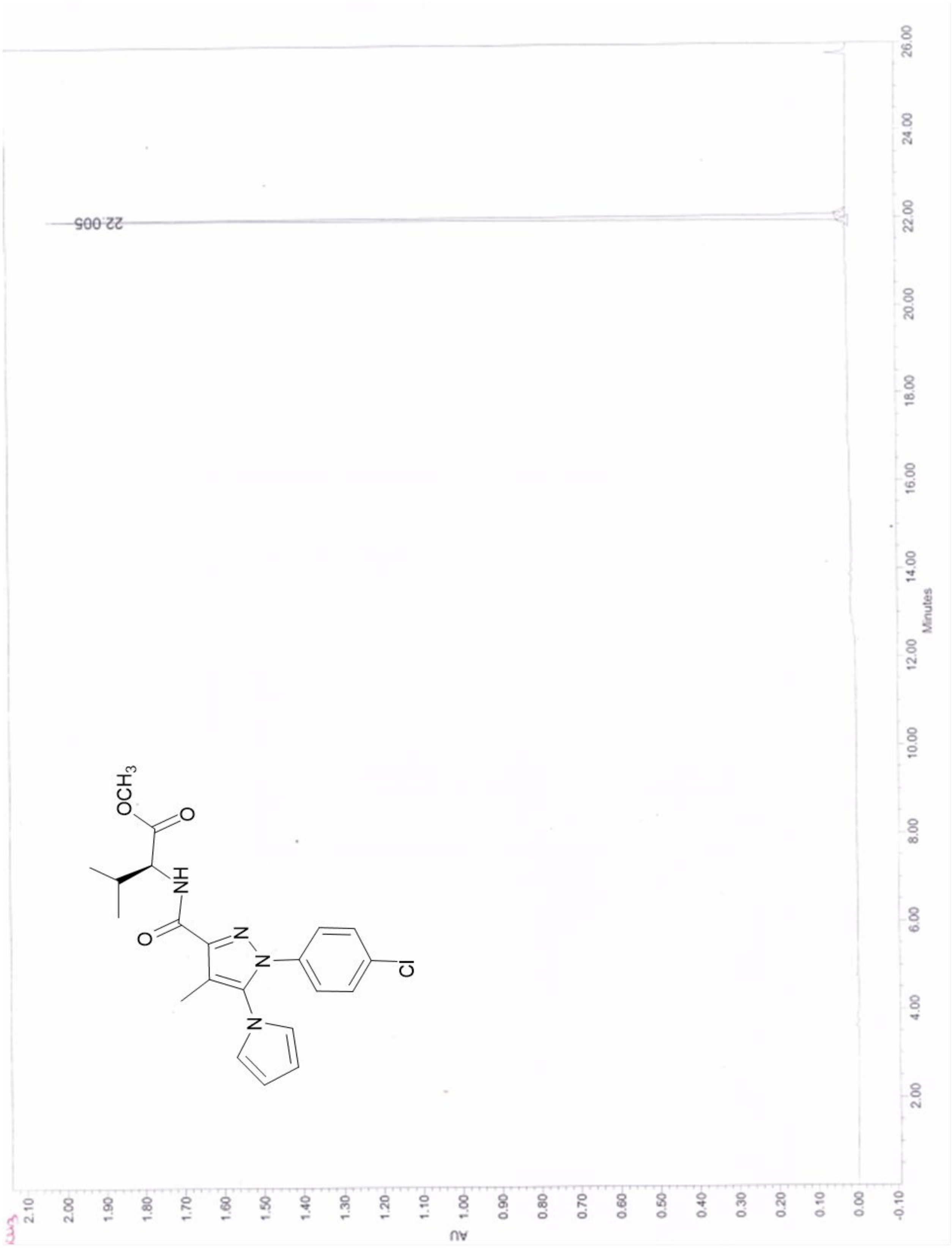

24

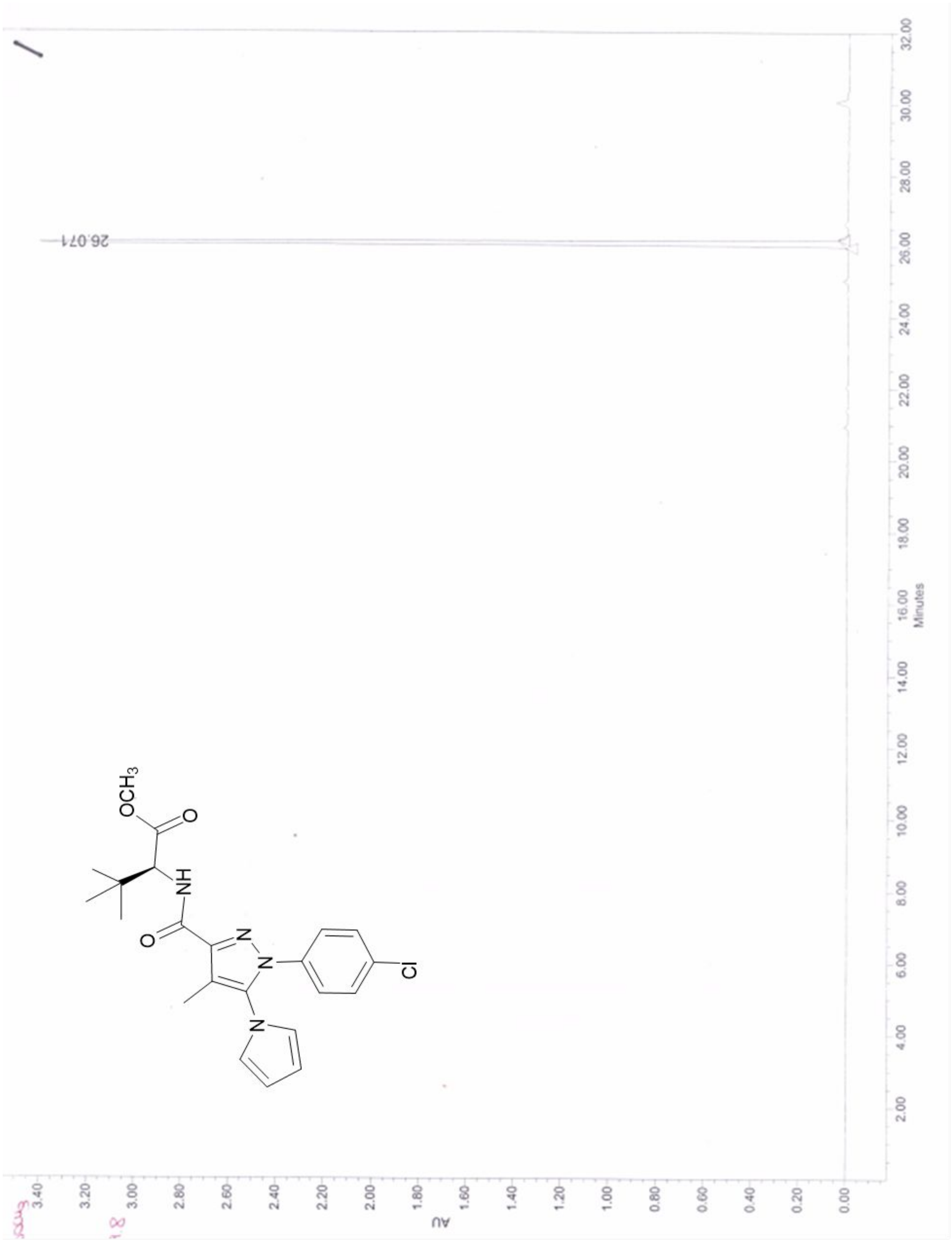

25

26

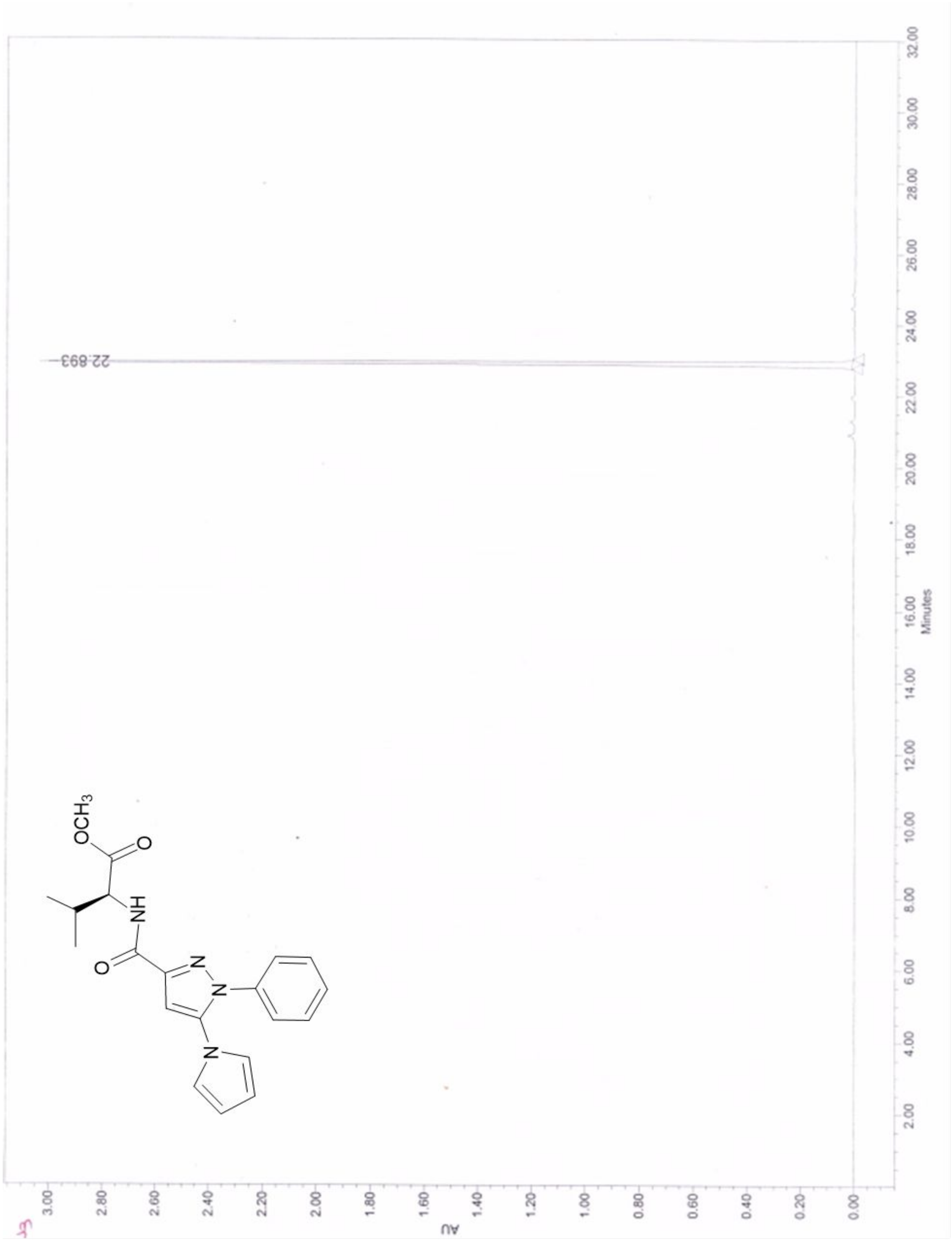

26

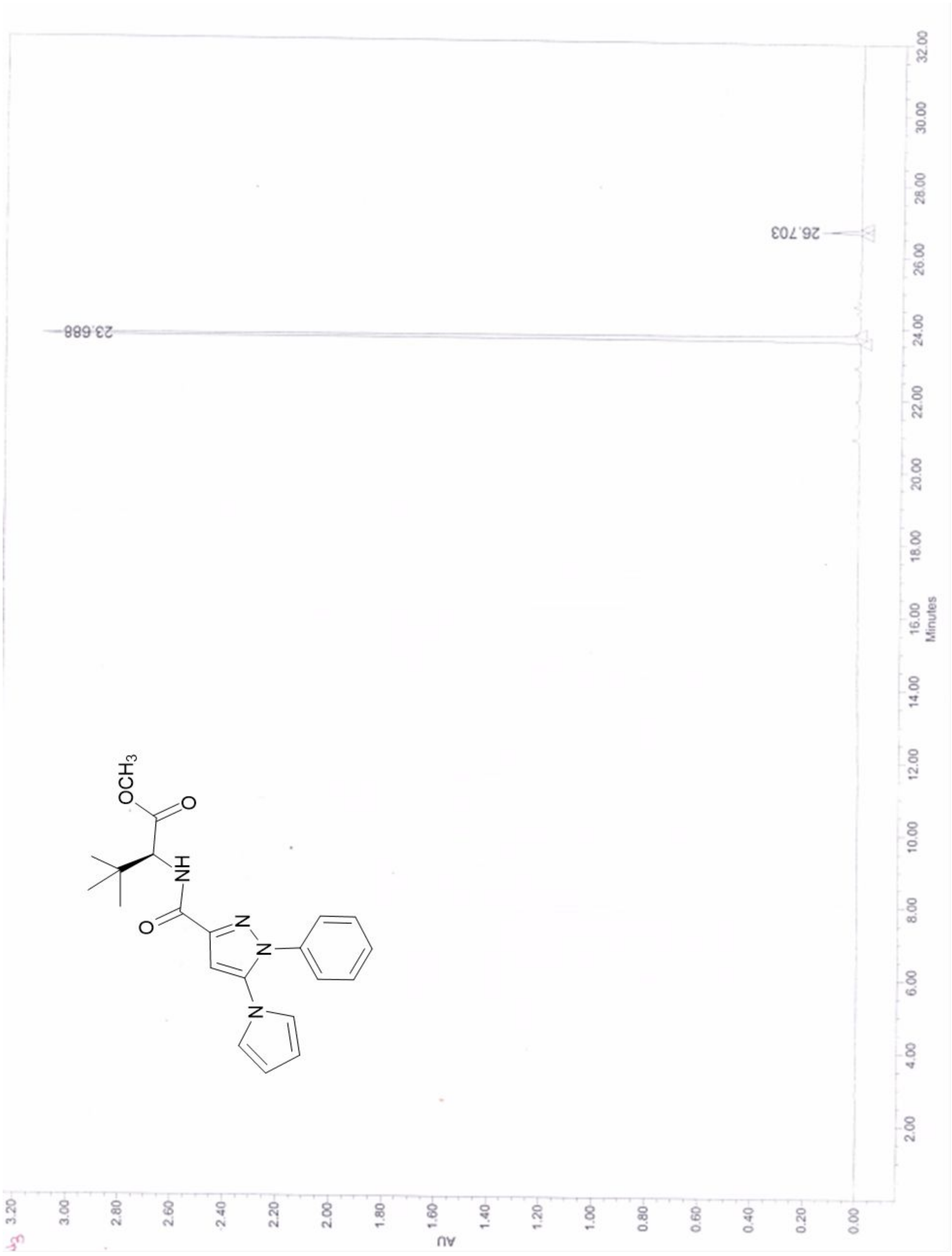

27

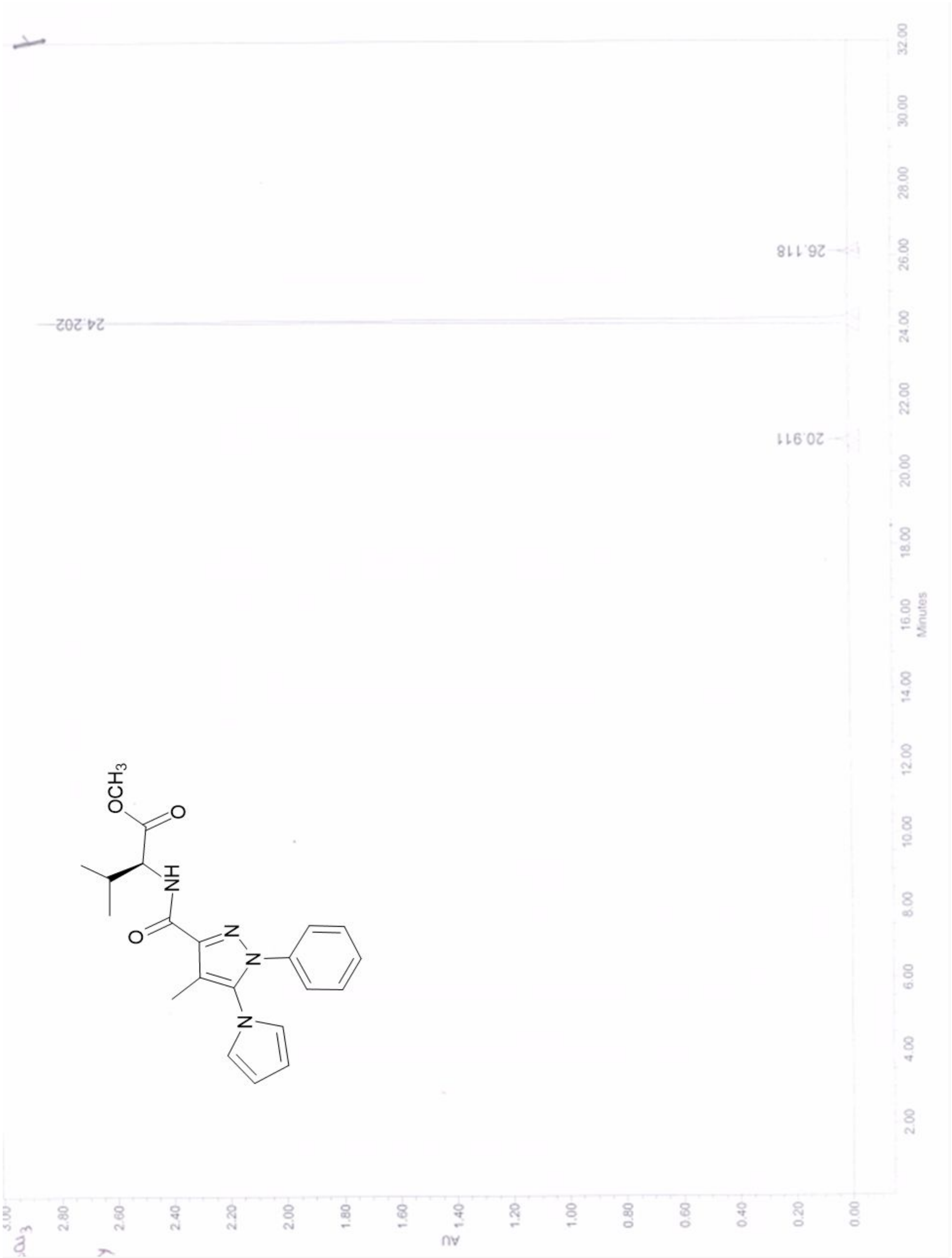

28

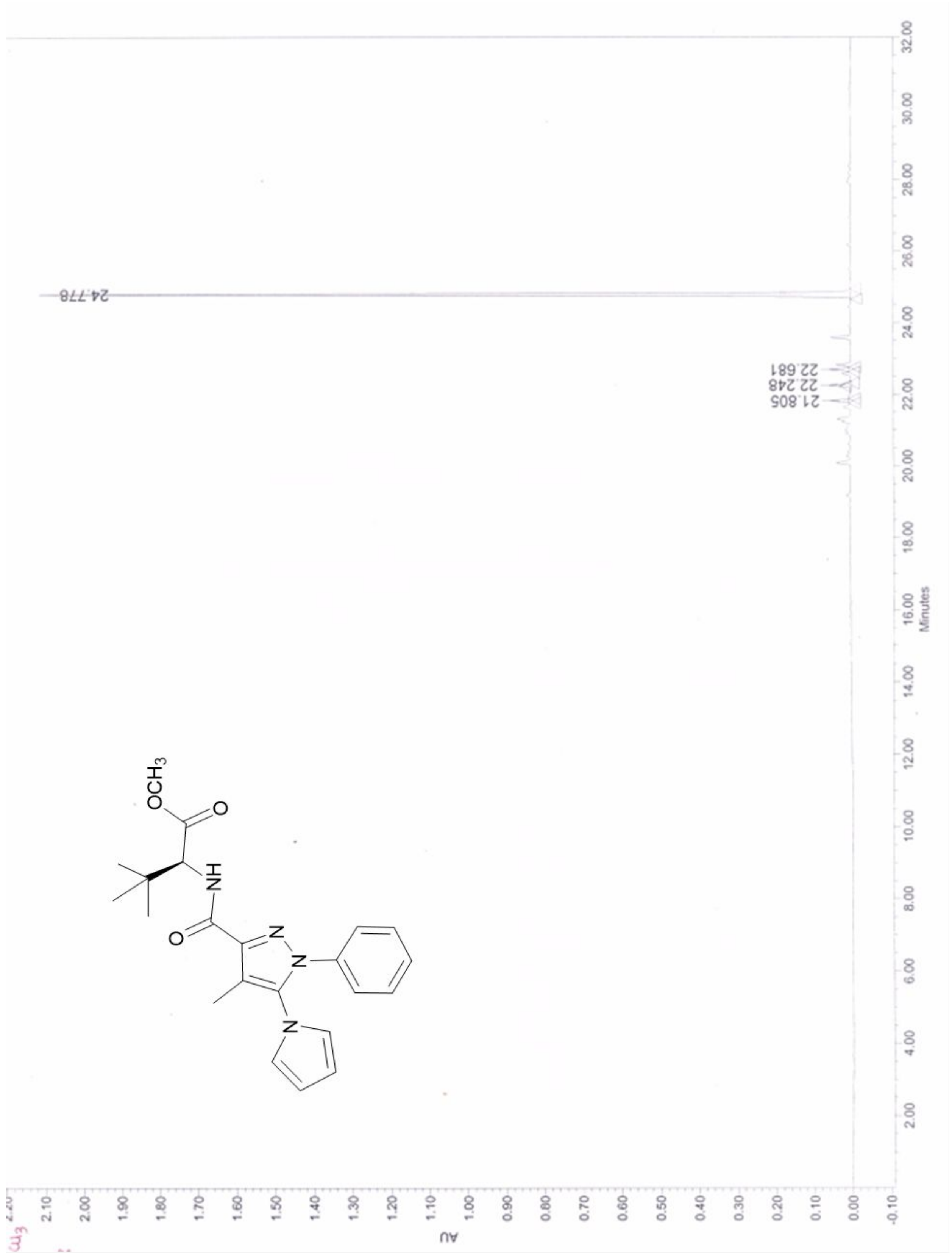

29

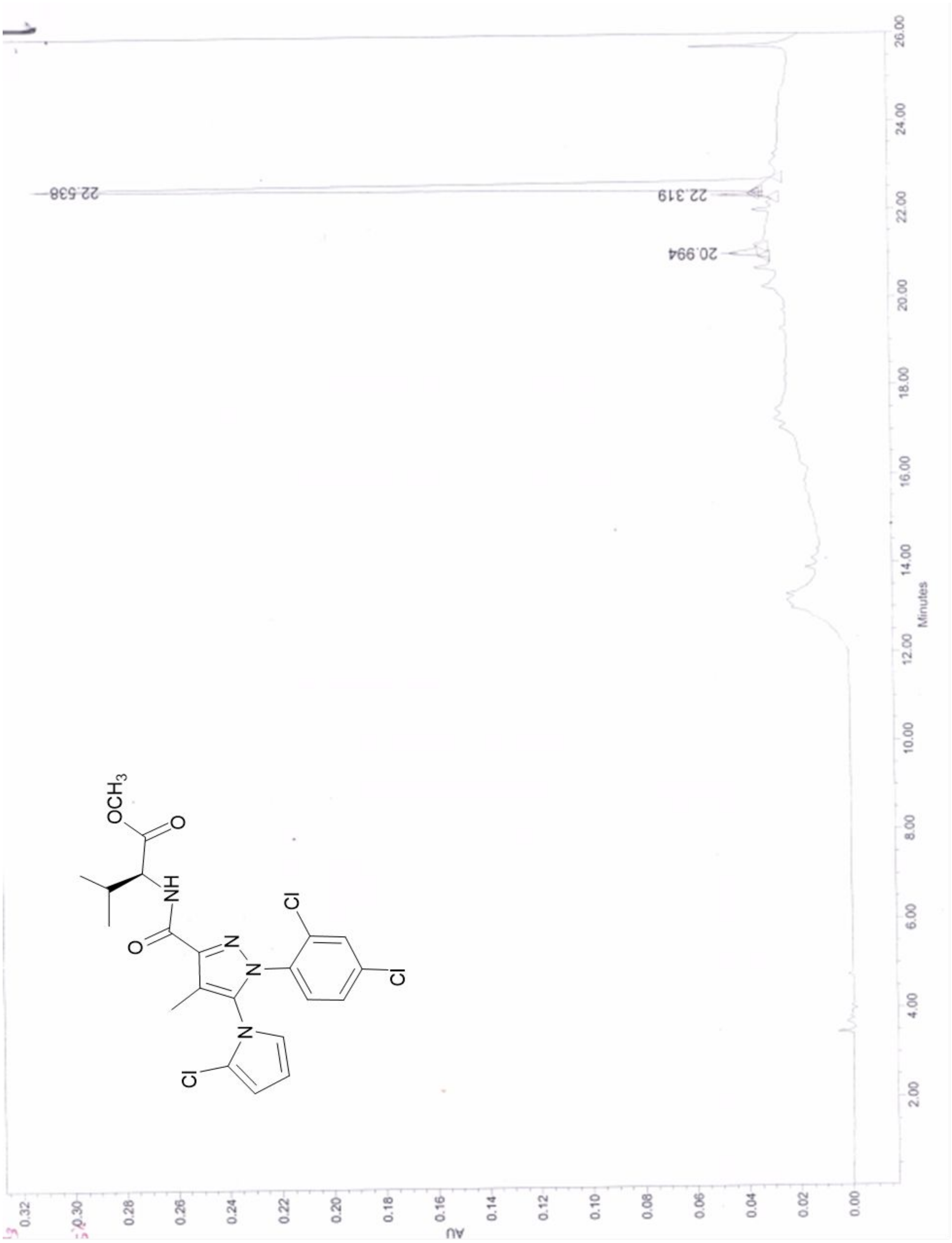

30

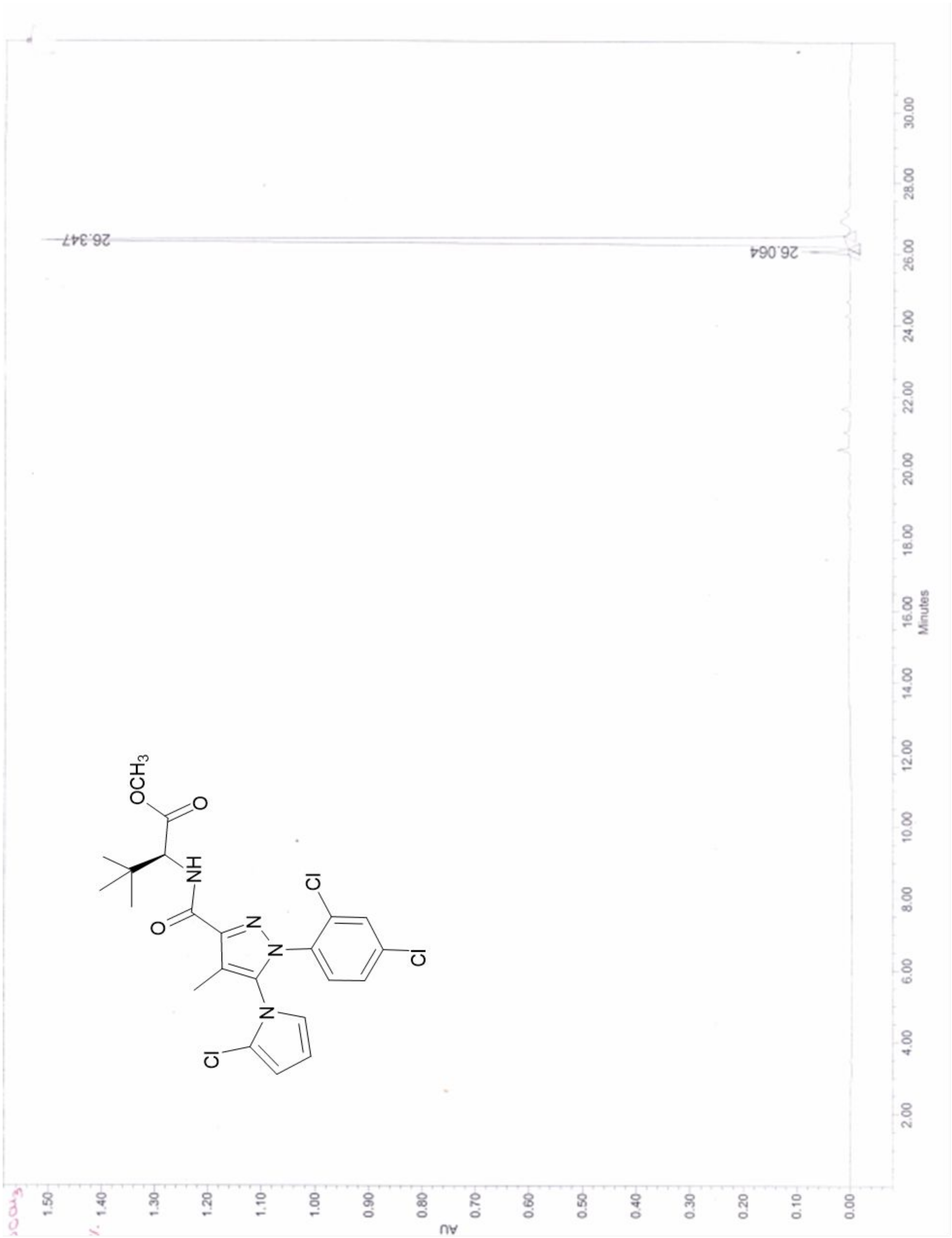

31

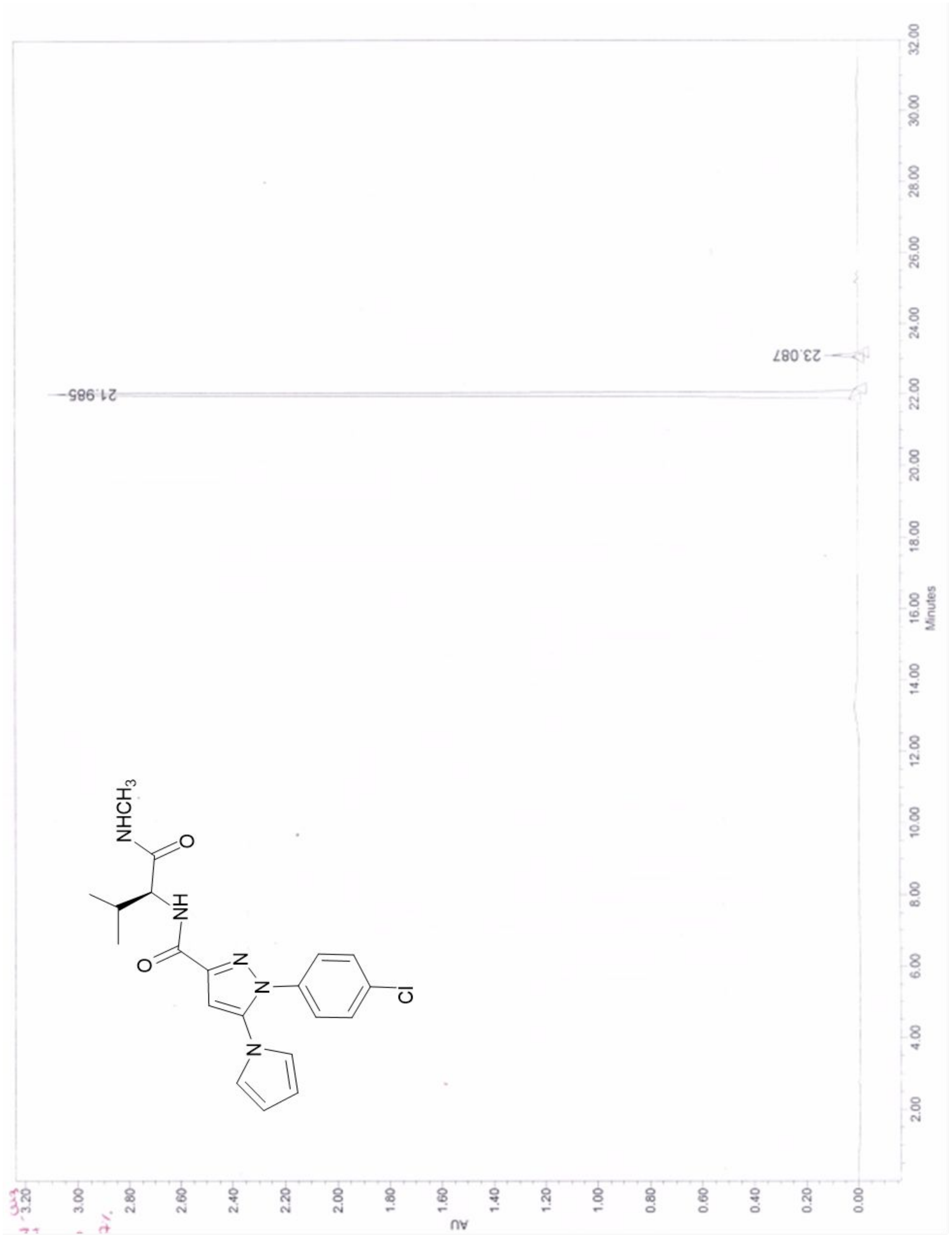

32

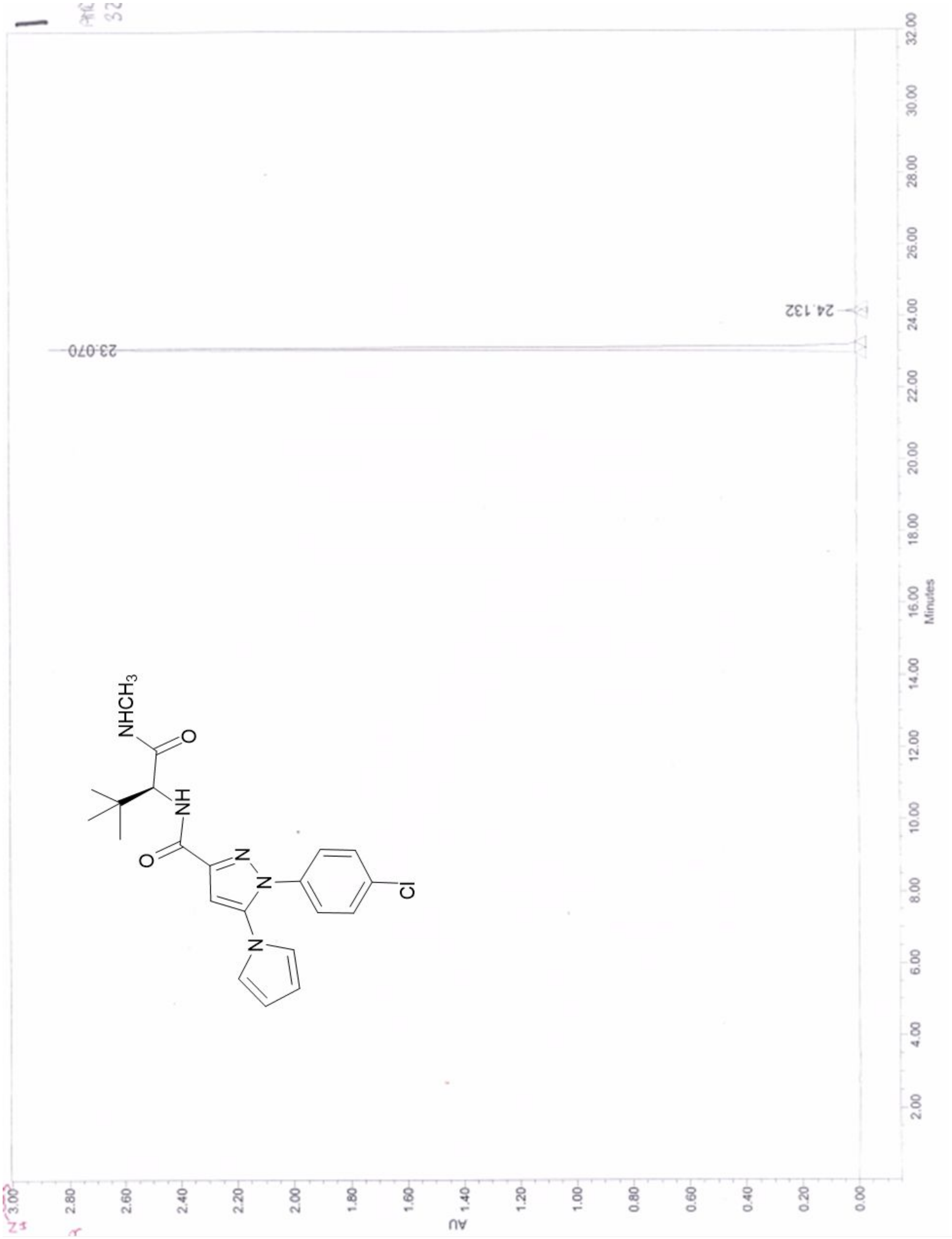

33

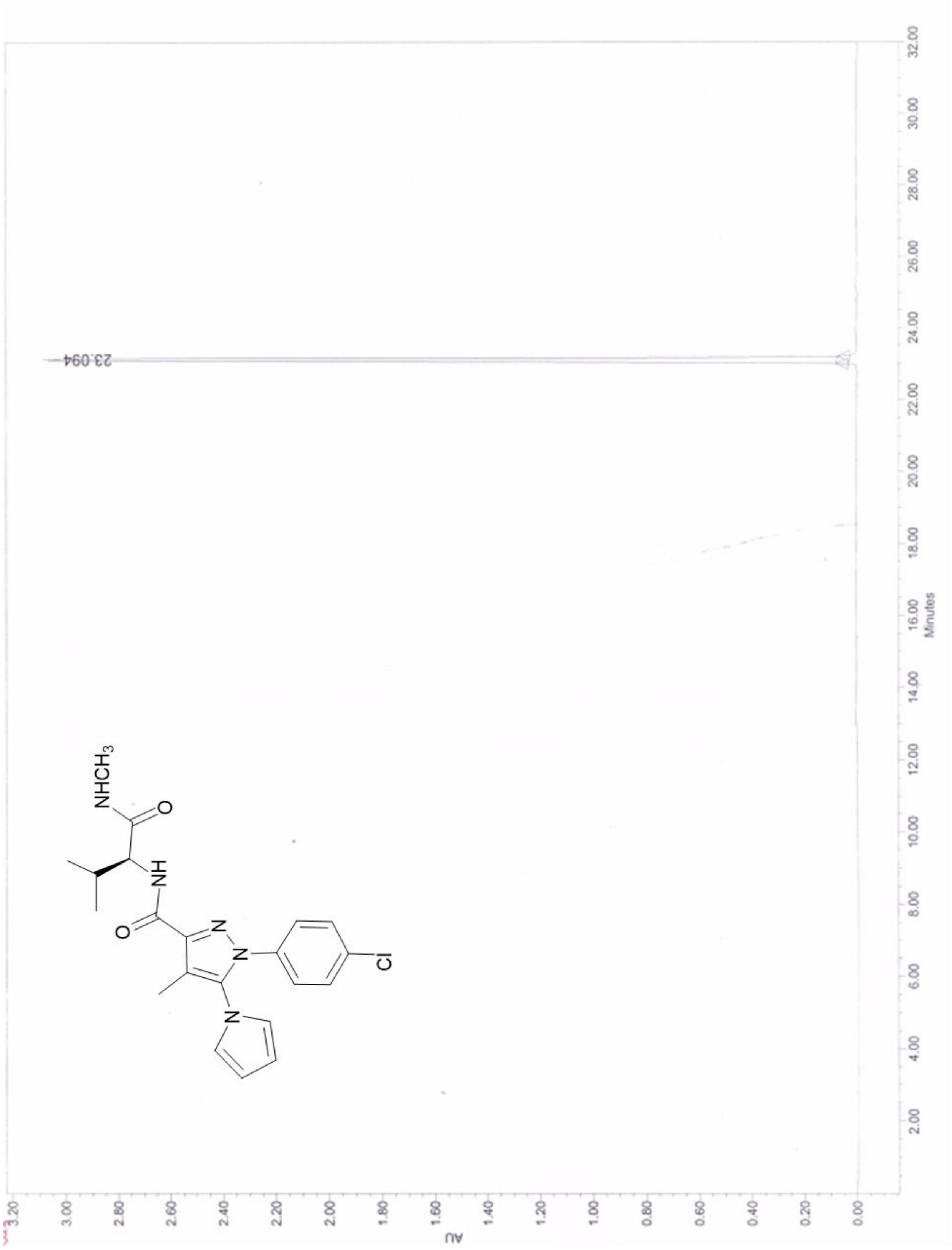

34

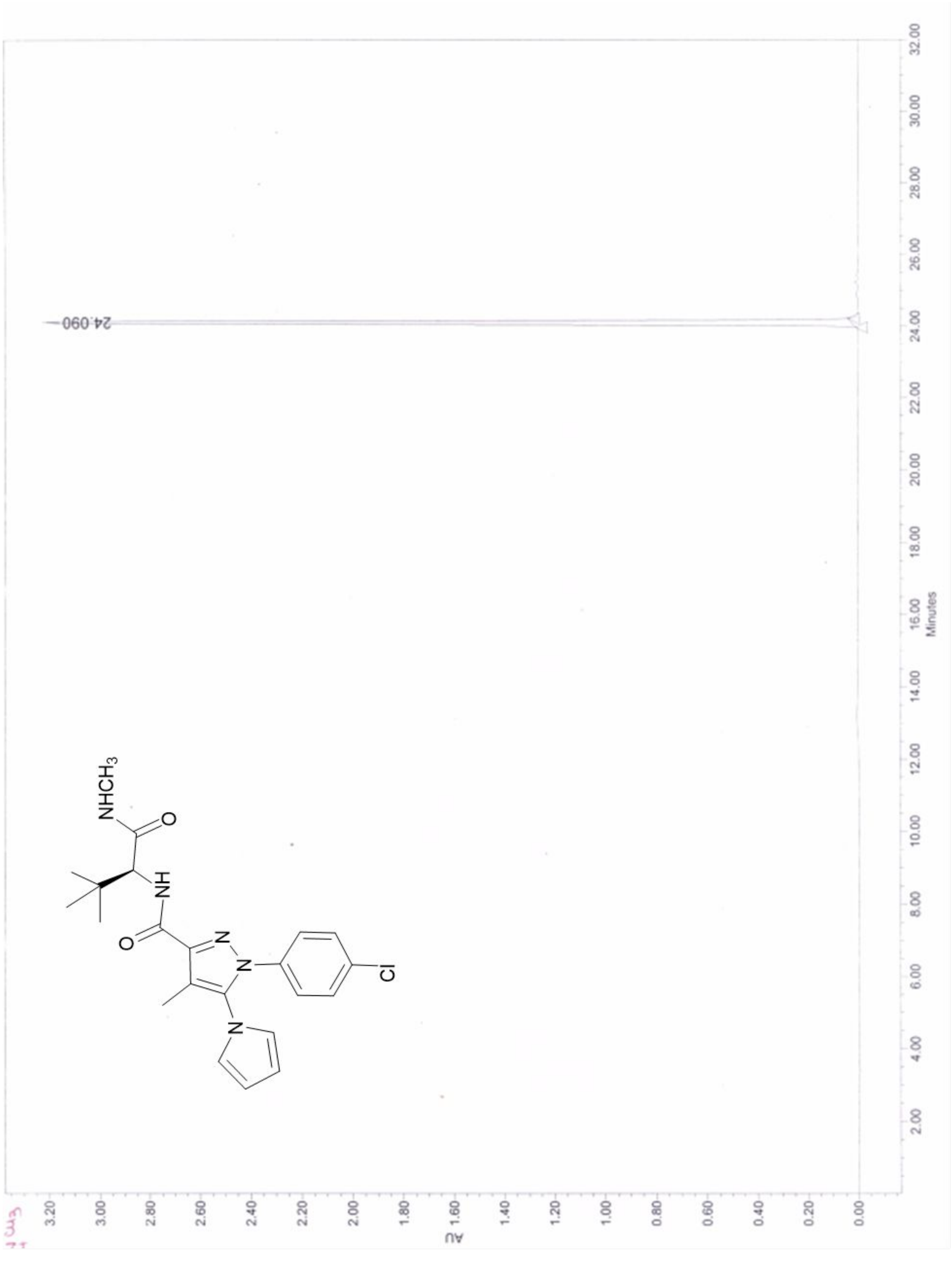

35

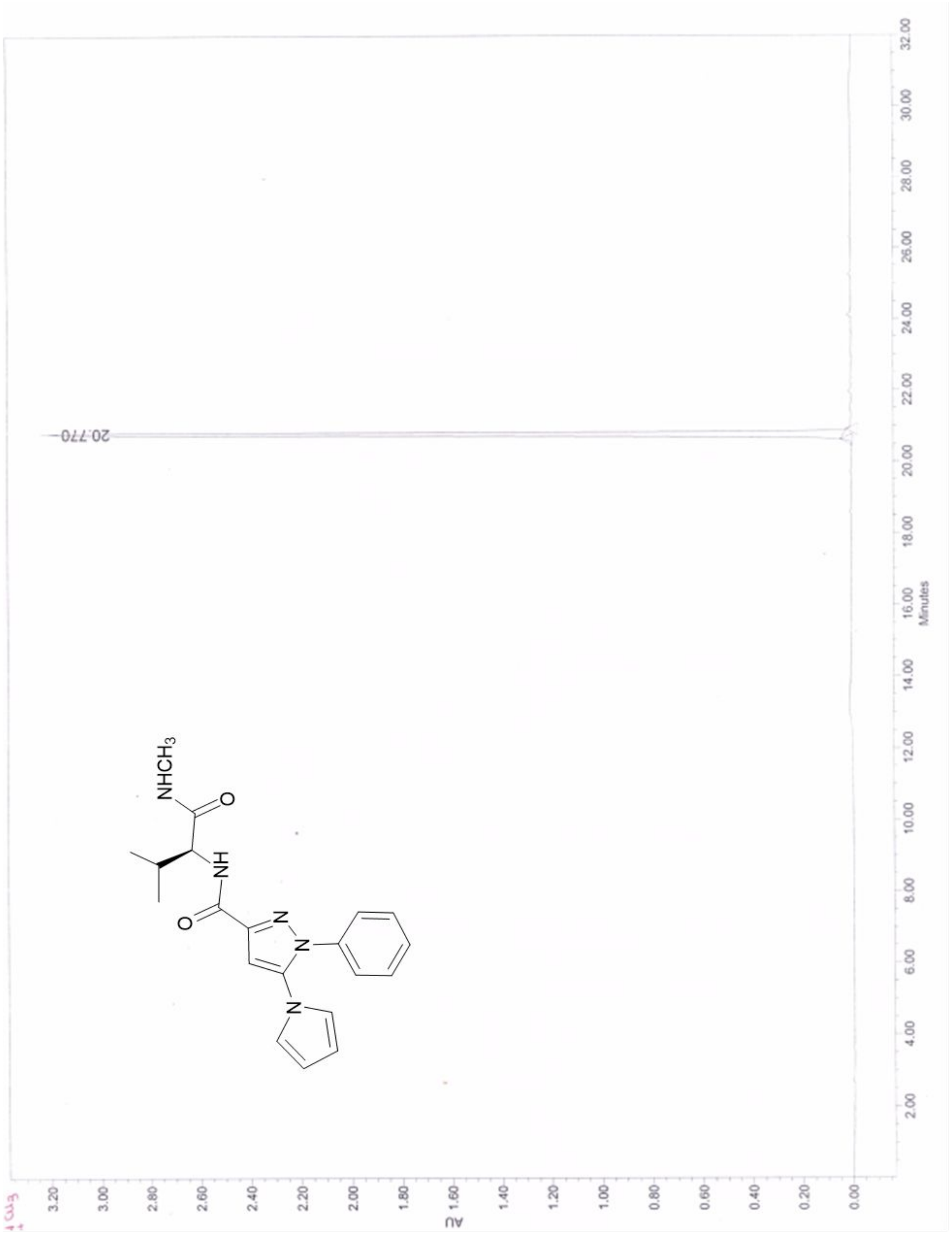

36

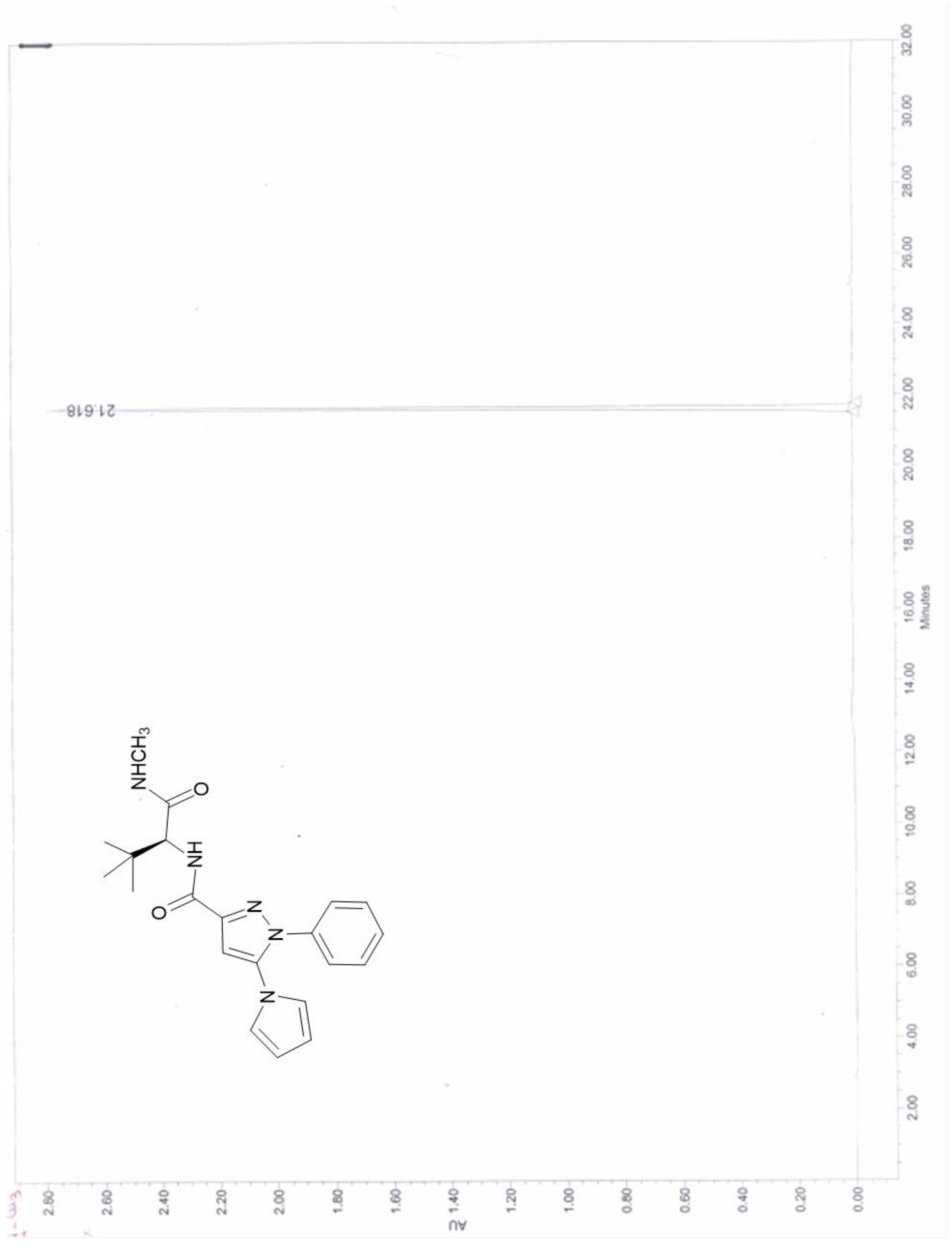

37

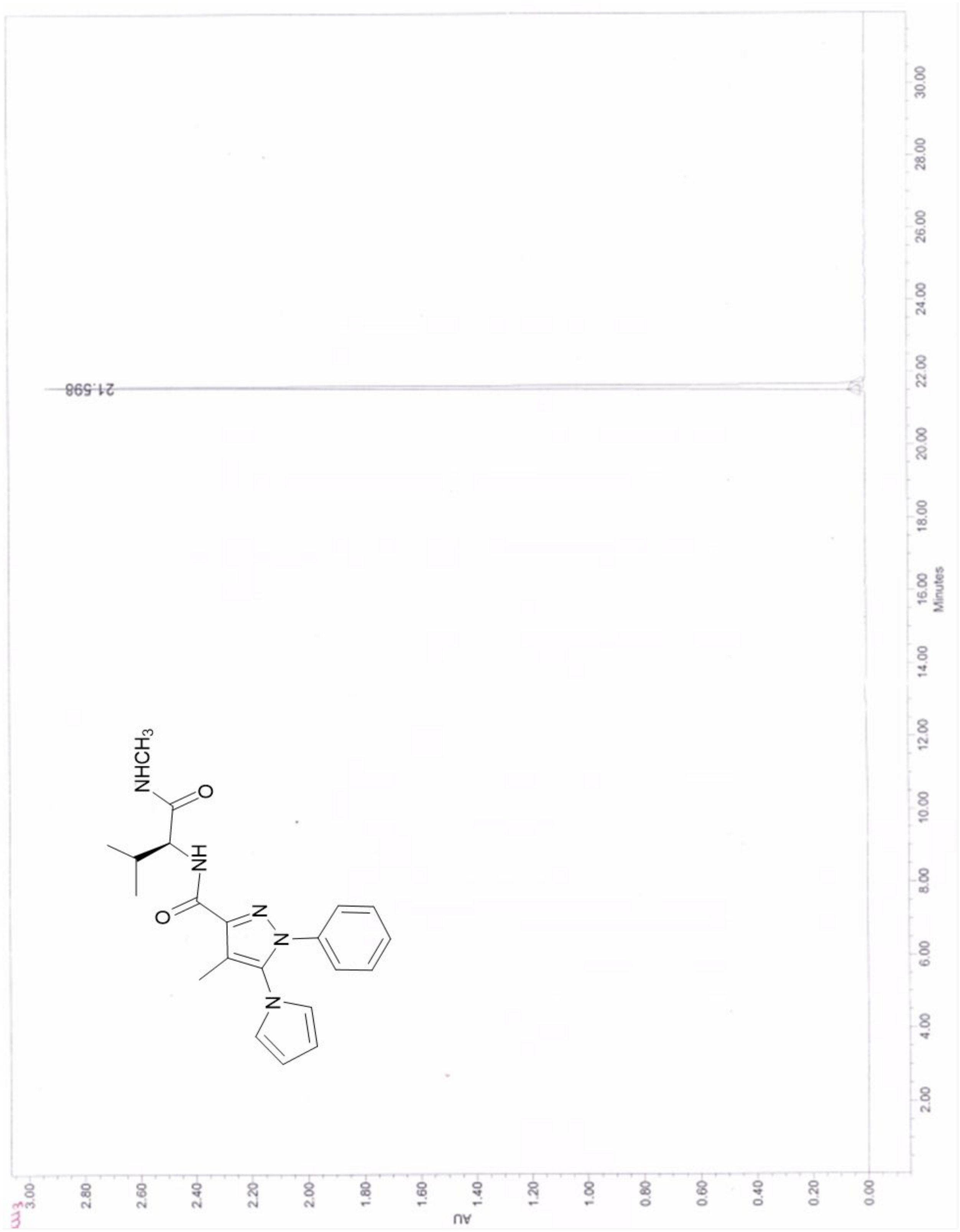

38

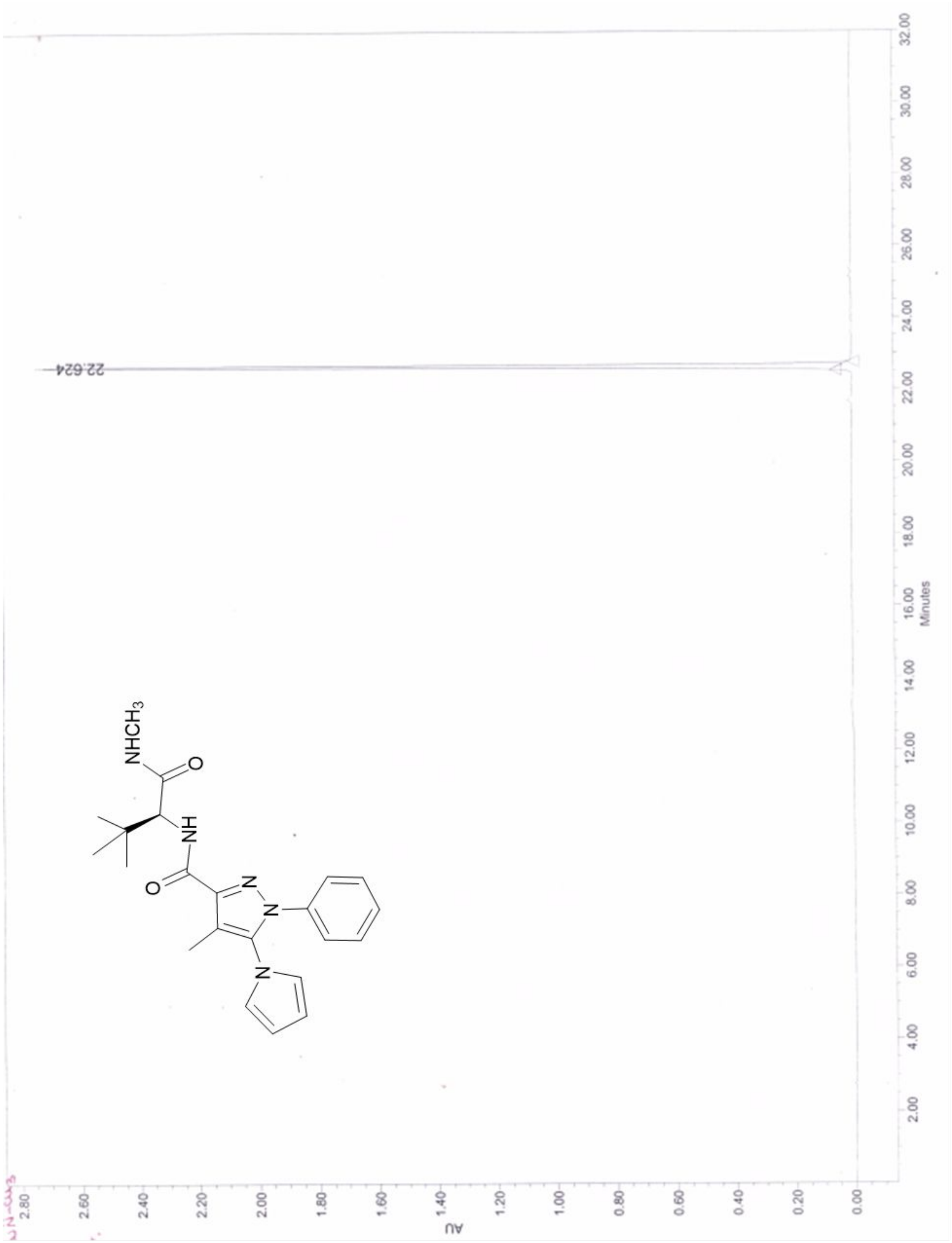

39

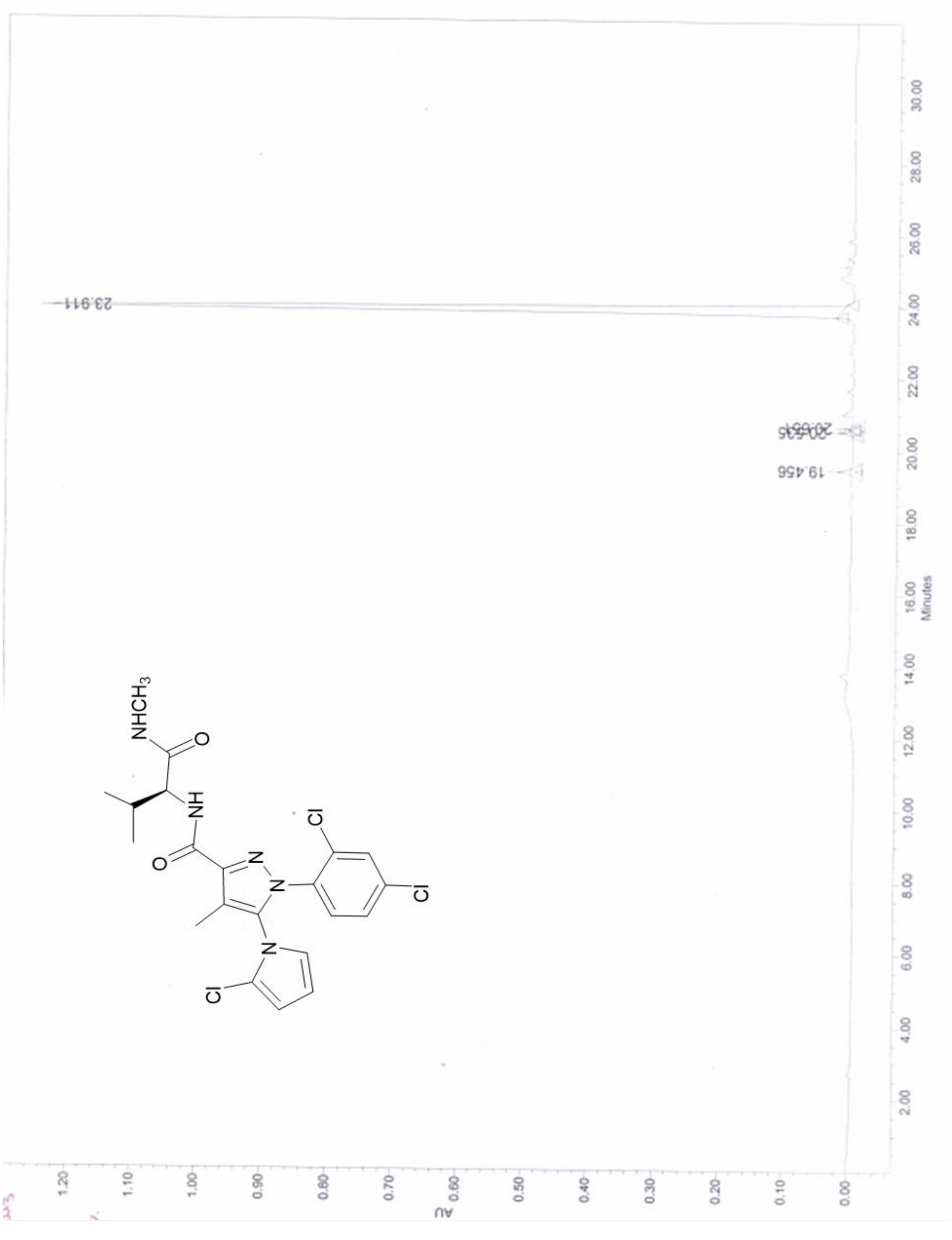

40

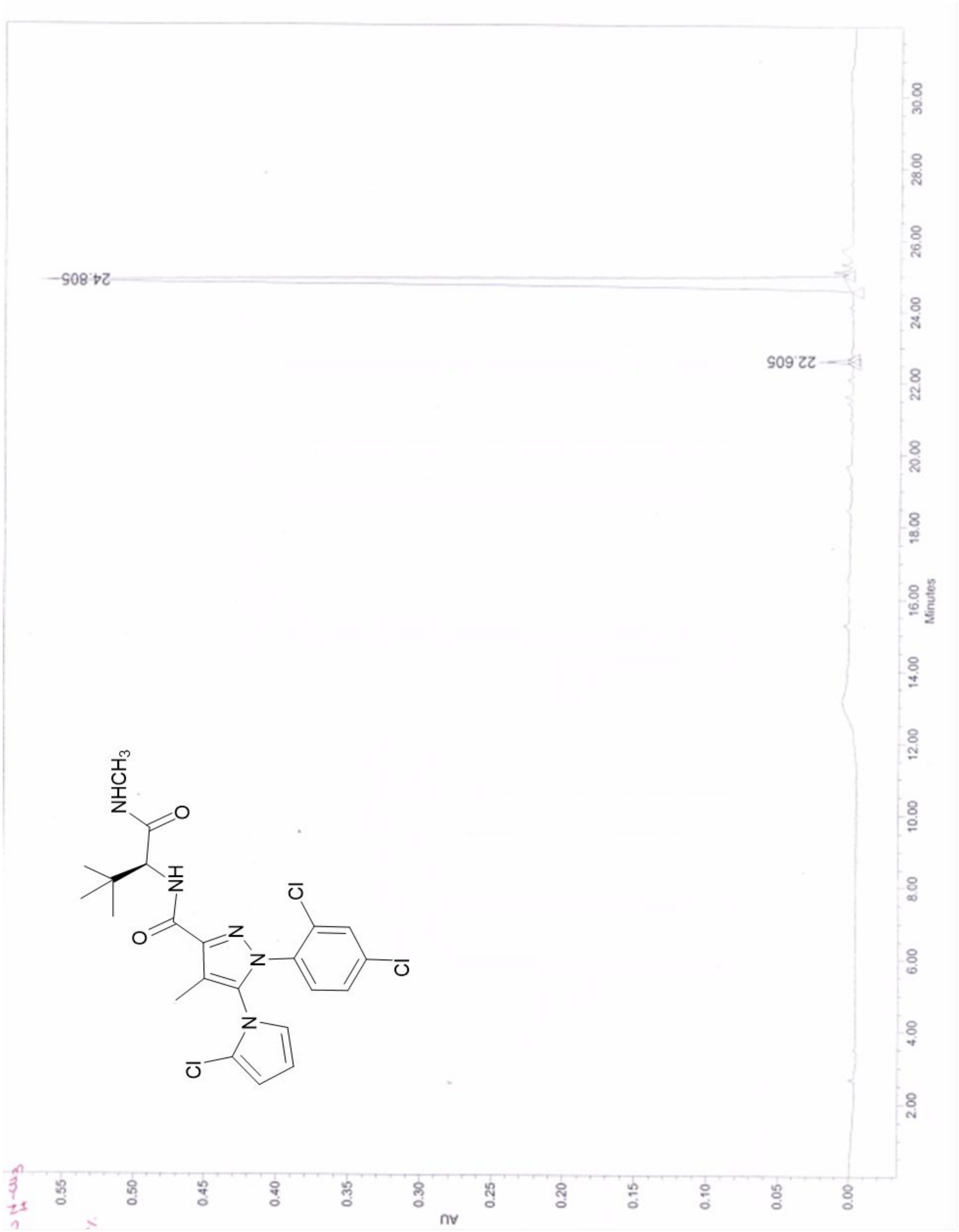

Figure S3. SWISS ADME prediction

Fill with an example | Clear | Run!

Hide BOILED-Egg

Retrieve data: POWERED BY ChemAxon

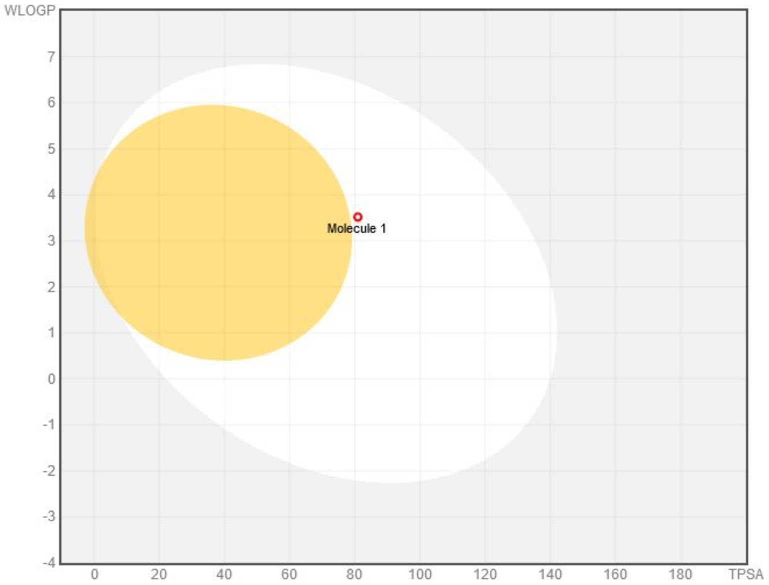

Actions

Show Molecules Name

Legends

BBB

HIA

PGP+

PGP—

Remarks

None

Molecule 1

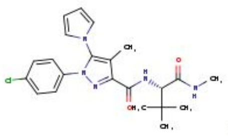

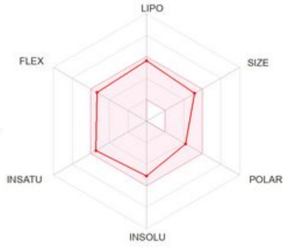

SMILES CNC(=O)[C@H](C(C)(C)C)NC(=O)c1nn(c(c1C)n1cccc1)c1ccc(cc1)Cl

Physicochemical Properties	
Formula	C22H26ClN5O2
Molecular weight	427.93 g/mol
Num. heavy atoms	30
Num. arom. heavy atoms	16
Fraction Csp3	0.32
Num. rotatable bonds	8
Num. H-bond acceptors	3
Num. H-bond donors	2
Molar Refractivity	117.45
TPSA	80.95 Å²

Lipophilicity	
Log $P_{o/w}$ (iLOGP)	3.77
Log $P_{o/w}$ (XLOGP3)	4.33
Log $P_{o/w}$ (WLOGP)	3.52
Log $P_{o/w}$ (MLOGP)	2.75
Log $P_{o/w}$ (SILICOS-IT)	2.85
Consensus Log $P_{o/w}$	3.44

Water Solubility	
Log *S* (ESOL)	-5.09
Solubility	3.50e-03 mg/ml ; 8.17e-06 mol/l
Class	Moderately soluble
Log *S* (Ali)	-5.74
Solubility	7.71e-04 mg/ml ; 1.80e-06 mol/l
Class	Moderately soluble
Log *S* (SILICOS-IT)	-6.38
Solubility	1.80e-04 mg/ml ; 4.21e-07 mol/l
Class	Poorly soluble

Pharmacokinetics	
GI absorption	High
BBB permeant	No
P-gp substrate	No
CYP1A2 inhibitor	No
CYP2C19 inhibitor	Yes
CYP2C9 inhibitor	Yes
CYP2D6 inhibitor	No
CYP3A4 inhibitor	Yes
Log K_p (skin permeation)	-5.84 cm/s

Druglikeness	
Lipinski	Yes; 0 violation
Ghose	Yes
Veber	Yes
Egan	Yes
Muegge	Yes
Bioavailability Score	0.55

Medicinal Chemistry	
PAINS	0 alert
Brenk	0 alert
Leadlikeness	No; 3 violations: MW>350, Rotors>7, XLOGP3>3.5
Synthetic accessibility	3.74
